# A palladium-catalysed multicomponent coupling approach to conjugated poly(1,3-dipoles) and polyheterocycles

**DOI:** 10.1038/ncomms8411

**Published:** 2015-06-16

**Authors:** David C. Leitch, Laure V. Kayser, Zhi-Yong Han, Ali R. Siamaki, Evan N. Keyzer, Ashley Gefen, Bruce A. Arndtsen

**Affiliations:** 1 Department of Chemistry, McGill University, 801 Sherbrooke Street West, Montreal, Quebec, Canada H3A 0K8

## Abstract

Conjugated polymers have emerged over the past several decades as key components for a range of applications, including semiconductors, molecular wires, sensors, light switchable transistors and OLEDs. Nevertheless, the construction of many such polymers, especially highly substituted variants, typically involves a multistep synthesis. This can limit the ability to both access and tune polymer structures for desired properties. Here we show an alternative approach to synthesize conjugated materials: a metal-catalysed multicomponent polymerization. This reaction assembles multiple monomer units into a new polymer containing reactive 1,3-dipoles, which can be modified using cycloaddition reactions. In addition to the synthetic ease of this approach, its modularity allows easy adaptation to incorporate a range of desired substituents, all via one-pot reactions.

## Abstract (web summary)

The requirement for multistep synthesis can render the fabrication of highly substituted polymers particularly troublesome. Here, the authors take advantage of metal-catalysed multicomponent polymerization to synthesize a large family of such materials with ease from single-pot reactions.

## Graphical abstract

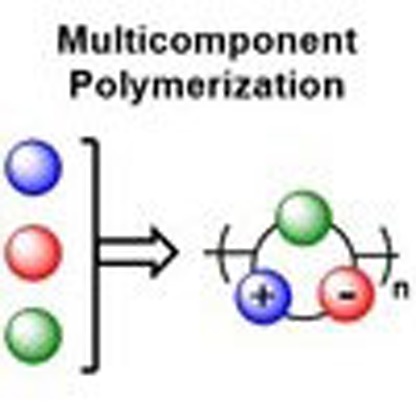

A central goal in polymer synthesis is to directly convert simple chemical building blocks into useful materials. While a wide variety of interesting and potentially important structurally complex polymers have been discovered through recent research efforts (for example, biopolymers, advanced polymer networks, responsive materials and so on), their synthesis via traditional methods can be sufficiently involved to limit their accessibility, especially with the efficiency often demanded in polymer synthesis. One area where structure complexity has proven particularly powerful is in the field of π-conjugated polymers. The development of poly(heterocycles) (polypyrroles^1^, polythiophenes^2^,^3^ and others^4^,^5^,^6^) and their copolymers has sparked a renaissance in how scientists consider constructing a host of organic electronics, such as semiconductors, photovoltaic devices, or sensors^7^,^8^,^9^,^10^. A useful feature of conjugated polymers is their tunability. The modulation of substituents, conjugated heteroatoms or alternating backbone units can allow the construction of conjugated polymers with tailored electronic and other physical features. A number of powerful approaches have been developed to access conjugated polymers, including the now commonplace use of cross-coupling methodologies^11^,^12^. While very effective, these often achieve complexity from the monomers themselves, which can in some instances require a multistep synthesis, followed by halogenation and metallation, and can make accessing varied polymer structures an iterative process. The latter have made the development of alternative methods to construct conjugated polymers an area of growing relevance^13^,^14^,^15^.

In principle, an attractive synthesis of complex conjugated polymers would be to consider their structure as arising directly from available monomers. A challenge is in how to accomplish this in an efficient fashion. One possibility is offered by multicomponent coupling reactions. Multicomponent reactions have been heavily exploited in organic synthesis to increase molecular complexity without the need for multistep synthetic sequences, with high efficiency and minimal waste^16^,^17^. When coupled with transition metal catalysis, these can provide methods to both activate and selectively couple several simple substrates directly into complex products^18^,^19^,^20^. Although metal catalysis is an established tool for activating typically unreactive components towards efficient polymerization (for example, polyolefin synthesis, ring-opening polymerization and so on), the use of metal catalysis to control the coupling of multiple different monomers into new, well-defined and more complex polymer structures is much less explored. Block terpolymer synthesis is well established (**I**; Fig. 1a), and a number of intriguing examples have recently emerged in the metal-catalysed assembly of alternating multicomponent polymers **II** (refs 21, 22, 23, 24, 25, 26, 27), including the synthesis of conjugated^22^,^23^ and high-molecular-weight materials^27^. However, a method to directly convert multiple simple monomers into an entirely new polymer structure such as **III** has to our knowledge not been reported. Considering the variety of monomers relevant for polymerization (for example, diamines, diacids, alkenes, alkynes and so on), this method could provide an efficient route to access a diverse variety of structurally complex polymers, yet do so through combinations of available substrates, with high efficiency, and with facile access to structural diversity.

A polymerization as in Fig. 1a requires a multicomponent reaction that is selective, high yielding, yet relies upon available monomers. A potential transformation that fulfils these features is the palladium-catalysed synthesis of heterocycles shown in Fig. 1b. We have recently reported a palladium-catalysed route to generate 1,3-dipoles **4** (Münchnones) from imines, acid chlorides and carbon monoxide (CO)^28^,^29^. This reaction proceeds in high efficiency and is equally important with substrates that are all easily accessible for a polymerization: imines, acid chlorides and CO. 1,3-Dipoles, including di-Münchnones, have been used in condensation chemistry to synthesize cross-conjugated materials in low molecular weights (Fig. 1c; refs 30, 31). A more attractive approach suggested by this palladium-catalysed synthesis would be to couple simple diimines, diacid chlorides and CO into a novel type of mesoionic, 1,3-dipole-containing conjugated polymer **4**. Münchnones are known to undergo a variety of 1,3-dipolar cycloaddition reactions with unsaturated substrates (A≡B) to generate nitrogen-containing heterocycles^32^. As such, this platform would allow the assembly of various structurally distinct polymers from multiple combinations of available substrates.

We describe here our efforts towards the development of such a metal-catalysed multicomponent polymerization reaction. This has allowed the assembly π-conjugated poly(heterocycles) from multiple combinations of simple monomers: diimines, diacid chlorides, CO, alkenes and/or alkynes. In addition to demonstrating the feasibility of this strategy, this reaction allows access to a new class of conjugated polymer in the form of a mesoionic poly(1,3-dipole). The polymers can be easily modified by cycloaddition, providing access to families of conjugated materials in one-pot, metal-catalysed reactions.

## Results

### Multicomponent synthesis of conjugated polypyrroles

As model monomers for the polymerization, we examined the palladium-catalysed coupling of terephthaloyl chloride **1a**, diimine **2a** based on dialkylfluorene and CO (Table 1). The efficiency of this polymerization was determined by reacting **4a** with the commercial alkyne dimethylacetylene dicarboxylate to form the polypyrrole **5a**. Using simple Pd(II) sources, with or without the addition of phosphines ligands, leads to no polymerization (entries 1–4), presumably owing to their slow reduction under the mild reaction conditions. Conversely, the common Pd(0) catalyst Pd_2_dba_3_·CHCl_3_ (dba=dibenzylidene acetone) results in the rapid conversion of starting materials to form a poorly soluble, amorphous product (entry 7). Gel permeation chromotography (GPC) analysis shows that the tetrahydrofuran (THF) soluble fraction contains a polymer (*M*_n_=7.8 kDa) but with a broad polydispersity (PDI=3.4). The addition of PPh_3_ or PCy_3_ in an attempt to attenuate the reactivity of the palladium catalyst leads instead to complete inhibition of the reaction (entries 5, 8 and 9) while P(*o*-tol)_3_ restores activity but yields a similarly poorly soluble product with a broad PDI (entry 10).

The low solubility and broad PDI in this product may be the alkene-based dba on the palladium precatalyst, which could react via cycloaddition with **4a** and lead to crosslinking. To circumvent this reaction, Pd[P(*o*-tol)_3_]_2_ was used as a commercially available source of Pd(0) with only weakly associated, and unreactive, ligands. This catalyst leads to the near-complete consumption of the reagents under mild conditions (50 °C, 4 atm CO), and the generation of a polymer (**5a**) that is soluble in common solvents (entry 11). ^1^H and ^13^C nuclear magnetic resonance (NMR) and infrared (IR) analysis show the conversion of the monomers into the polypyrrole **5a**, which has identical spectral features to independently prepared model compounds (Supplementary Figs 5 and 16). GPC analysis shows **5a** to have a well-defined monomodal molecular weight distribution (PDI=1.6). The catalytic activity of Pd[P(*o*-tol)_3_]_2_ can be enhanced by the addition of CuPF_6_, which presumably acts as a phosphine scavenger to generate a mono-ligated palladium catalyst (entry 12). Alternatively, simply using high CO pressure leads to a rapid and near-quantitative polymerization, forming polypyrrole **5a** as the only observable product (entry 13). Molecular weights as high as 22.7 kDa can be obtained by increasing the concentration and reaction time (entry 14).

### Synthesis of poly(1,3-dipoles)

The reaction in Table 1 provides a new approach to synthesize pyrrole-based conjugated polymers from combinations of substrates that are either available or monomers themselves in other polymerizations (terephthaloyl chloride, CO, dialdehydes and alkynes). This platform can also be used to access new classes of conjugated materials. For example, the first step in the transformation in Table 1 also generates the 1,3-dipole-containing polymer **4a**. 1,3-Dipoles such as Münchnones are typically considered reactive intermediates and used *in situ* in synthesis. However, while performing the palladium-catalysed coupling in the absence of alkyne, we noted the precipitation of a dark solid **4a** that can be easily isolated by washing with acetonitrile. This polymer is surprisingly stable (<5% mass loss at up to 180 °C under nitrogen), and can be stored at low temperature in the absence of air and moisture, although it does hydrolyse in the presence of water. In order to fully characterize the moderately soluble **4a** by NMR analysis, it was prepared in moderate molecular weight with an imine end-capping agent (**4a′**, *M*_n_=6.7 kDa; Supplementary Methods). Spectral analysis show all the signals for a Münchnone, including characteristic carbonyl resonances in the infrared (1,710 cm^−1^) spectra, in the ^13^C NMR (*δ* 160.6) and others, all of which correlate with the model di-Münchnone **4a′′** prepared from diimine **2a**, toluoyl chloride and CO. Therefore, **4a** represents an unusual new class of mesoionic polymer, poly(1,3-dipoles).

**Figure 2:**
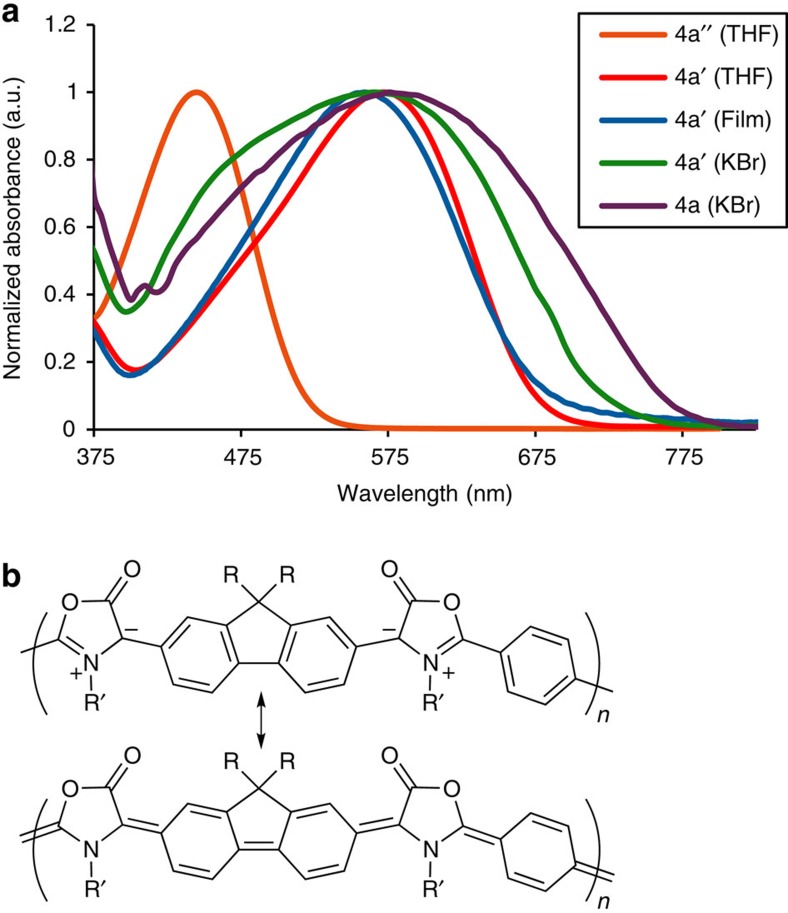
Ultraviolet/visible spectra and conjugation in poly-Münchnones. (**a**) Ultraviolet/visible spectra of **4a** (22.7 kDa), **4a**′ (6.7 kDa) and model dimer 4a′′ (*n*=1). (**b**) Potential resonance structures of **4a** leading to planarization.

Donor/acceptor-conjugated polymers have become an important thrust in the recent design of polymer-based photovoltaic materials^7^,^8^,^33^, although these do not typically incorporate formal charges into the backbone. Likely as a result of this charge separation, poly-Münchnone **4a** is a dark purple solid, characteristic of low-bandgap materials. Ultraviolet/visible analysis shows intense (molar absorptivity up to 5.0 × 10^4^ l mol^−1^ cm^−1^) and broad absorptions in most of the visible region (Fig. 2a). These are significantly red-shifted relative to the model bis-Münchnone, and indicate that **4a** is highly conjugated. While the precise nature of this extended conjugation is still under investigation, the persistent charge-separated character of the mesoionic moieties creates a donor/acceptor motif on a single heterocycle, and may also provide a novel route to planarization as a mechanism to partially eliminate charge (for example, Fig. 2b). Estimation of the optical bandgap by absorbance onset gives values of 1.74 eV for **4a′** (1.59 eV for the high-molecular-weight **4a**), and cyclic voltammetry shows a reversible reduction and an electrochemical bandgap of 1.84 eV. As such, these are a new class of low-bandgap-conjugated materials.

**Figure 3:**
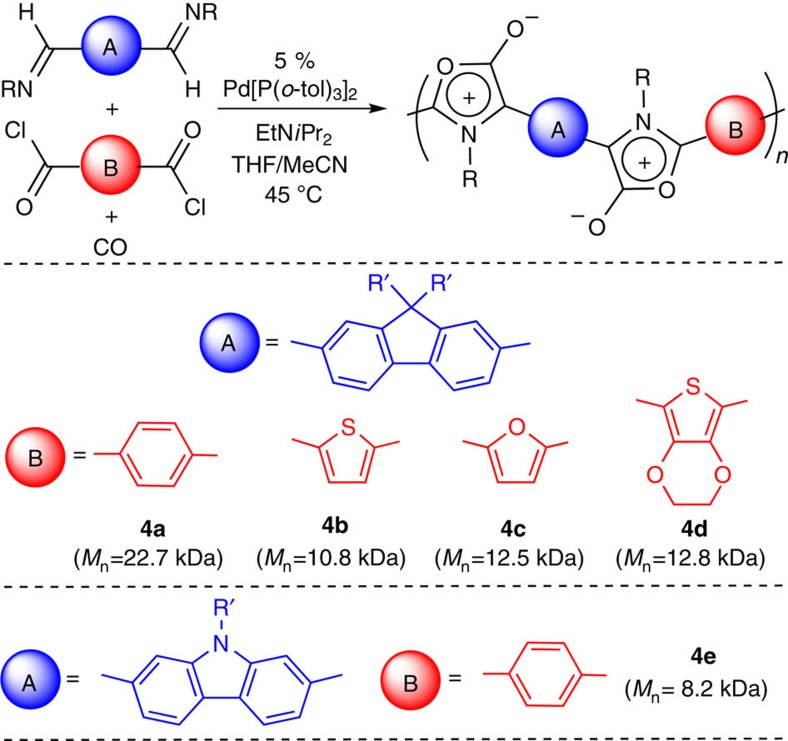
Diversity of Münchnone-containing polymers. Owing to the moisture sensitivity of **4**, molecular weight determined by conversion to polypyrroles **5**, as in Table 1.

In addition to their unusual electronic properties, the synthesis of **4** from diimines and diacid chlorides makes it straightforward to attenuate their structure and form a range of mesoionic polymers. Examples of the structural diversity available are shown in Fig. 3. Notably, each of the bis(acid chloride) monomers are either commercially available or easily prepared from the diacid compounds. For example, 2,5-thiophene dicarbonyl dichloride (precursor to **4c**) can be synthesized in one step from adipic acid, a commodity chemical used in the production of nylon and other polyamides, whereas 2,5-furan dicarboxylic acid is derived from carbohydrates and identified by the US Department of Energy as one of the top 10 bio-based renewable chemicals^34^. The diimines used can be similarly altered to incorporate carbazole (**4e**), a common unit in conjugated polymer production^8^. In all cases, the catalytic coupling is clean and molecular weights are limited only by the solubility of the poly(1,3-dipole).

**Figure 4:**
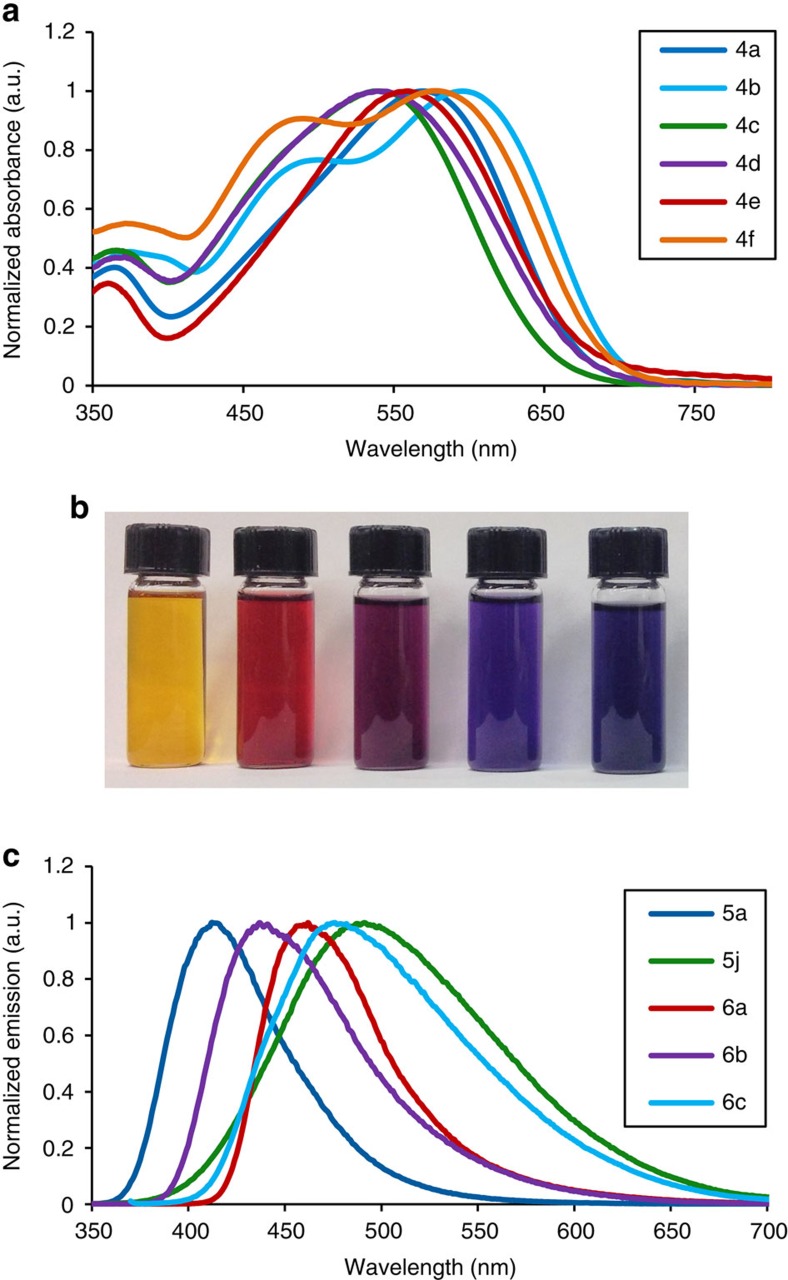
Properties of polymers. Polymers were prepared with imine end-capping at 6–8 kDa to ensure full solubility for analyses (Supplementary Methods). (**a**) Ultraviolet/visible absorbance spectra of poly-Münchnones **4a**–**f** in THF. (**b**) Polymers **5a**, **4f′**, **4e′**, **4a′** and **4c′** in THF (from left to right). (**c**) Fluorescence spectra of select polymers **5–6** in THF.

The structural manipulations translate into the properties of these polymers (Fig. 4a,b). For example, the disubstituted carbazole-containing polymer **4e** displays visible absorbances that are blue-shifted (*λ*_max_ of 539 nm) relative to **4a** (*λ*_max_ of 570 nm; Fig. 4a). Alternatively, the thiophene-containing material **4b** is significantly red-shifted relative to these other polymers (absorbance onset ∼768 nm), corresponding to an optical bandgap of 1.58 eV. The latter is comparable to materials currently of interest as light harvesting materials in bulk heterojunction solar cells^7^,^8^. As such, this provides a method to both construct low-bandgap-conjugated polymers and manipulate or tune their electronic properties by choice of the constituent components.

### Multicomponent synthesis of poly(heterocycles)

**Figure 5:**
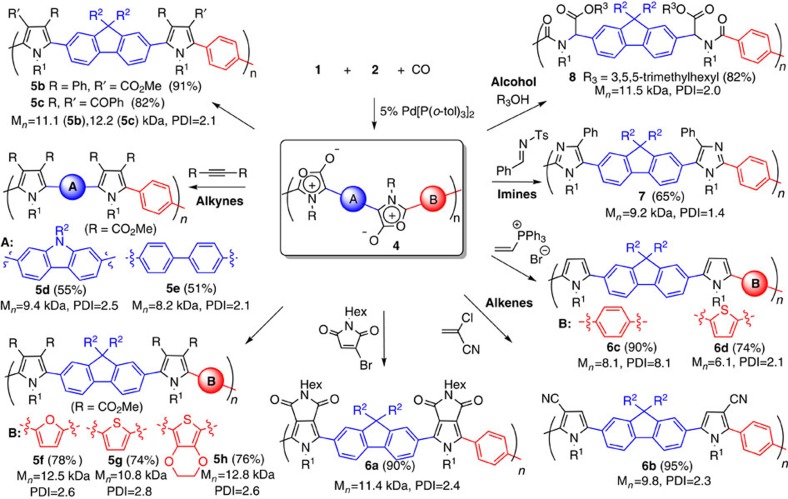
Transformation of poly-Münchnones into families of conjugated polymers. Derivatization performed on poly-Münchnone samples of 8–12 kDa to ensure full solubility of **4a**–**e**, and allow the quantification of the cycloaddition.

**Figure 6:**
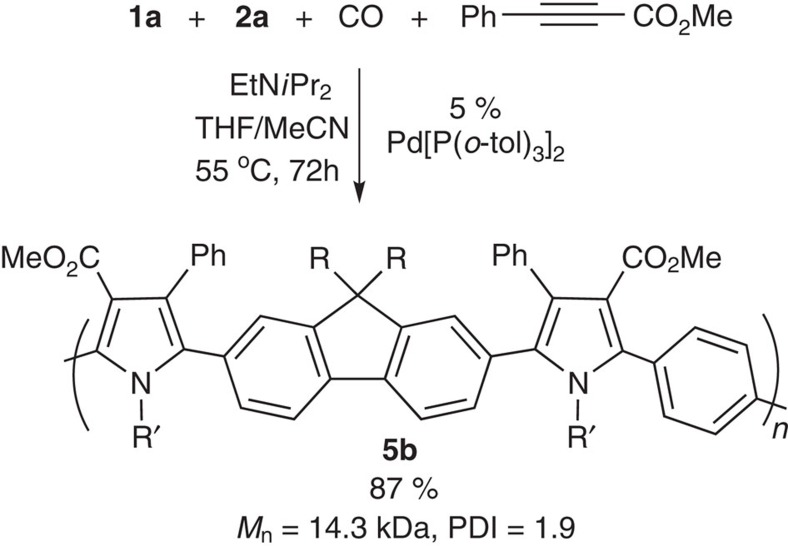
One-pot, four-component polymerization. See Supplementary Methods for experimental details.

These poly-Münchnones **4** also offer access to another deeper level of molecular complexity via their backbone reactivity. This can provide, to our knowledge, a unique route to convert one conjugated organic polymer into other backbone-conjugated polymers^35^,^36^,^37^. For example, the addition of phenyl methylpropiolate to **4a** results in the transformation of the purple, low-bandgap poly-Münchnone into a moderate bandgap, blue-emitting polypyrrole **5b** (Fig. 5). ^1^H NMR and infrared analysis suggest the complete disappearance of 1,3-dipole unit in this reaction, with no observable byproducts. In order to quantify the efficiency of cycloaddition, ^13^C-labelled polymer **4a** was generated from isotopically enriched terephthaloyl chloride 4-C_6_H_4_(^13^COCl)_2_. NMR (^13^C) analysis shows the quantitative reaction with alkyne to form polypyrrole (>95%; see Supplementary Methods for details), and is consistent with the high cycloaddition reactivity of the 1,3-dipoles. A range of pyrrole-based polymers can be formed by this reaction. For example, changing the alkyne used can provide access to a wide variety of pyrrole-based polymers. Representative examples of these include the diester-containing polymer **5a** or the diketone-substituted **5c**. In addition to alkynes, electron-deficient alkenes are suitable dipolarophiles to form pyrroles. This can allow the facile formation of fused ring pyrrole-based polymer **6a** (from cyclic alkene cycloaddition), the mono-substituted **6b** (from chlorocyanoethylene) or the unsubstituted polypyrrole **6c** with high yield. By using different diimines and diacid chlorides, various other polypyrroles can be obtained (for example, **5d**–**5 h**). As such, this provides a platform to readily incorporate desired functionalities onto conjugated polymers, all of which emanate from a single polymer **4**. The reactivity of Münchnones is also not limited to pyrroles. The addition of *N*-tosyl imine forms imidazole-based-conjugated polymer **7**, and backbone conjugation in these polymers can be easily quenched by the addition of alcohols, leading to the generation of polyamides **8**. Poly-Münchnones **4** can therefore be considered highly reactive and versatile conjugated materials, where a single polymer **4** can be transformed into entire families of new conjugated materials. In the case of phenyl methylpropiolate, this polymerization can be performed in a single step (Fig. 6), thereby allowing the orthogonal, four-component synthesis of a conjugated polymer.

This structural diversity can provide a further platform to modulate properties. For example, between polymers **4** and **8**, optical absorbance and polymer bandgap can be tuned across the visible spectrum, and materials can be formed that fluoresce anywhere from blue to green to not at all (Fig. 4c and Table 2; Supplementary Tables 1 and 2 for the full list of properties). Similarly, electrochemical studies show that this cycloaddition (or lack thereof) can be used to tune HOMO energies by over 1 eV. While the primary thrust of these studies was not in product design, several of the polymers reported here display notable properties. Polypyrroles and their copolymers have attracted significant interest as electronic materials^1^,^38^. Three of the pyrrole-based polymers (**5a**, **6a** and **6b**) have good photoluminescent quantum efficiency (>35%) with emission maxima in the range of blue light (413–459 nm). Alternatively, the pyrrole-based imide-functionalized unit has been identified as a promising electron acceptor unit in conjugated polymers, and can be readily generated by this approach (**6a**; ref. 39), while alternating pyrrole-thiophene materials such as **6d** represent new variants of materials found to be of use in field-effect transistors^40^. The range of properties observed is a direct result of their structural diversity, which varies from the backbone-conjugated heterocycles, the spacer units, to the substituents. This level of structural attenuation would require an individual synthesis for each new monomer via typical methods. In this case, each polymer is generated in one pot, from a small pool of monomers, and with minimal waste (often only HCl and CO_2_).

### Synthesis of polymers from vanillin

**Figure 7:**
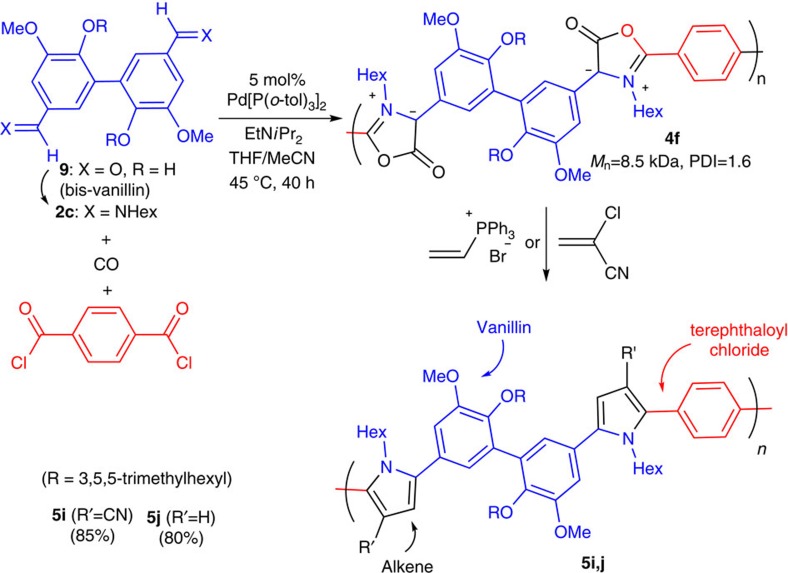
Conjugated polymers from vanillin. Multicomponent synthesis of polymer **4f** and its derivatization to **5i** and **5j**.

Finally, we have examined the potential of using other renewable materials as precursors to conjugated polymers. Lignin is a major component of lignocellulosic biomass and the world's largest renewable source of aromatic compounds, thereby making it a potentially attractive, bio-based feedstock for π-conjugated polymers^41^. Lignin depolymerization yields a variety of aromatic building blocks, including the dialdehyde **9** (a dimer of vanillin). As this multicomponent polymerization uses aldehydes and carboxylic acids as monomer feedstocks, **9** can be incorporated in this palladium-catalysed polymerization to generate the conjugated poly-Münchnone **4f** (Fig. 7). Ultraviolet/visible and electrochemical studies show that **4f** is a moderately low-bandgap polymer (1.9 eV). As above, the dipole in **4f** can undergo cycloaddition reactions to generate the polypyrroles **5i** and **5j**, each of which are blue-emitting materials. These polymers are hybrid materials derived from the following four simple substrates: vanillin, terephthaloyl chloride, a primary amine and CO, and represent, as far as we are aware, the first use of lignin in cross-conjugated polymer formation. Considering the versatility of this reaction, it should prove relevant for the controlled assembly of a range of renewable-based conjugated polymers.

## Discussion

In summary, we have described a new type of metal-catalysed multicomponent polymerization, which provides a method to convert combinations of monomers, such as diimines, diacid chlorides, CO, alkynes, alkenes and alcohols, into structurally well-defined conjugated polymers. In addition, new backbone-conjugated mesoionic polymers (**4**) can be prepared via this coupling, which can undergo efficient post-polymerization cycloaddition to generate families of conjugated materials. In light of the variety of dipolarophiles possible for cycloaddition, as well as diimines and bis(acid chloride)s available, this can be used to construct arrays of conjugated materials, yet without the typical need to preassemble each new conjugated unit, and with the efficiency often desired in polymer synthesis. This multicomponent catalytic polymerization approach could prove equally applicable for the controlled assembly of a range of new materials from established monomers and/or other inexpensive building blocks. Experiments directed towards the latter are currently underway.

## Methods

### General procedure for the synthesis of poly-Münchnones and polyheterocycles

To diacid chloride **1** (0.1 mmol) and diimine **2** (0.100 mmol) in THF (0.6 ml) in a 5-ml vial under N_2_ was added *N*,*N*-diisopropylethylamine (51.7 mg, 70.0 μl, 0.400 mmol), and Pd[P(*o*-tol)_3_]_2_ (7.2 mg, 0.010 mmol) in 1.3 ml THF (1.3 ml)/MeCN (0.6 ml). The vial was placed in a 40-ml Parr steel autoclave, charged with CO (20 bar) and heated to 45 °C. (For the imine endcapped poly-Münchnone, *p*-tolyl(H)C=NC_16_H_37_ was added; see Supplementary Methods for details.) The CO was evacuated, the vessel was brought into a glovebox and the appropriate dipolarophile in 1.5 ml THF was added. The reaction was stirred at room temperature or 50 °C for 16 h, 0.2 ml water was added and the polymer product was extracted with *o*-dichlorobenzene using a Soxhlet extractor. The solvent was removed *in vacuo*, and the residue was dissolved with a minimum amount of hot chloroform (∼1 ml) and added dropwise into methanol (∼20 ml) to precipitate the polymer. The suspension was centrifuged and the methanol layer was decanted. The polymer was washed with methanol (3 × 2 ml) before drying *in vacuo* at 50 °C. See Supplementary Methods for further information. For NMR analysis, ultraviolet/visible, fluorescence and cyclic voltammetry of the molecules in this article, see Supplementary Figs 1–7.

## Additional information

**How to cite this article**: Leitch, D. C. *et al.* A palladium-catalysed multicomponent coupling approach to conjugated poly(1,3-dipoles) and polyheterocycles. *Nat. Commun.* 6:7411 doi: 10.1038/ncomms8411 (2015).

## Supplementary Material

Supplementary Information Supplementary Figures 1-77, Supplementary Tables 1-2, Supplementary Methods and Supplementary References

## Figures and Tables

**Figure 1:**
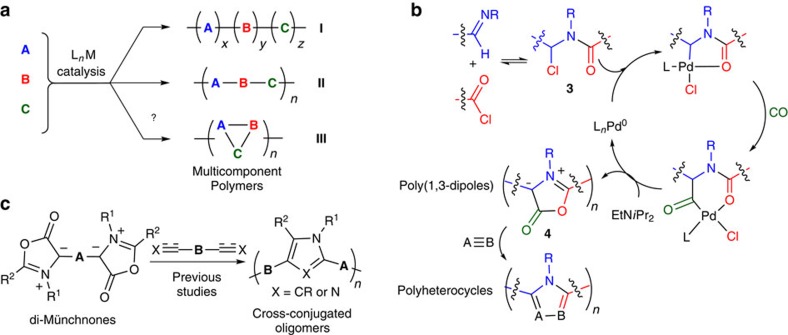
Multicomponent approaches to complex polymer synthesis. (**a**) Metal-catalysed multicomponent coupling approaches to polymers. (**b**) Palladium-catalysed Münchnone formation. (**c**) Previous work involving di-Münchnones in oligomer synthesis.

**Table 1:** A multicomponent synthesis of conjugated polypyrroles*.

**Table 2:** Properties of polymers 5–8.

**Compound**	***λ***_**max**_ **(nm)**	***λ***_**em**_ **(nm)**	***ϕ***_**PL**_	***E***_**g**_^**opt**^**(eV)**	***E***_**g**_^**opt**^ **film (eV)**
**5a**	321	413	0.39	3.18	3.16
**5b**	335	492	0.27	2.91	2.87
**5c**	329	496	0.04	2.99	2.98
**5d**	325	474	0.03	2.92	2.79
**5e**	301	413	0.12	3.15	3.08
**5f**	320	459	0.14	3.08	2.97
**5g**	320	467	0.11	3.07	3.11
**5h**	321	467	0.10	3.05	2.97
**5i**	312	417	0.17	3.19	2.93
**5j**	329	473	0.02	2.88	2.92
**6a**	364	459	0.35	2.86	2.80
**6b**	345	431	0.47	3.01	2.96
**6c**	366	501	0.08	2.60	2.60
**6d**	368	497	0.06	2.63	2.47
**7**	330	467	0.12	2.98	2.80
**8**	280	—	—	3.45	3.42

Select physical properties of **5–8** (see Supplementary Tables 1 and 2 for further details, electrochemical studies, HOMO/LUMO energies and for properties of polymers **4a–f′**).
