# Supplementary material for: A palladium-catalysed multicomponent coupling approach to conjugated poly(1,3-dipoles) and polyheterocycles
Source: Nat Commun. 2015 Jun 16;6:7411. doi: 10.1038/ncomms8411 (PMC4490558; doi:10.1038/ncomms8411)
Supplement: Supplementary Information — Supplementary Figures 1-77, Supplementary Tables 1-2, Supplementary Methods and Supplementary References [file ncomms8411-s1.pdf]

Supplementary Figures

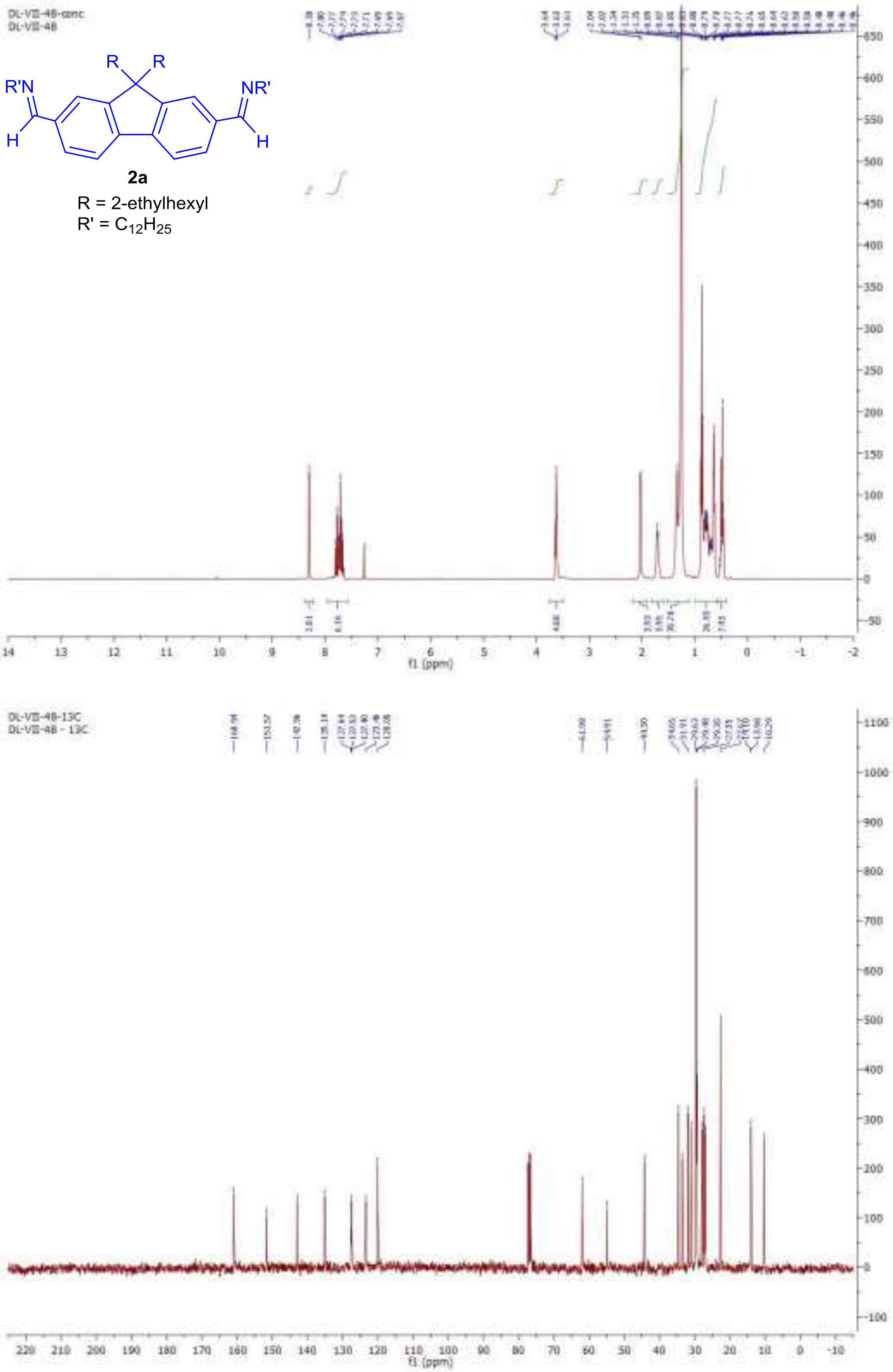

Supplementary Figure 1. ^{1}H and ^{13}C NMR spectra for monomer **2a**.

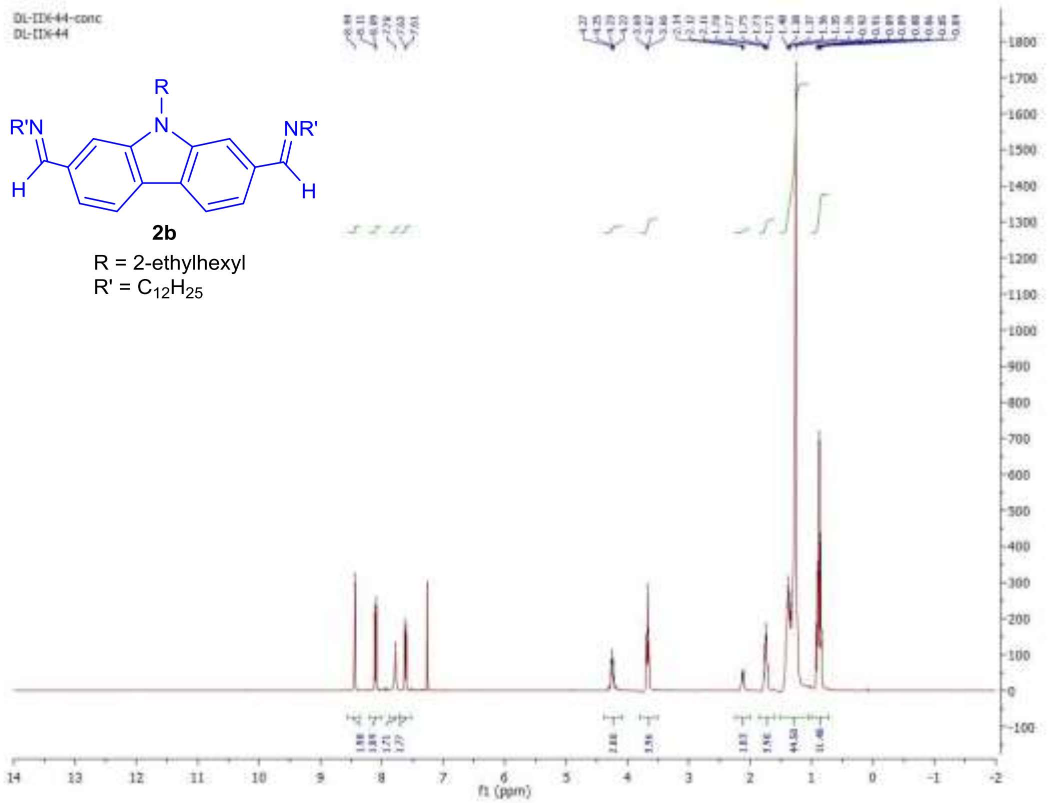

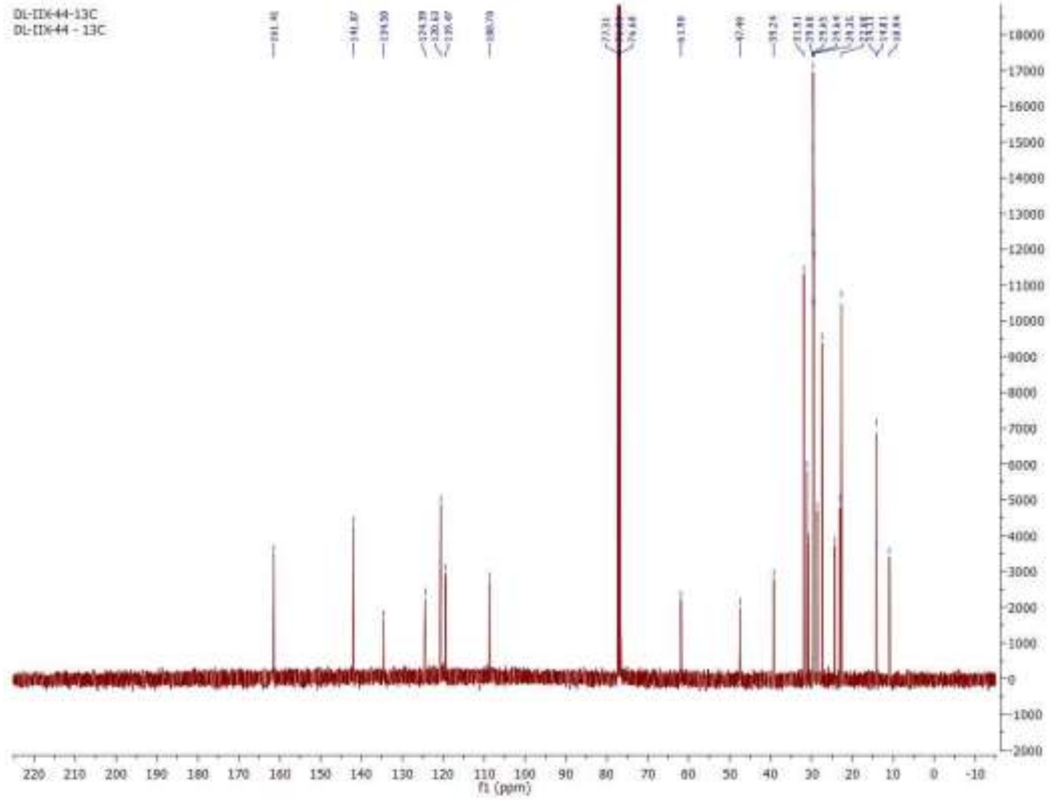

Supplementary Figure 2. ^{1}H and ^{13}C NMR spectra for monomer **2b**.

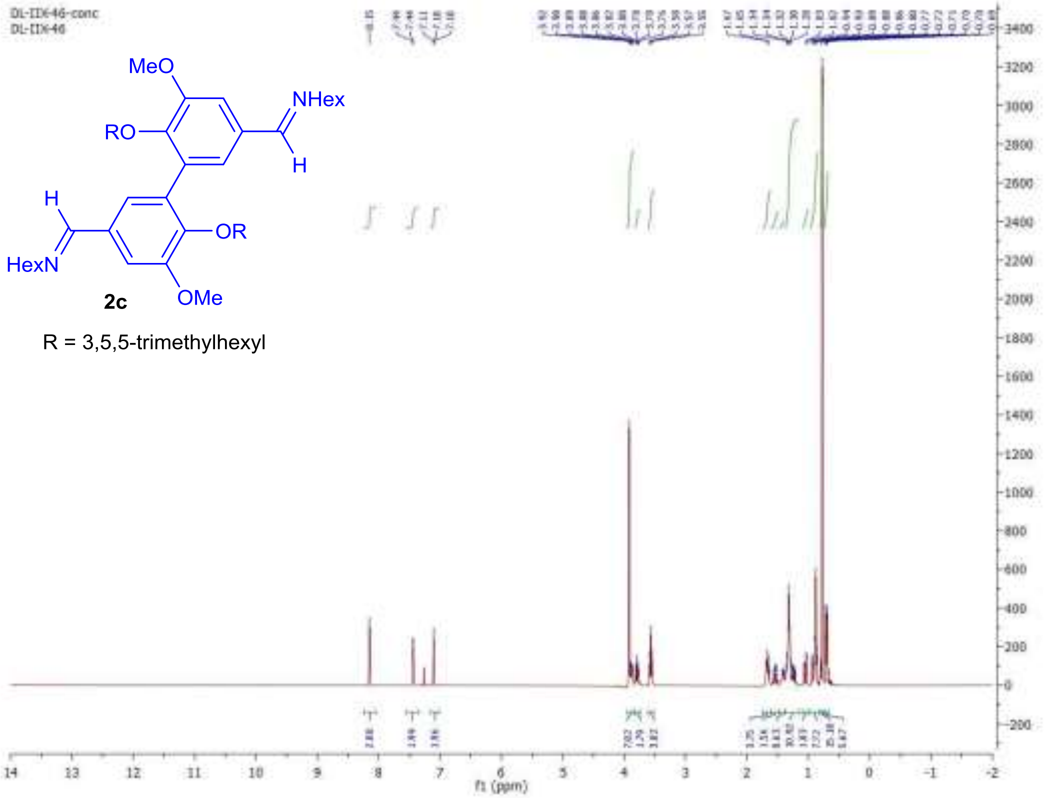

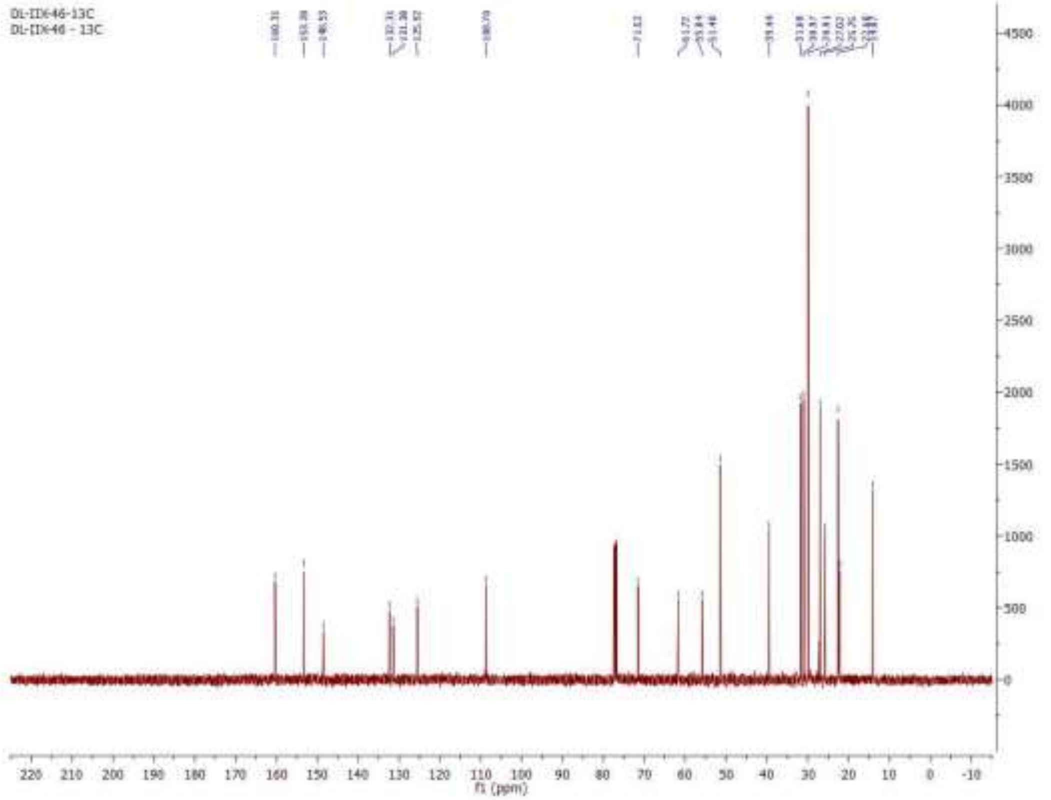

Supplementary Figure 3. ^{1}H and ^{13}C NMR spectra for monomer **2c**.

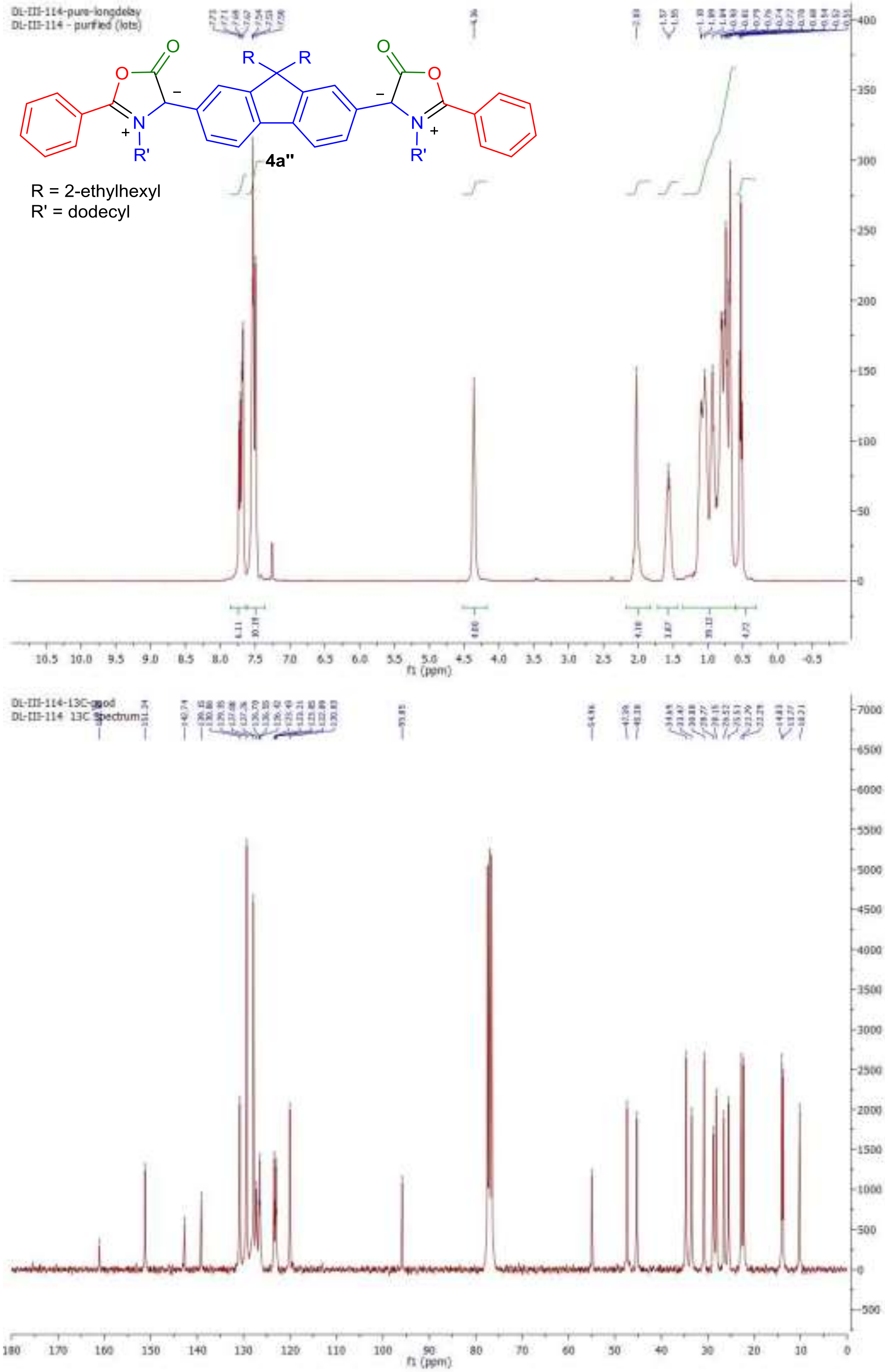

Supplementary Figure 4. ^{1}H and ^{13}C NMR spectra for dimer **4a''**.

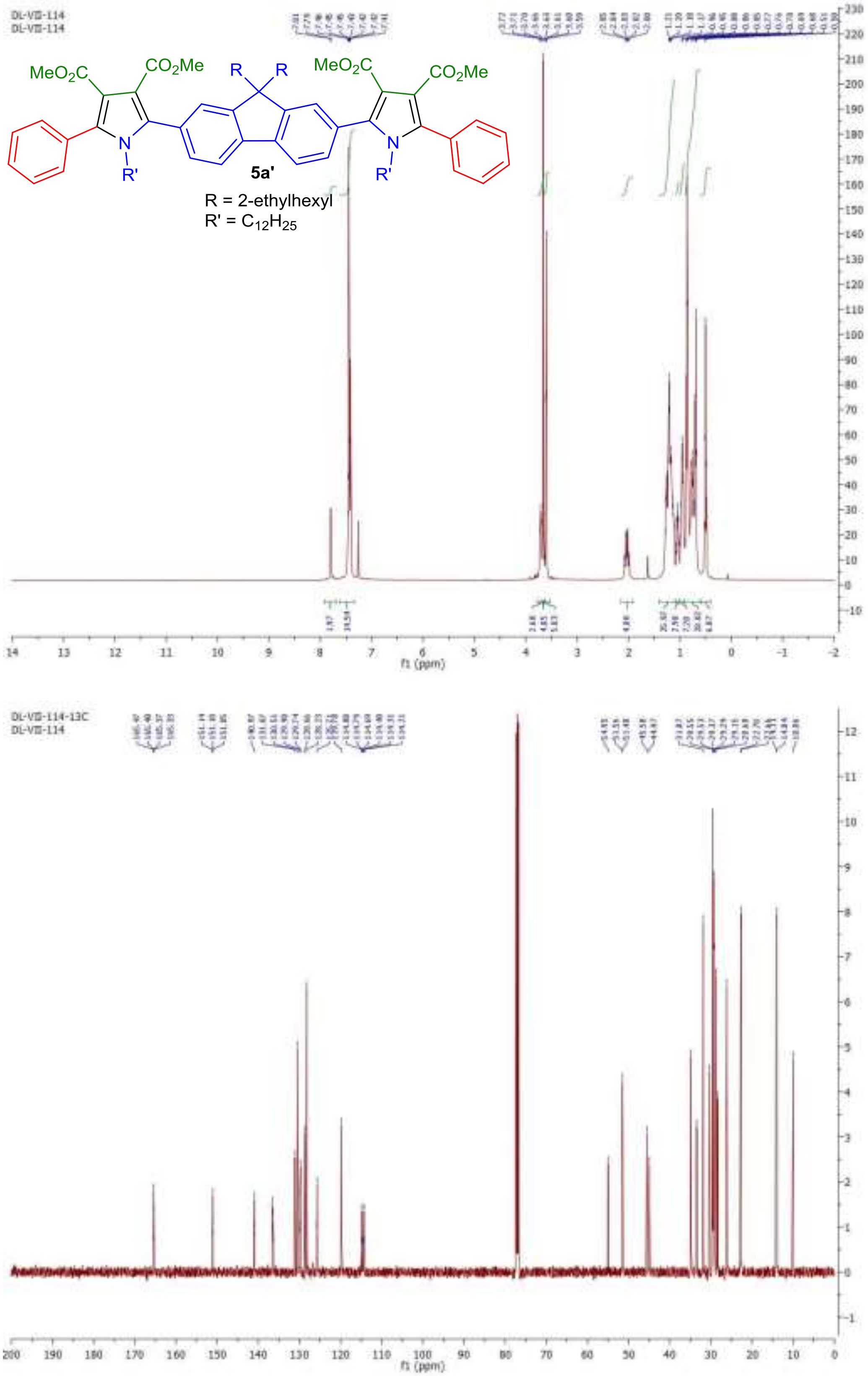

Supplementary Figure 5. ^{1}H and ^{13}C NMR spectra for dimer **5a'**.

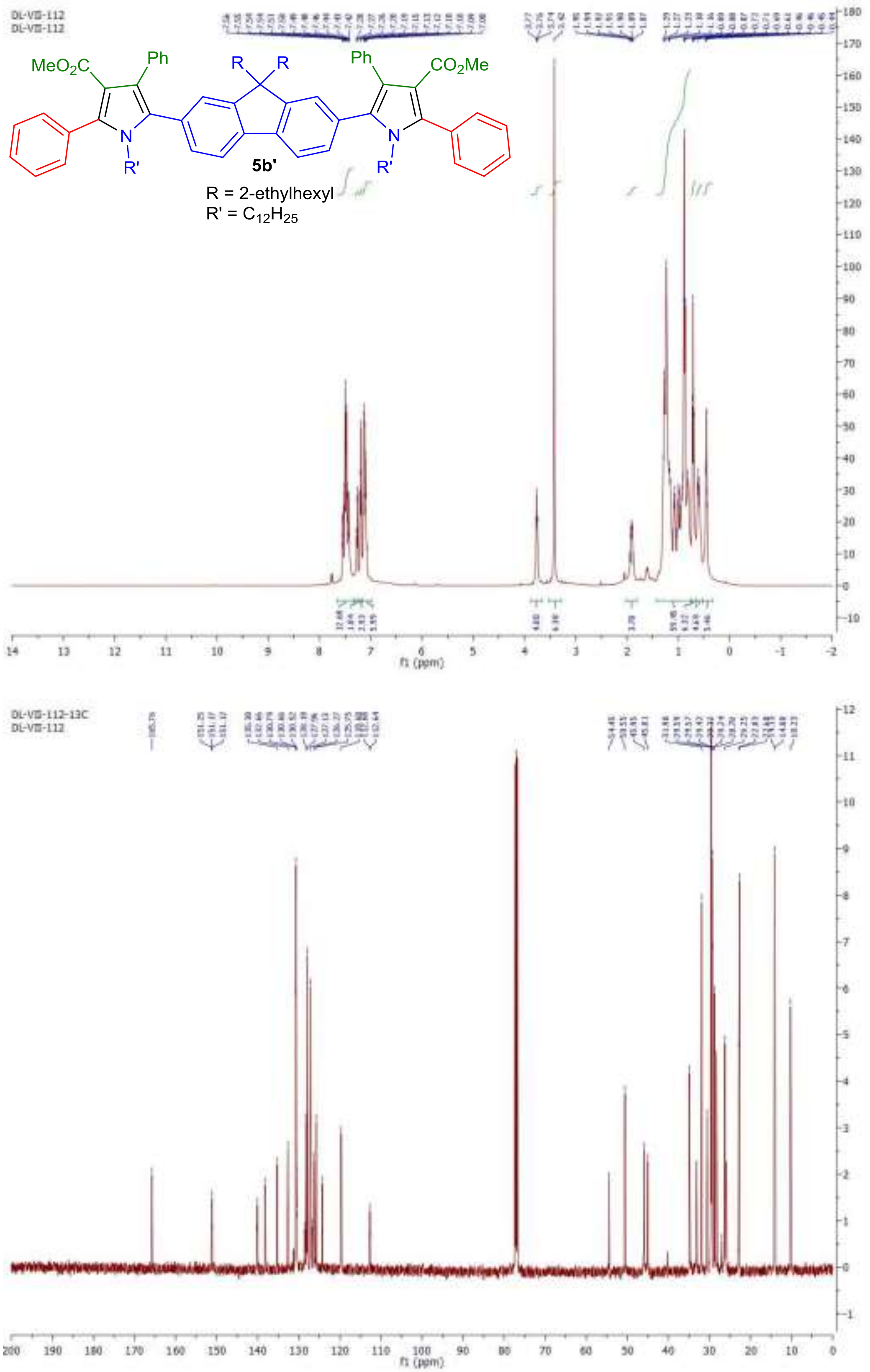

Supplementary Figure 6. ^{1}H and ^{13}C NMR spectra for dimer **5b’**.

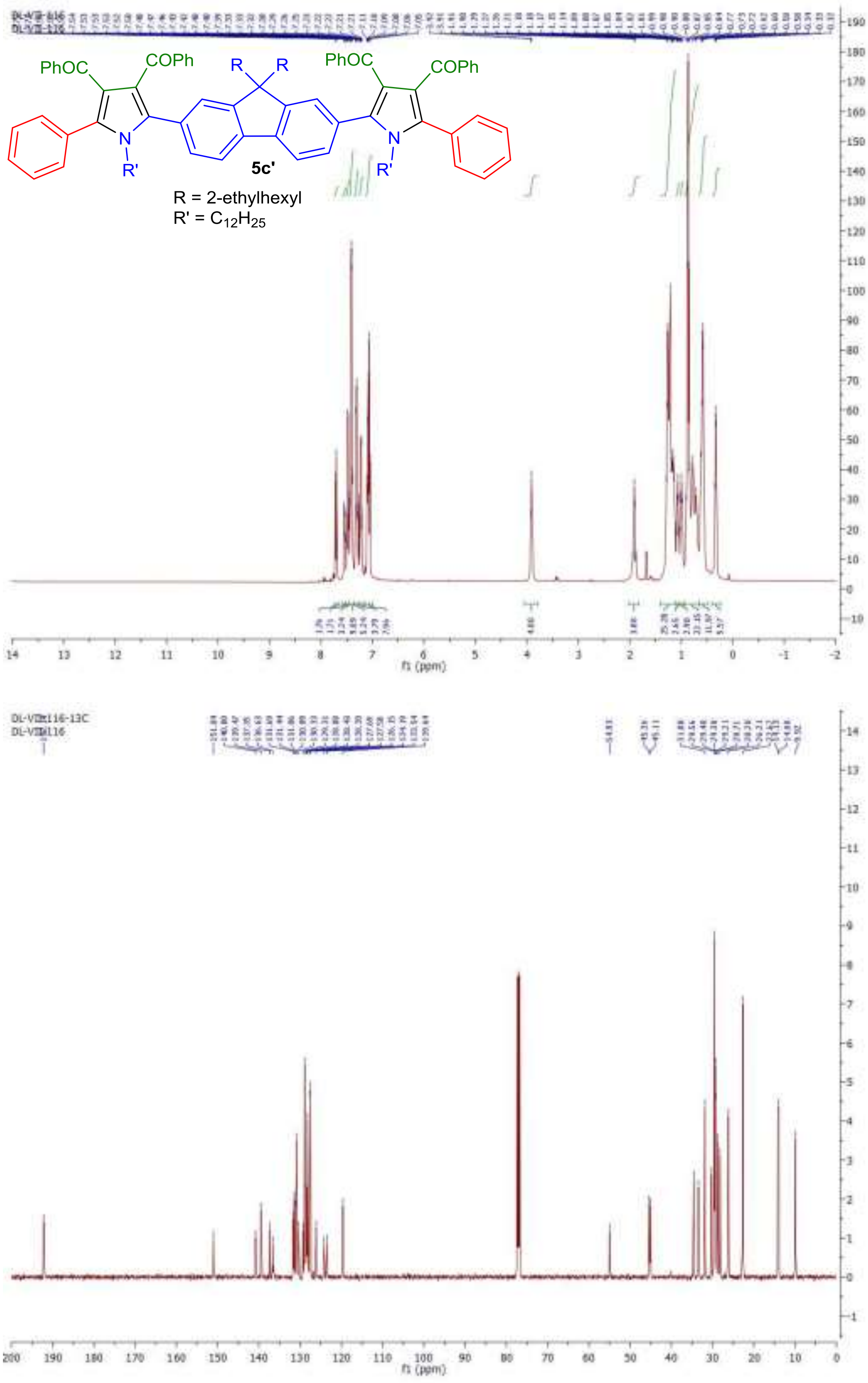

Supplementary Figure 7. ^{1}H and ^{13}C NMR spectra for dimer **5c'**.

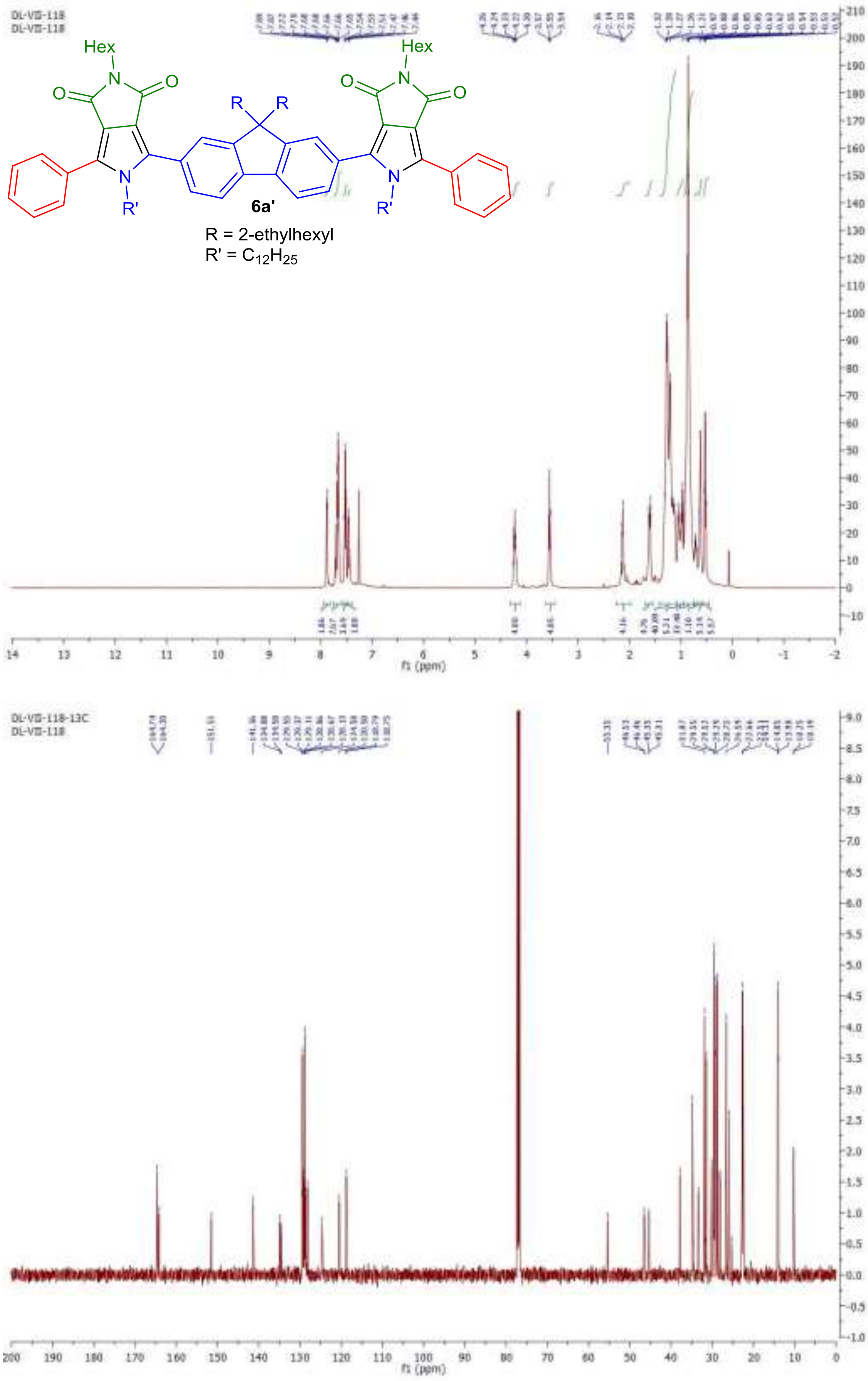

Supplementary Figure 8. ^{1}H and ^{13}C NMR spectra for dimer **6a'**.

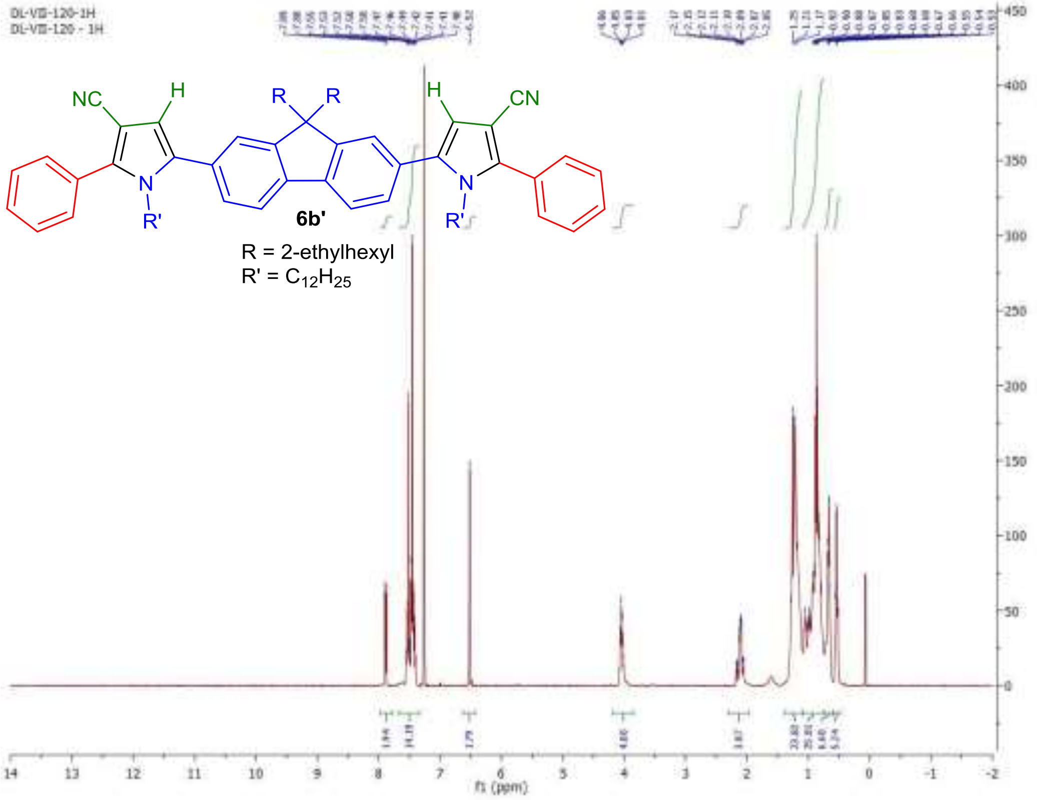

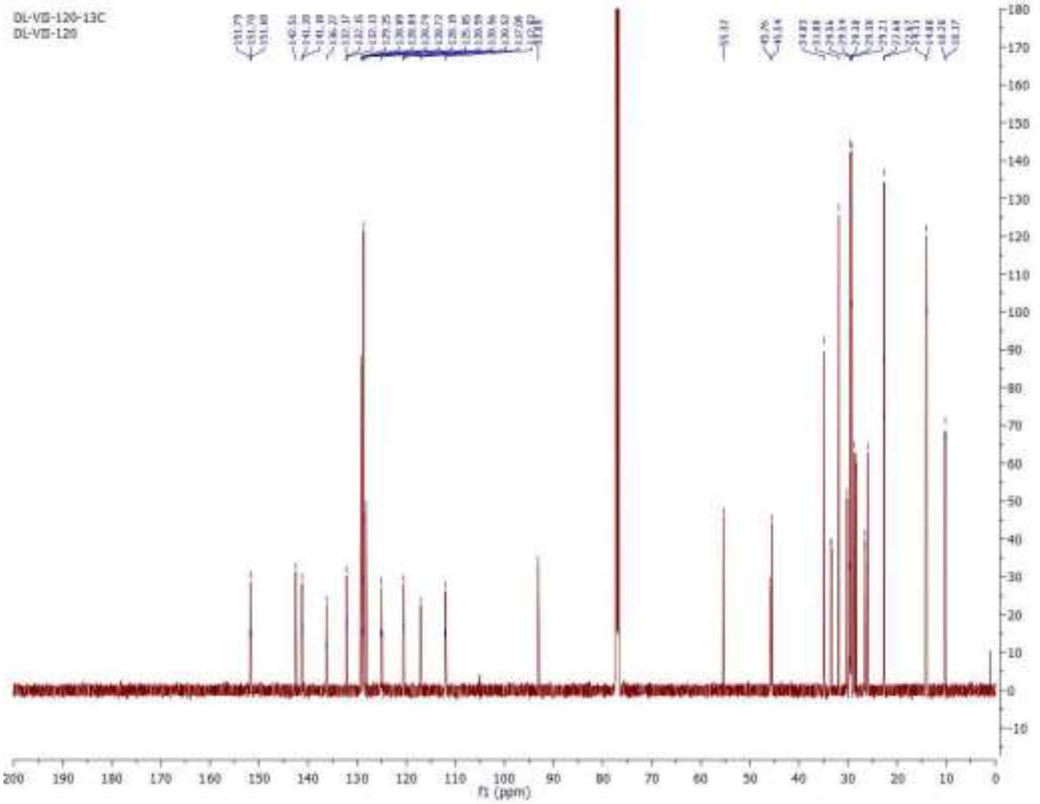

Supplementary Figure 9. ^{1}H and ^{13}C NMR spectra for dimer **6b’**.

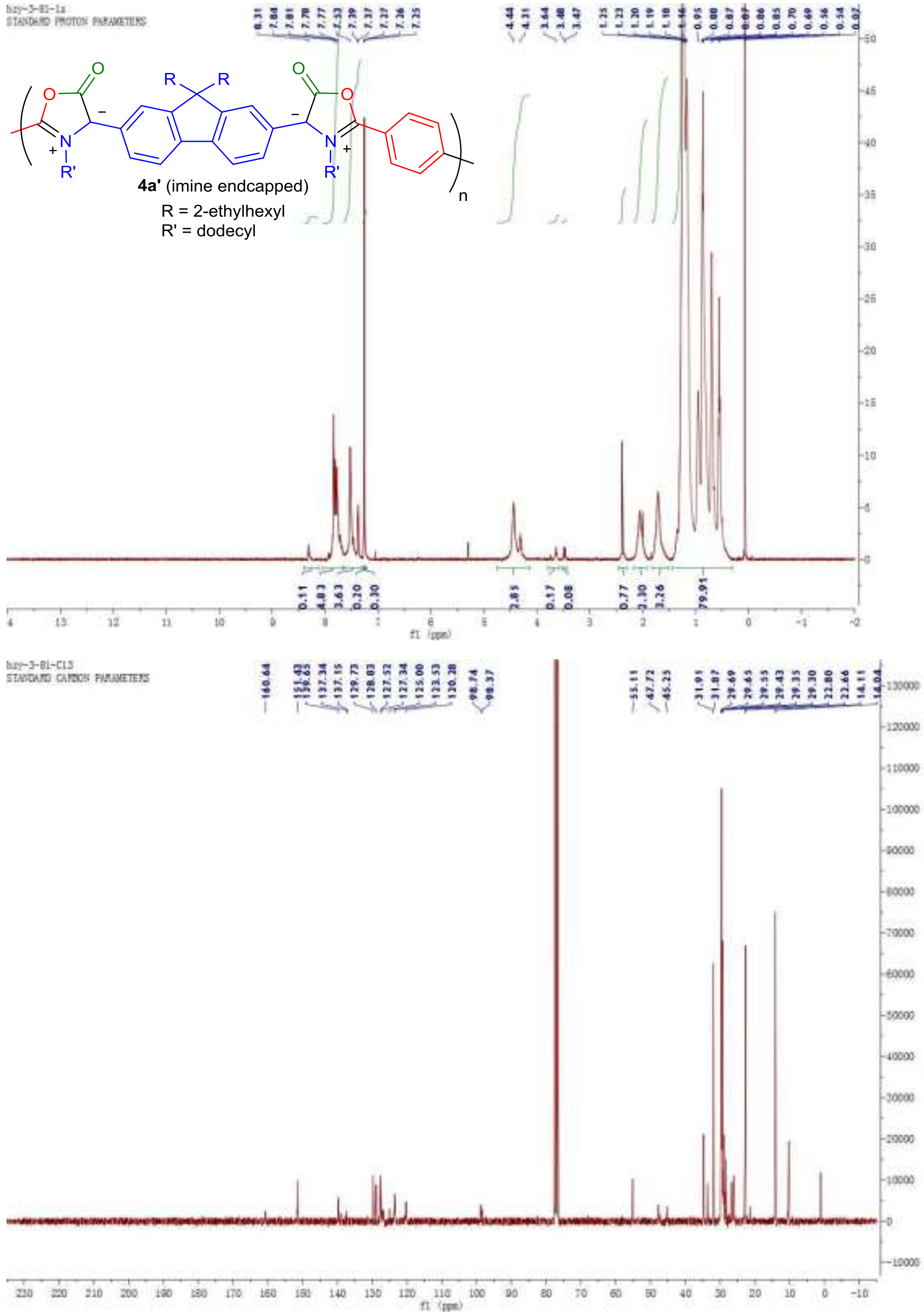

Supplementary Figure 10. ^{1}H and ^{13}C NMR spectra for polymer **4a’** (imine end-capped).

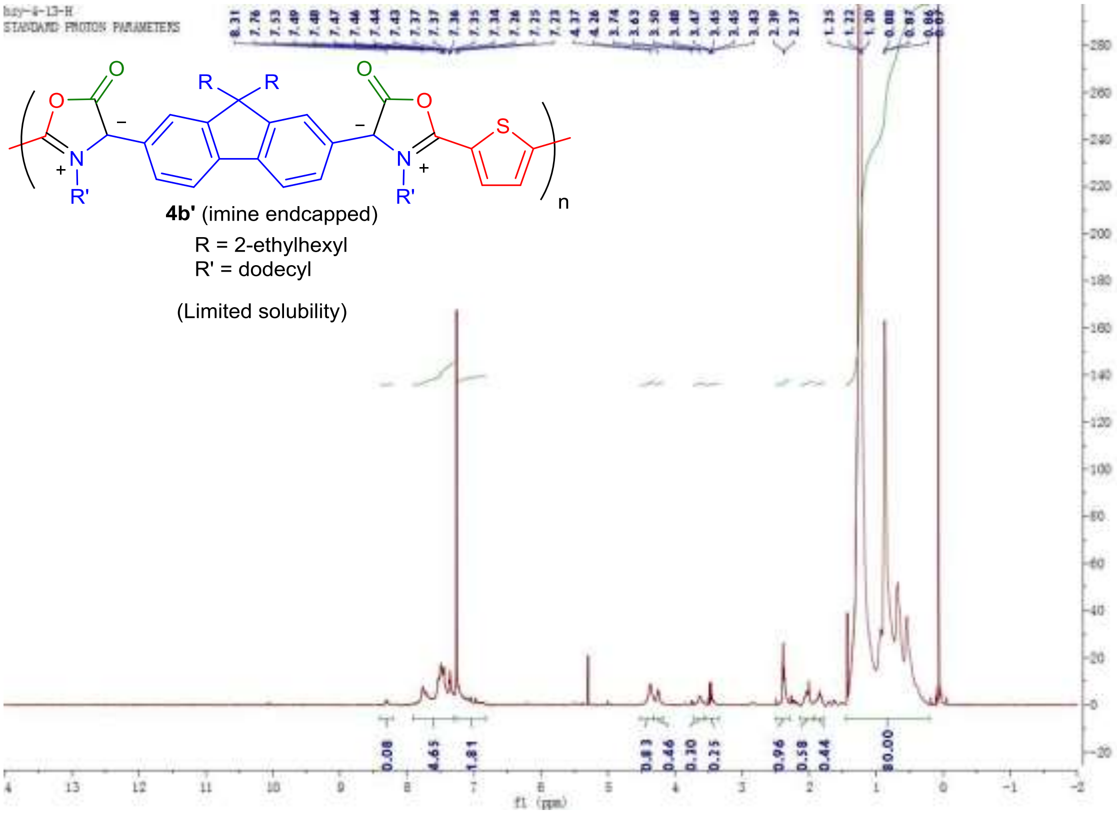

Supplementary Figure 11. ^{1}H NMR spectrum for polymer **4b'** (imine end-capped).

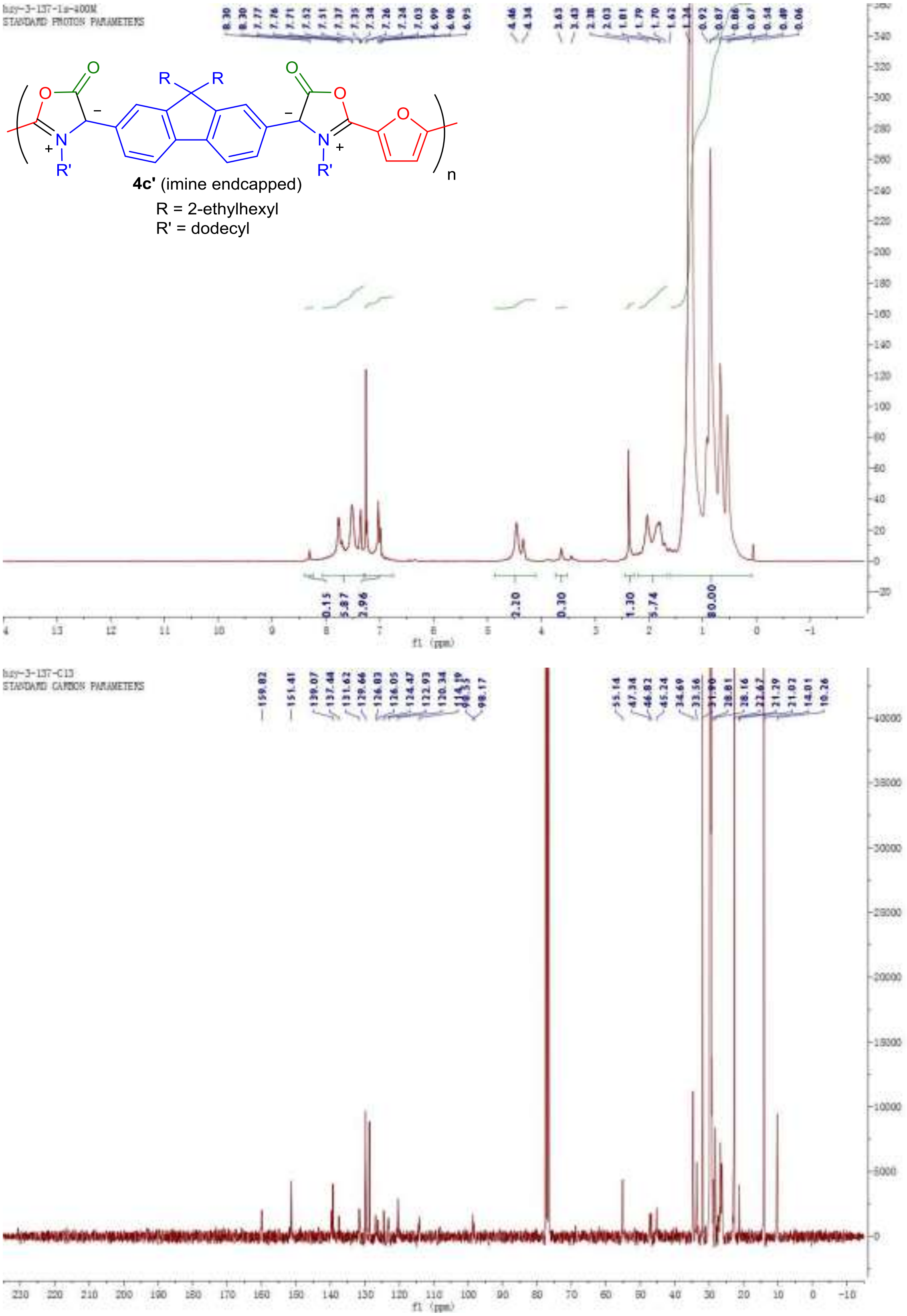

Supplementary Figure 12. ^{1}H and ^{13}C NMR spectra for polymer **4c'** (imine end-capped).

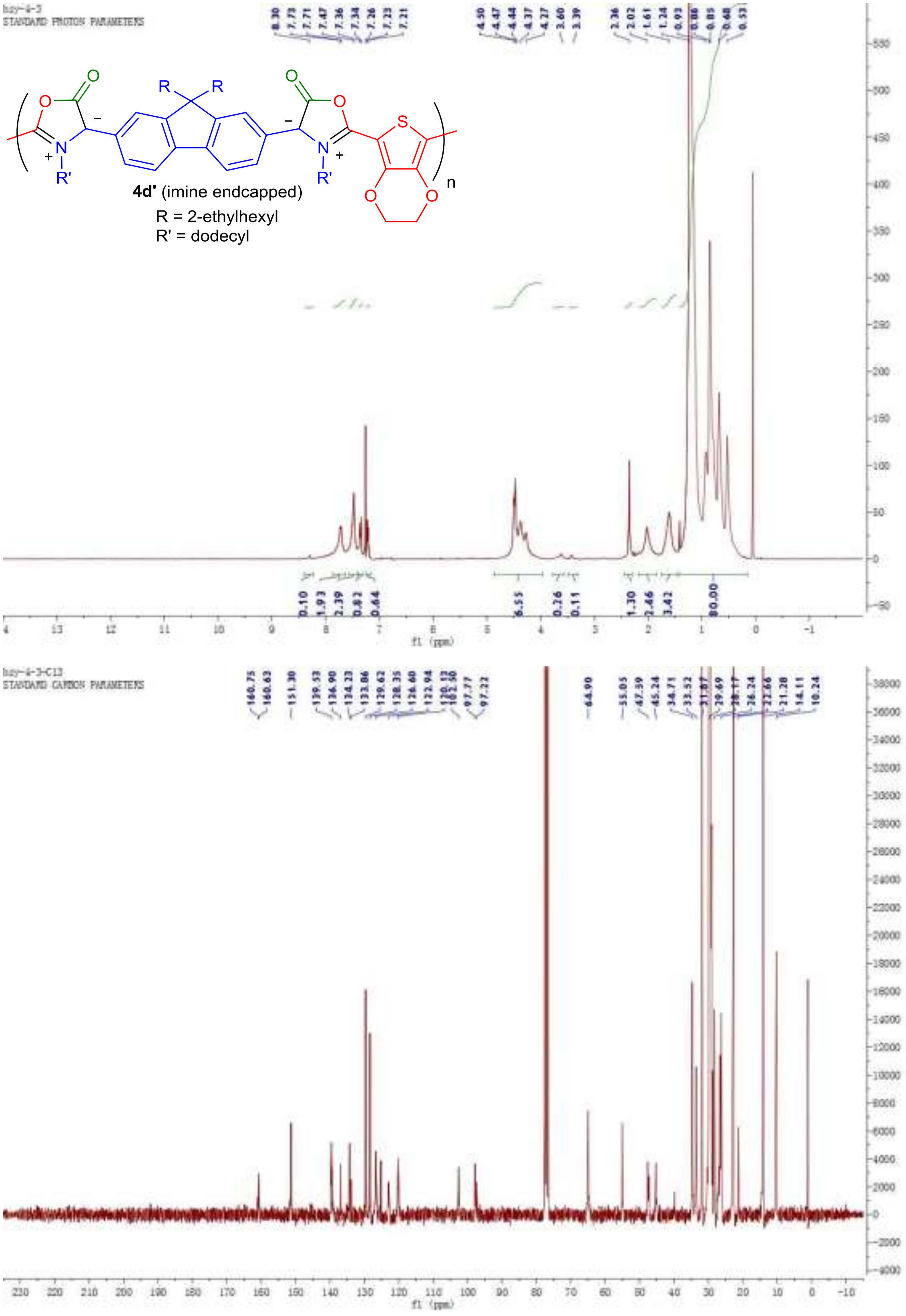

Supplementary Figure 13. ^{1}H and ^{13}C NMR spectra for polymer **4d'** (imine end-capped).

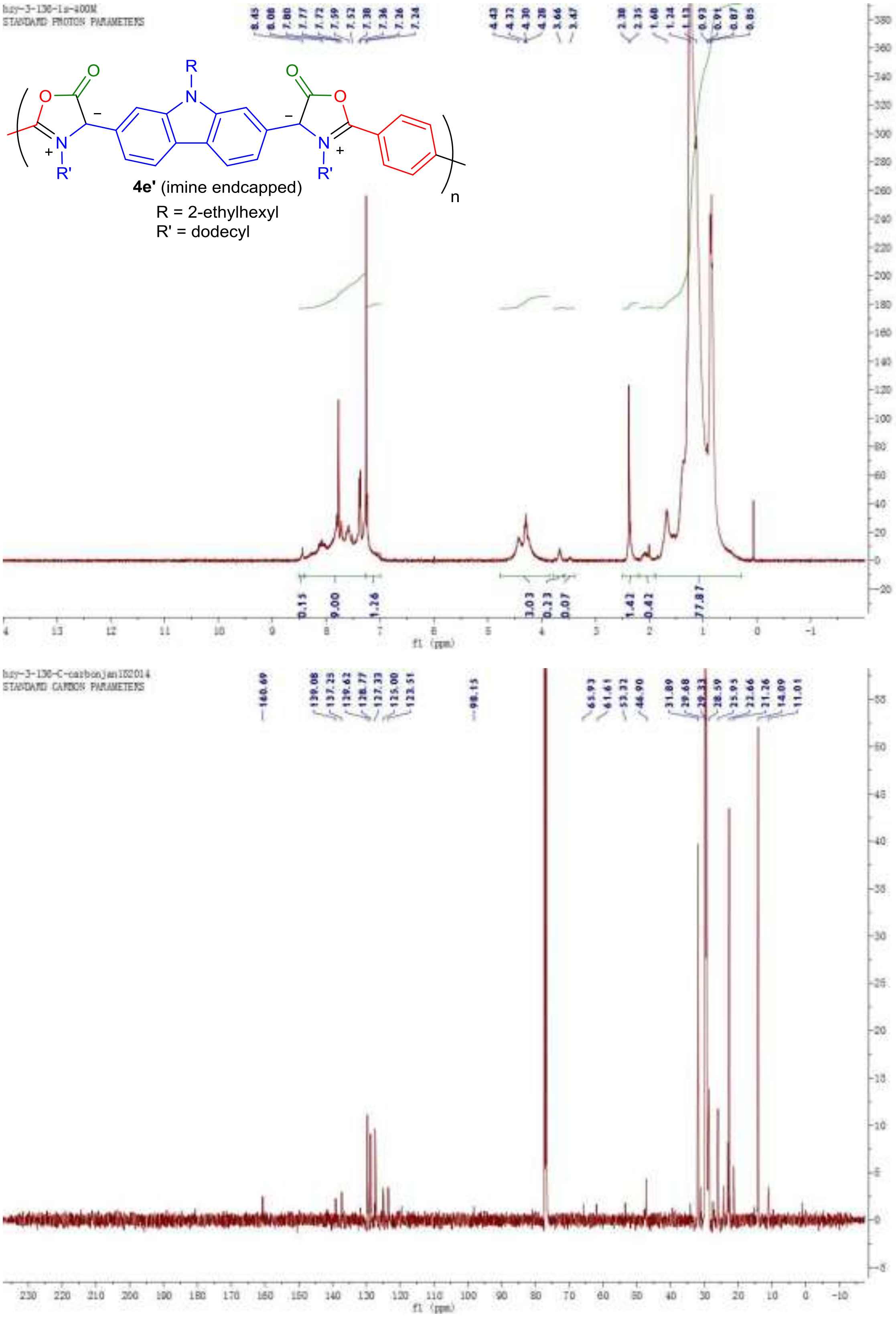

Supplementary Figure 14. ^{1}H and ^{13}C NMR spectra for polymer **4e'** (imine end-capped).

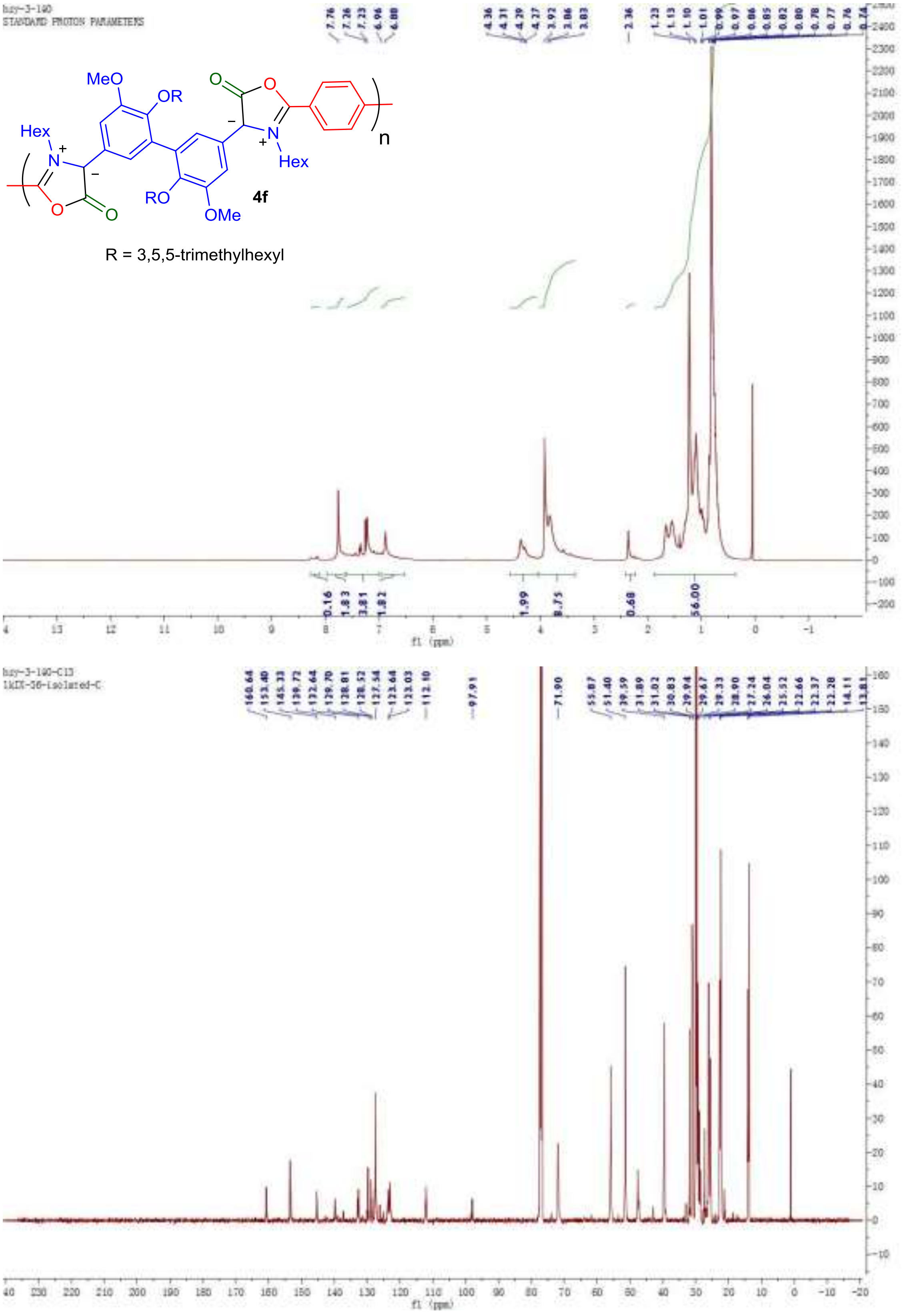

Supplementary Figure 15. ^{1}H and ^{13}C NMR spectra for polymer **4f**.

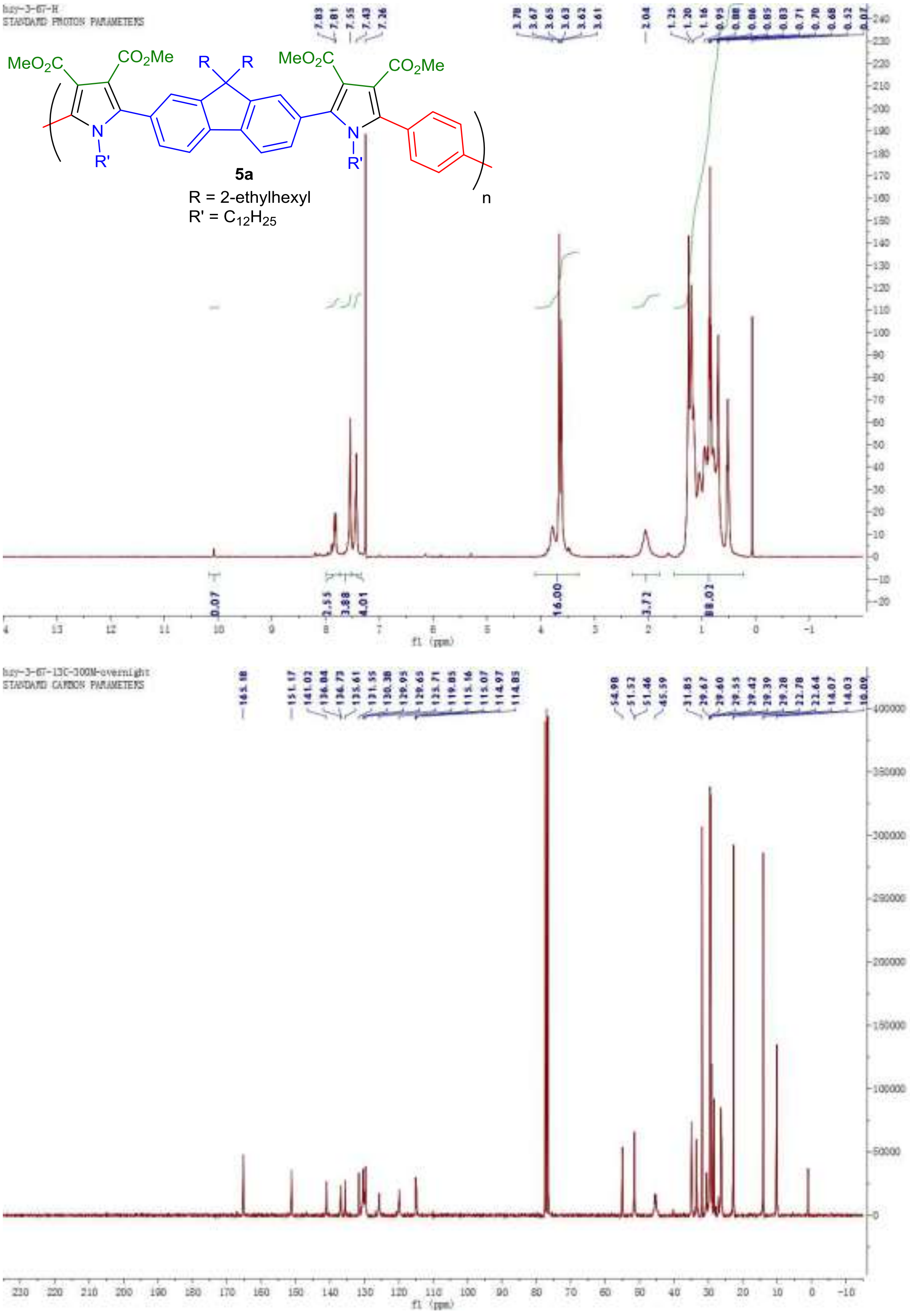

Supplementary Figure 16. ^{1}H and ^{13}C NMR spectra for polymer **5a**.

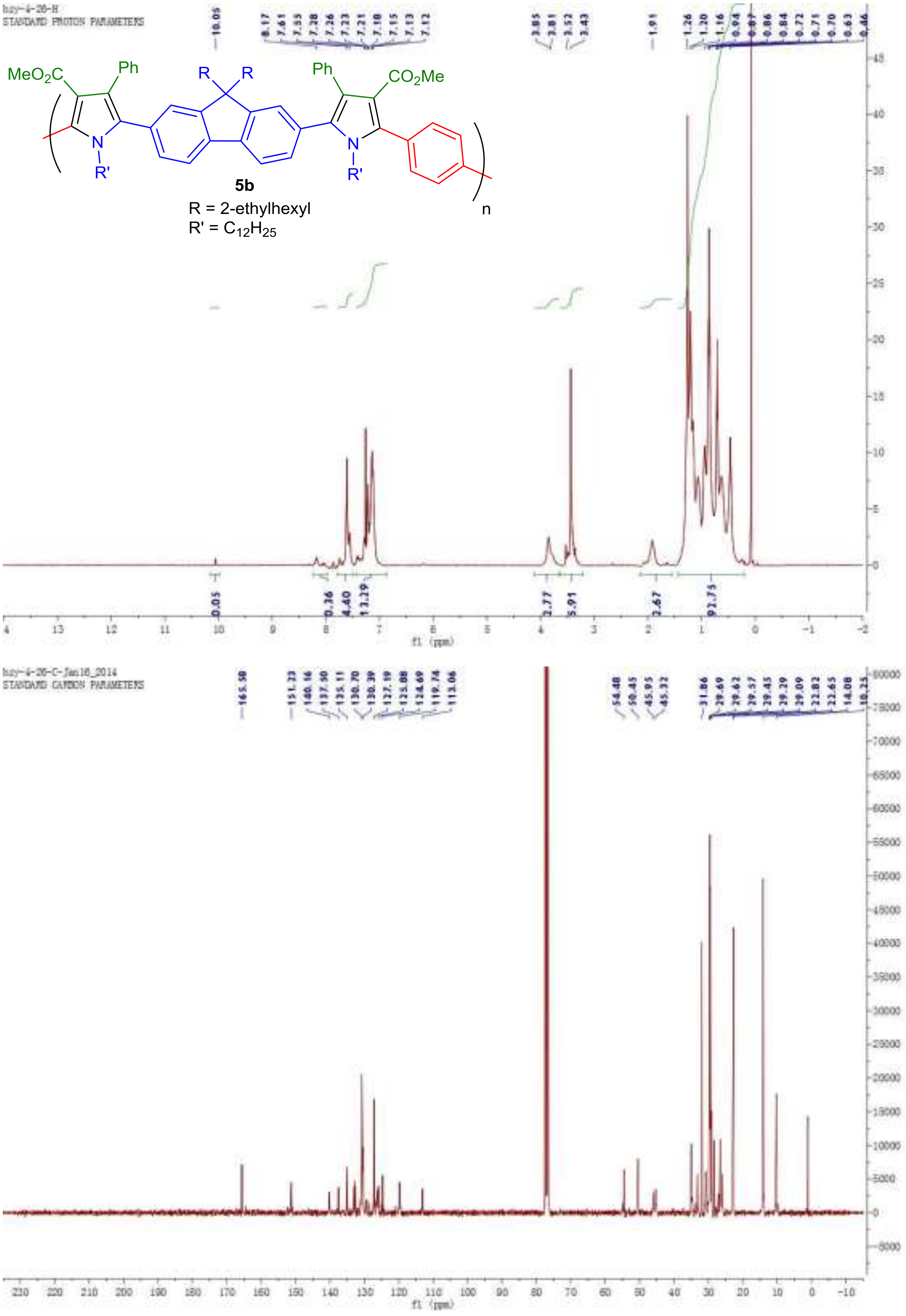

Supplementary Figure 17. ^{1}H and ^{13}C NMR spectra for polymer **5b**.

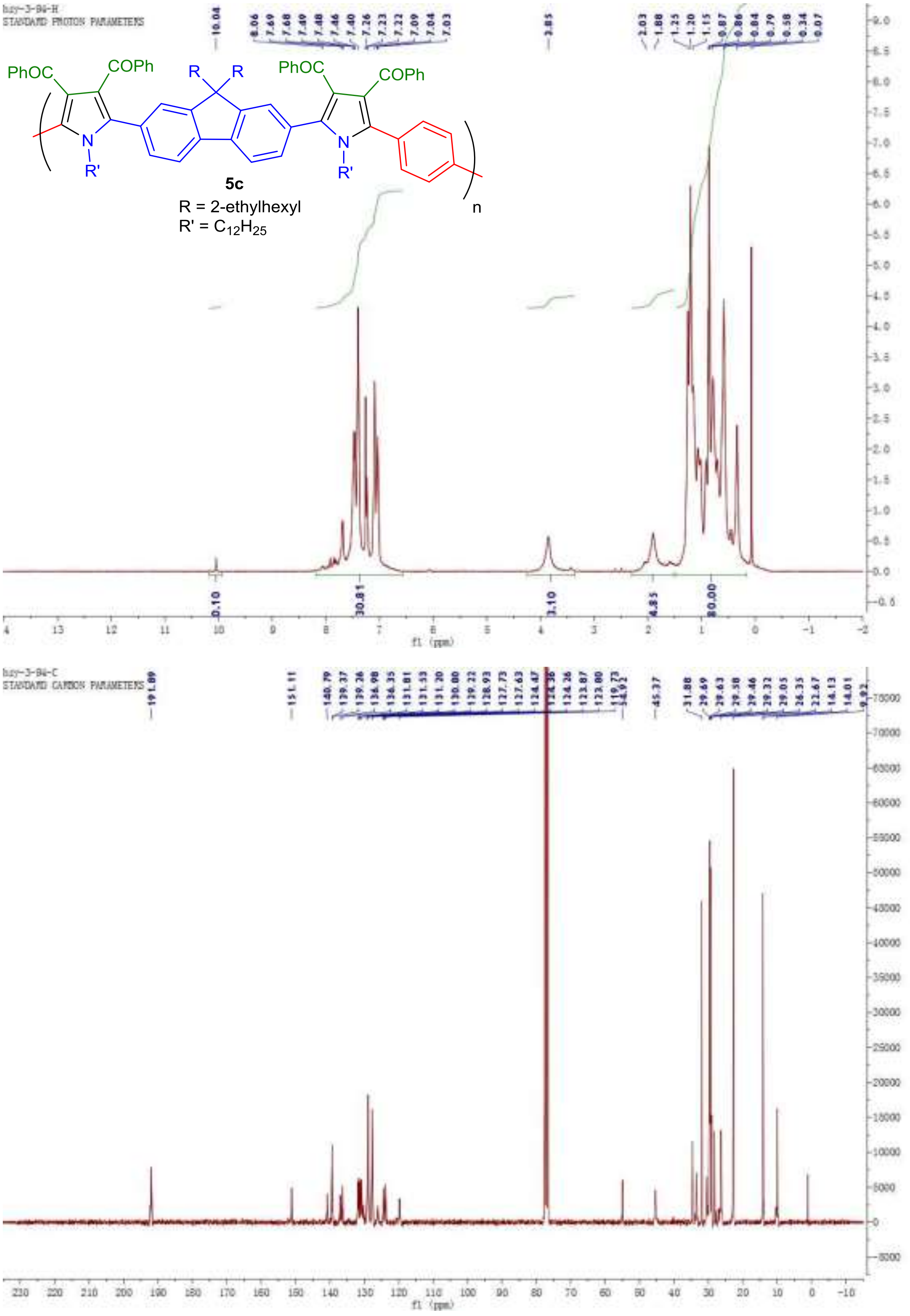

Supplementary Figure 18. ^{1}H and ^{13}C NMR spectra for polymer **5c**.

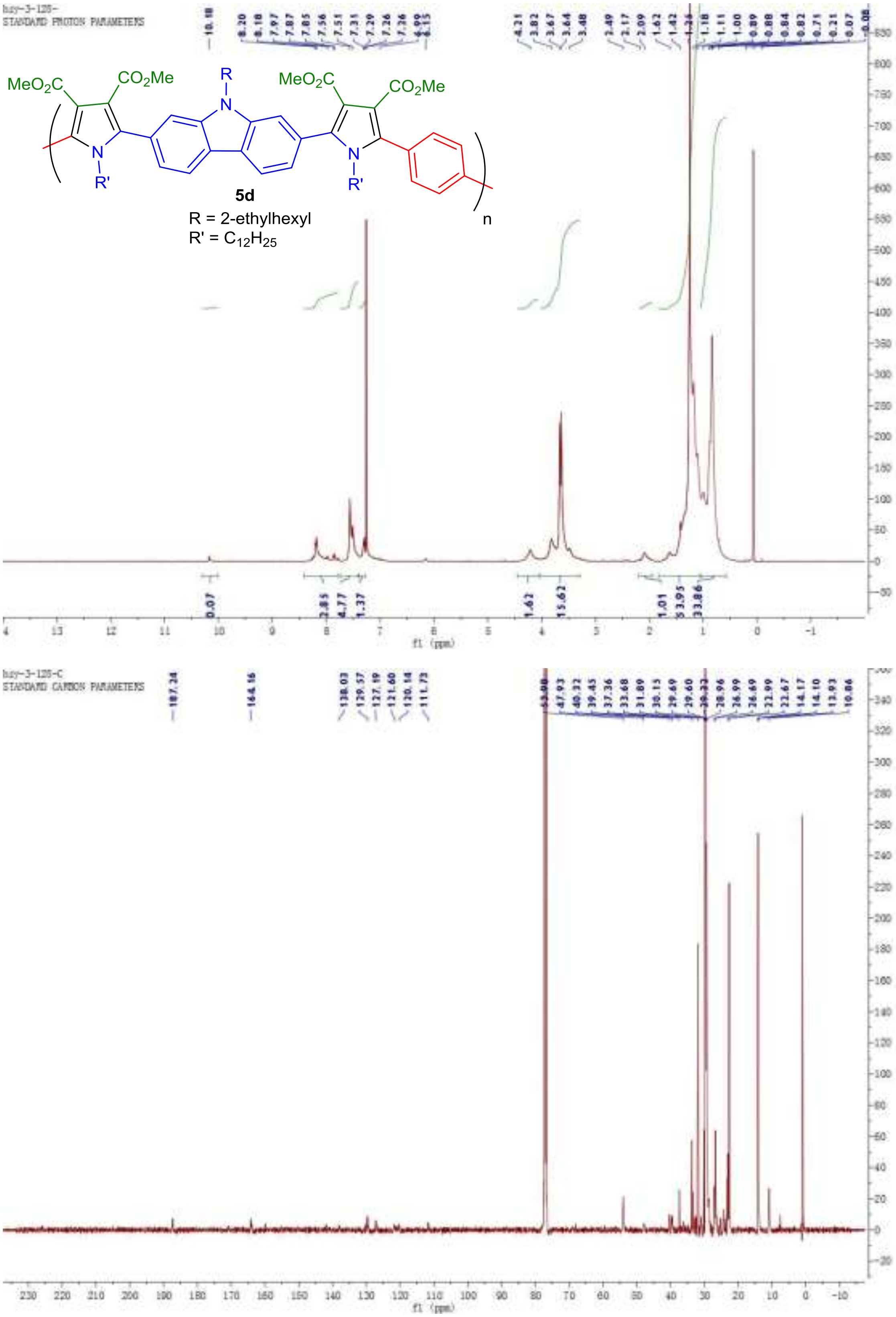

Supplementary Figure 19. ^{1}H and ^{13}C NMR spectra for polymer **5d**.

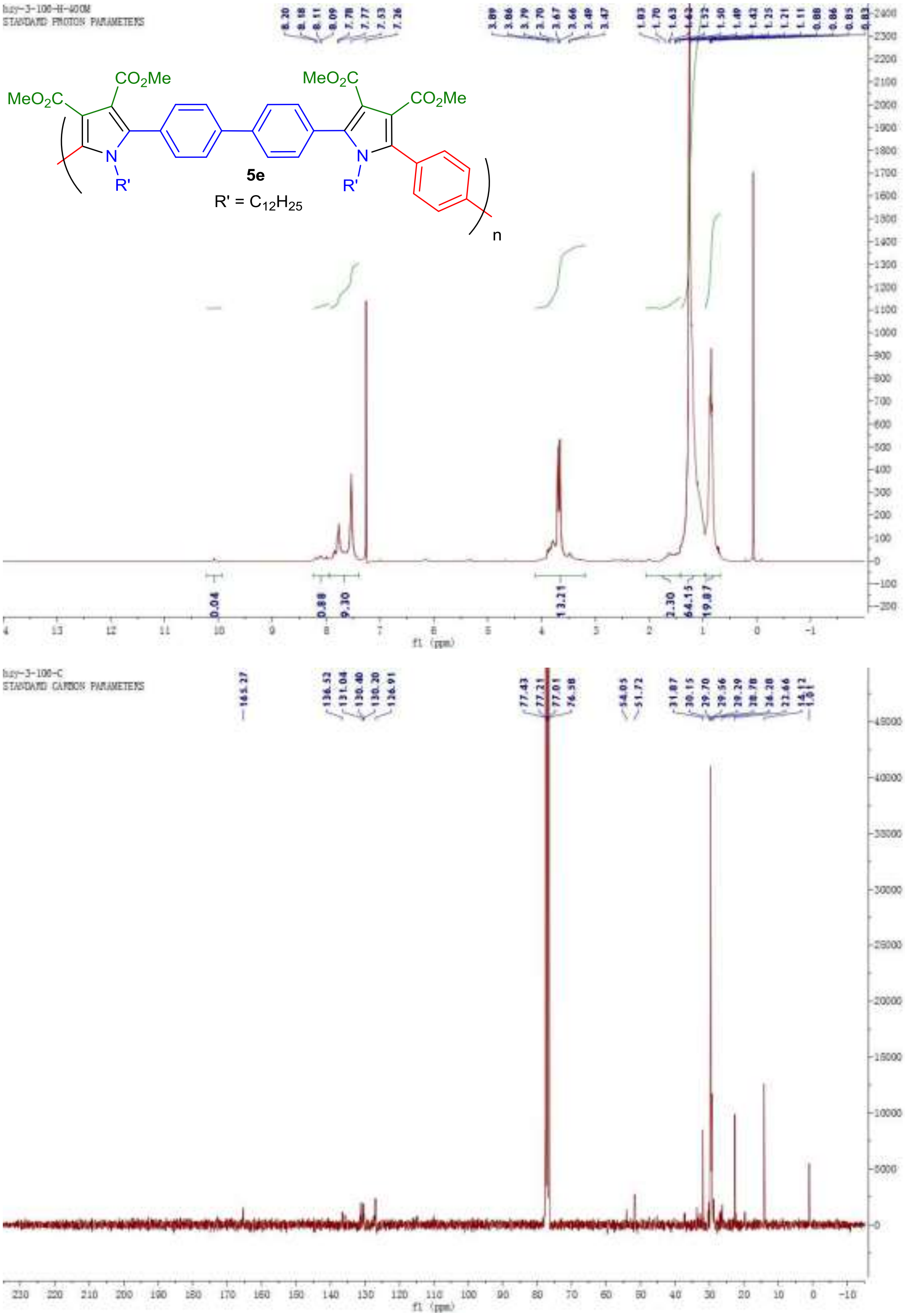

Supplementary Figure 20. ^{1}H and ^{13}C NMR spectra for polymer **5e**.

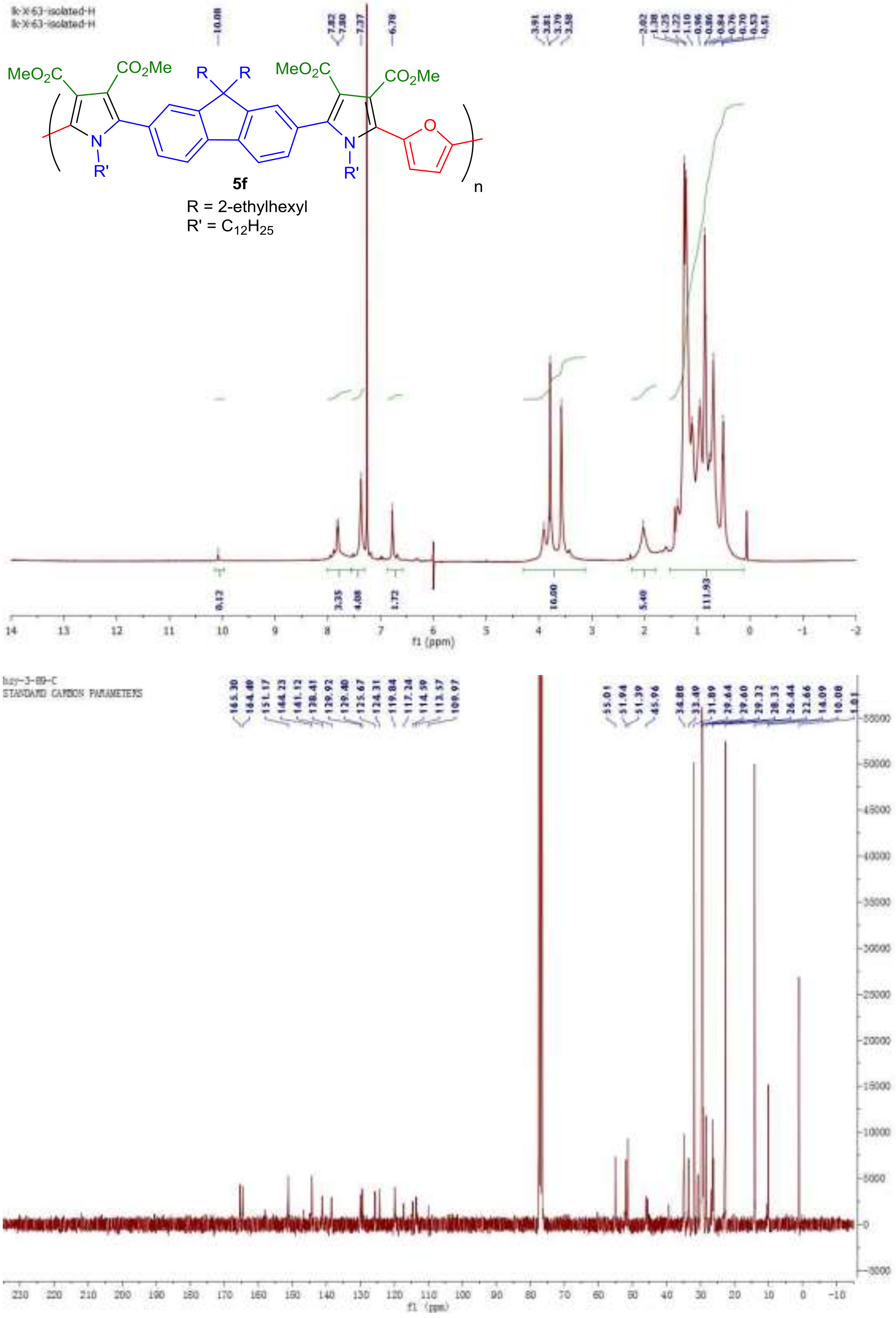

Supplementary Figure 21. ^{1}H and ^{13}C NMR spectra for polymer **5f**.

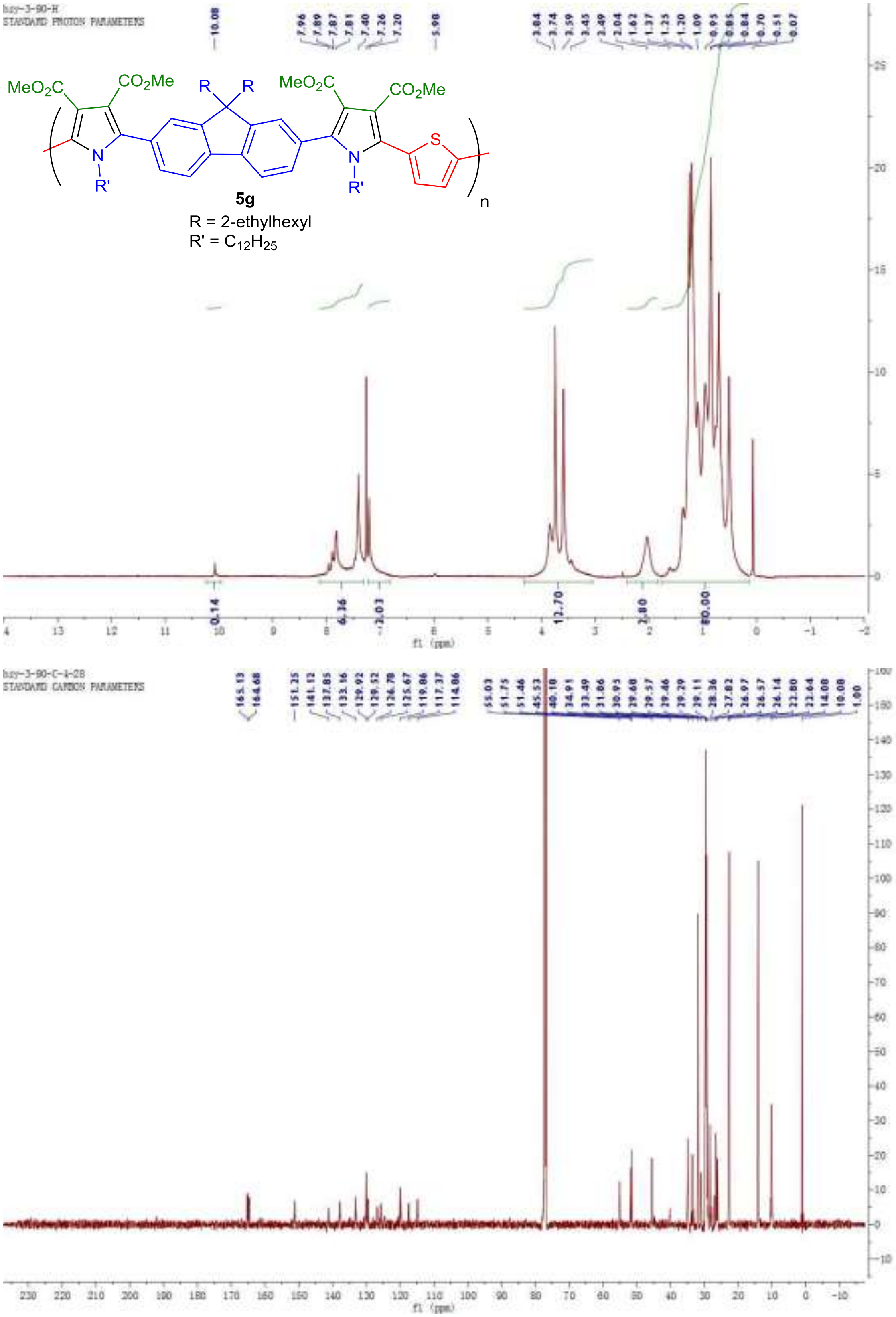

Supplementary Figure 22. ^{1}H and ^{13}C NMR spectra for polymer **5g**.

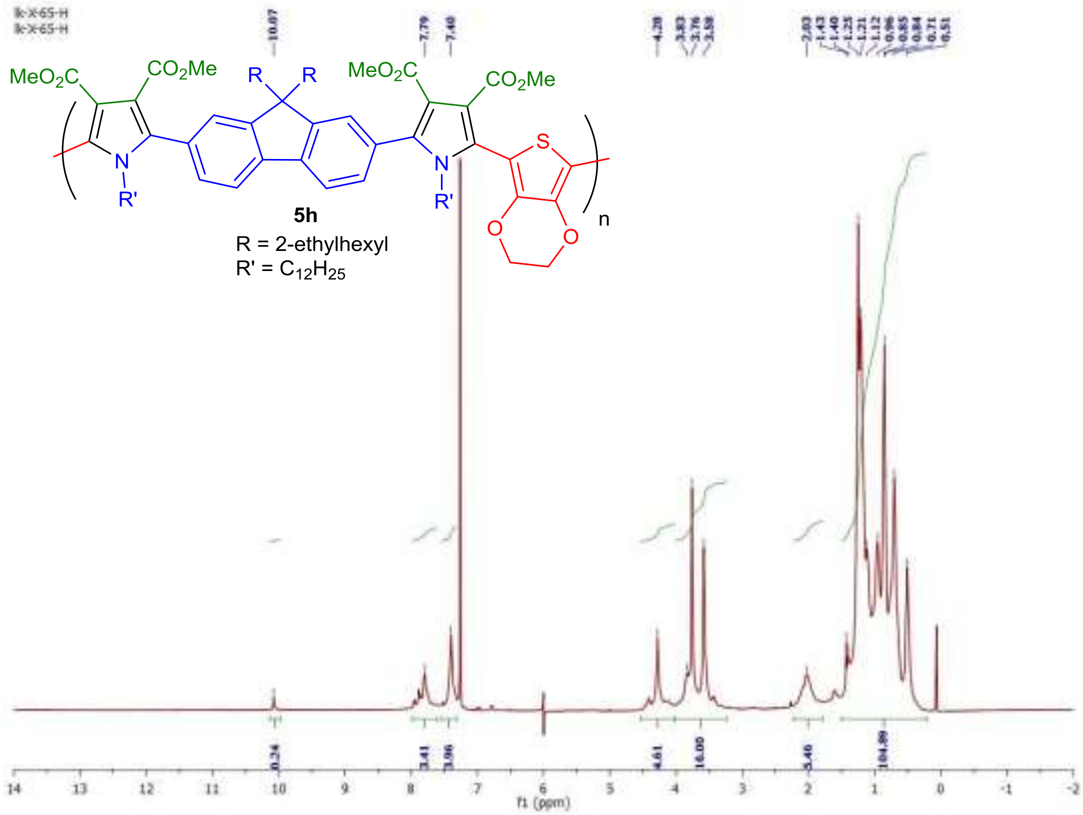

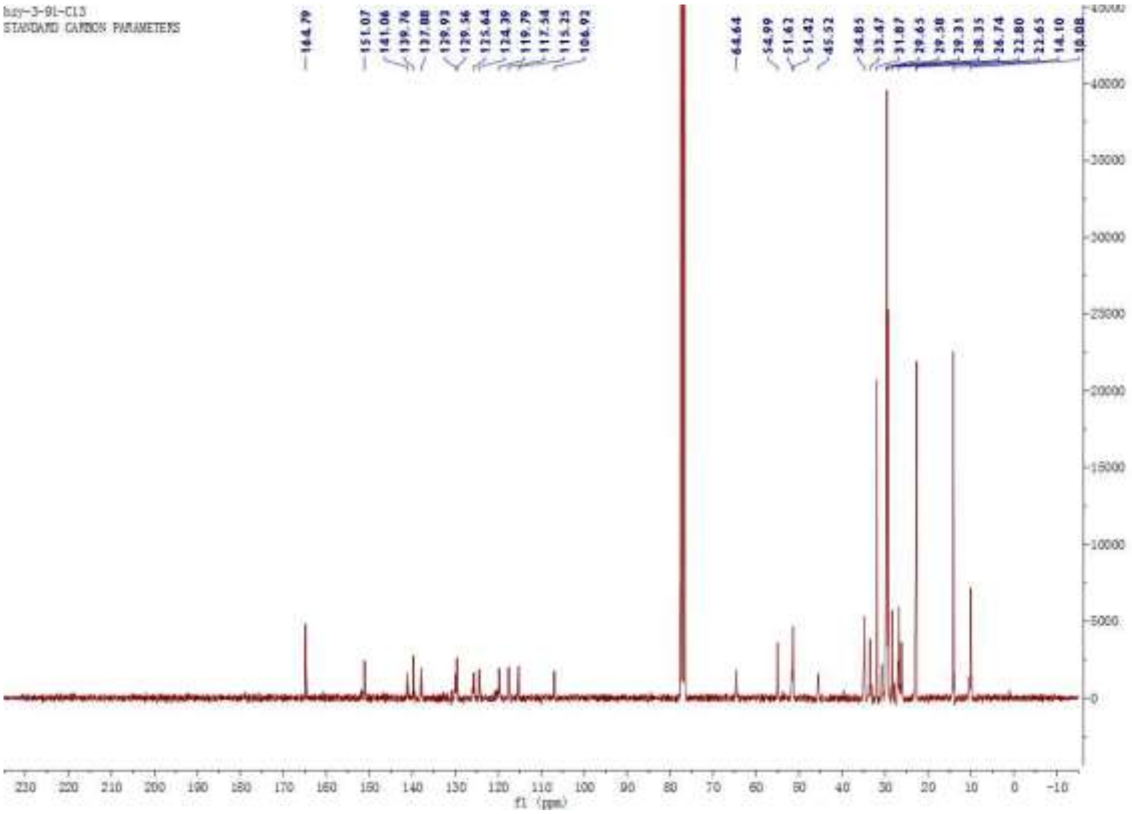

Supplementary Figure 23. ^{1}H and ^{13}C NMR spectra for polymer **5h**.

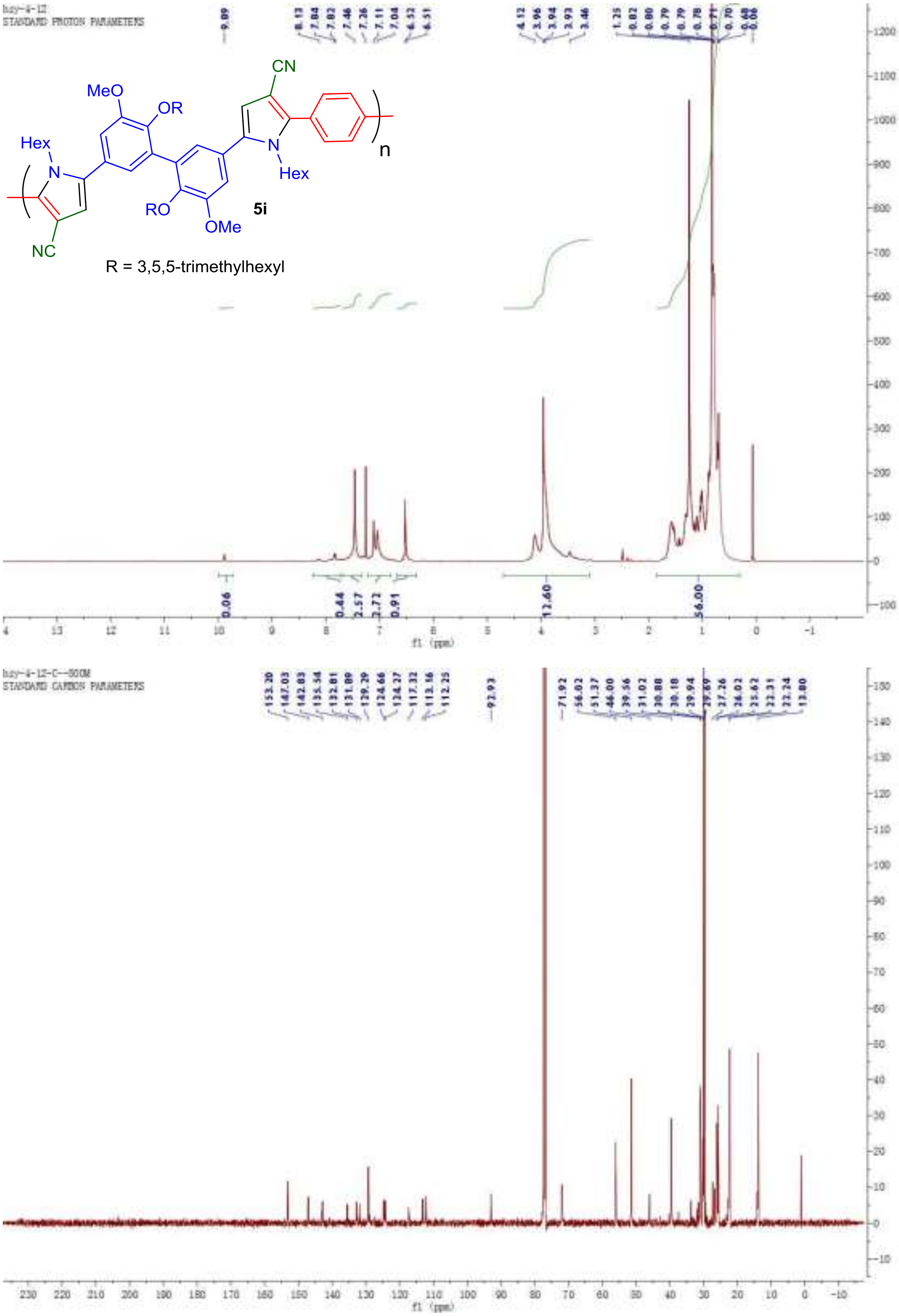

Supplementary Figure 24. ^{1}H and ^{13}C NMR spectra for polymer **5i**.

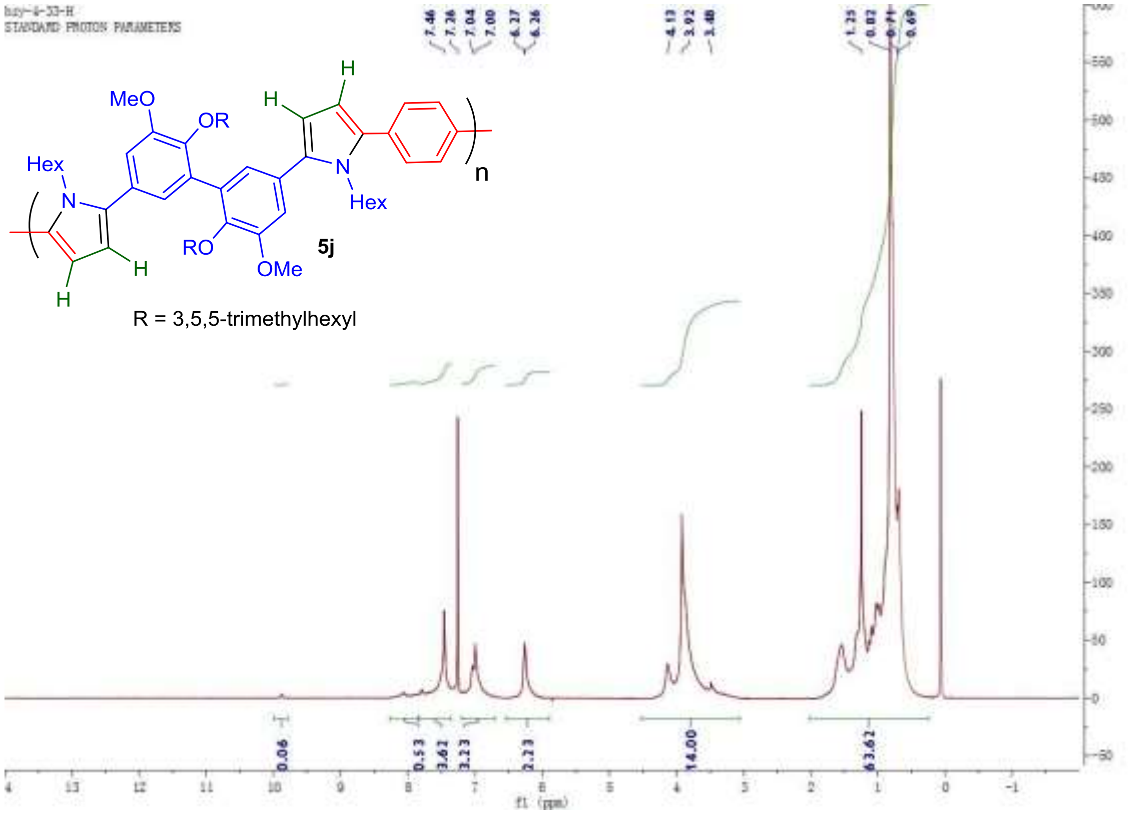

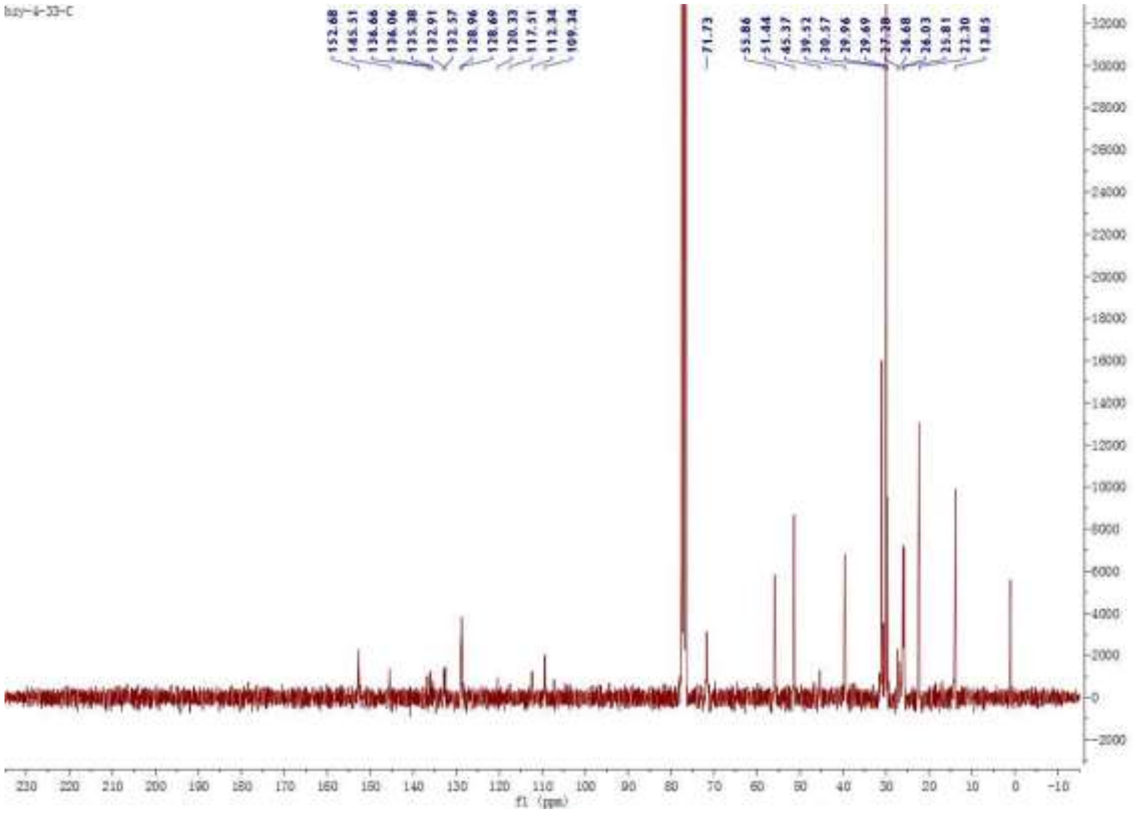

Supplementary Figure 25. ^{1}H and ^{13}C NMR spectra for polymer **5j**.

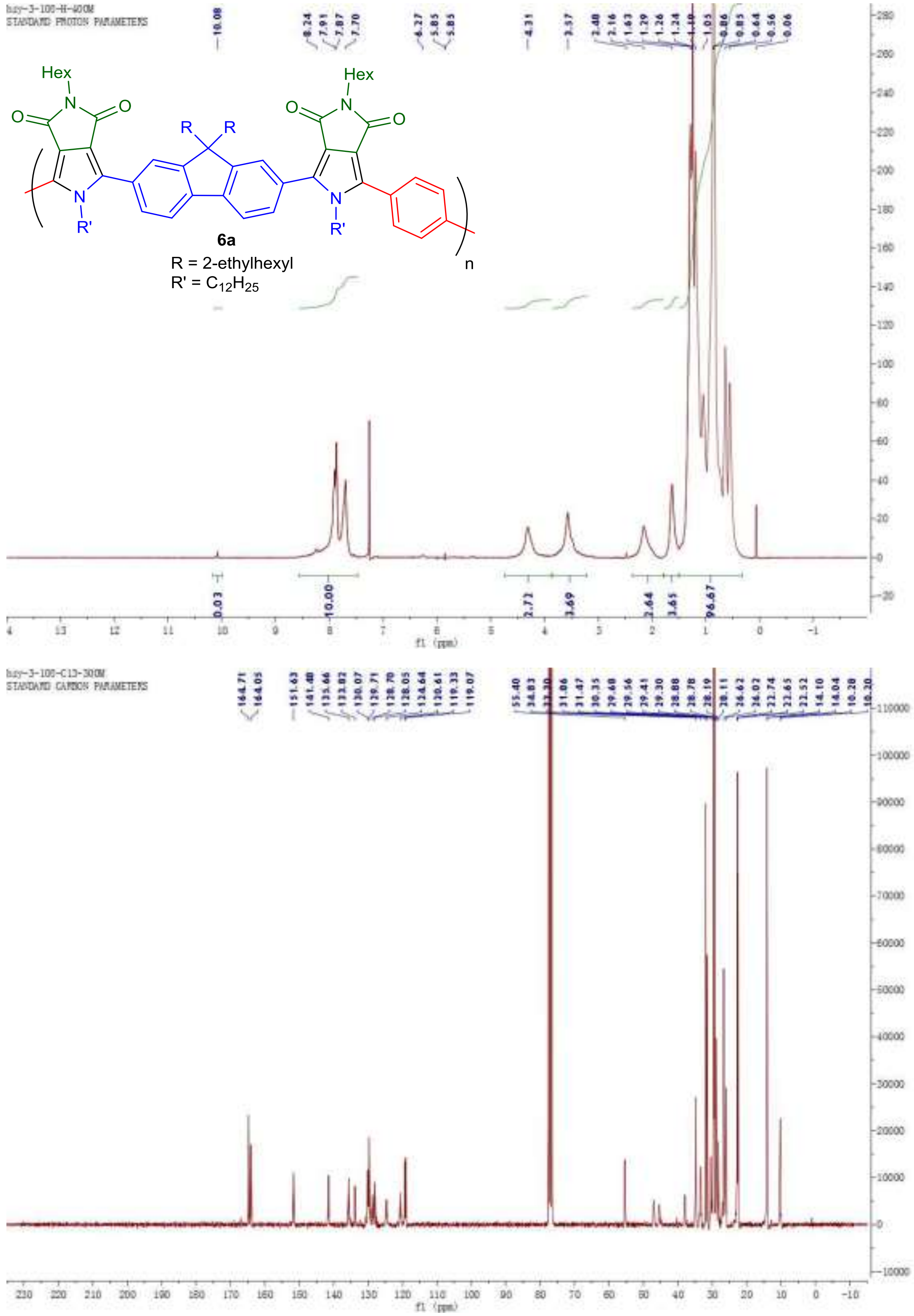

Supplementary Figure 26. ^{1}H and ^{13}C NMR spectra for polymer **6a**.

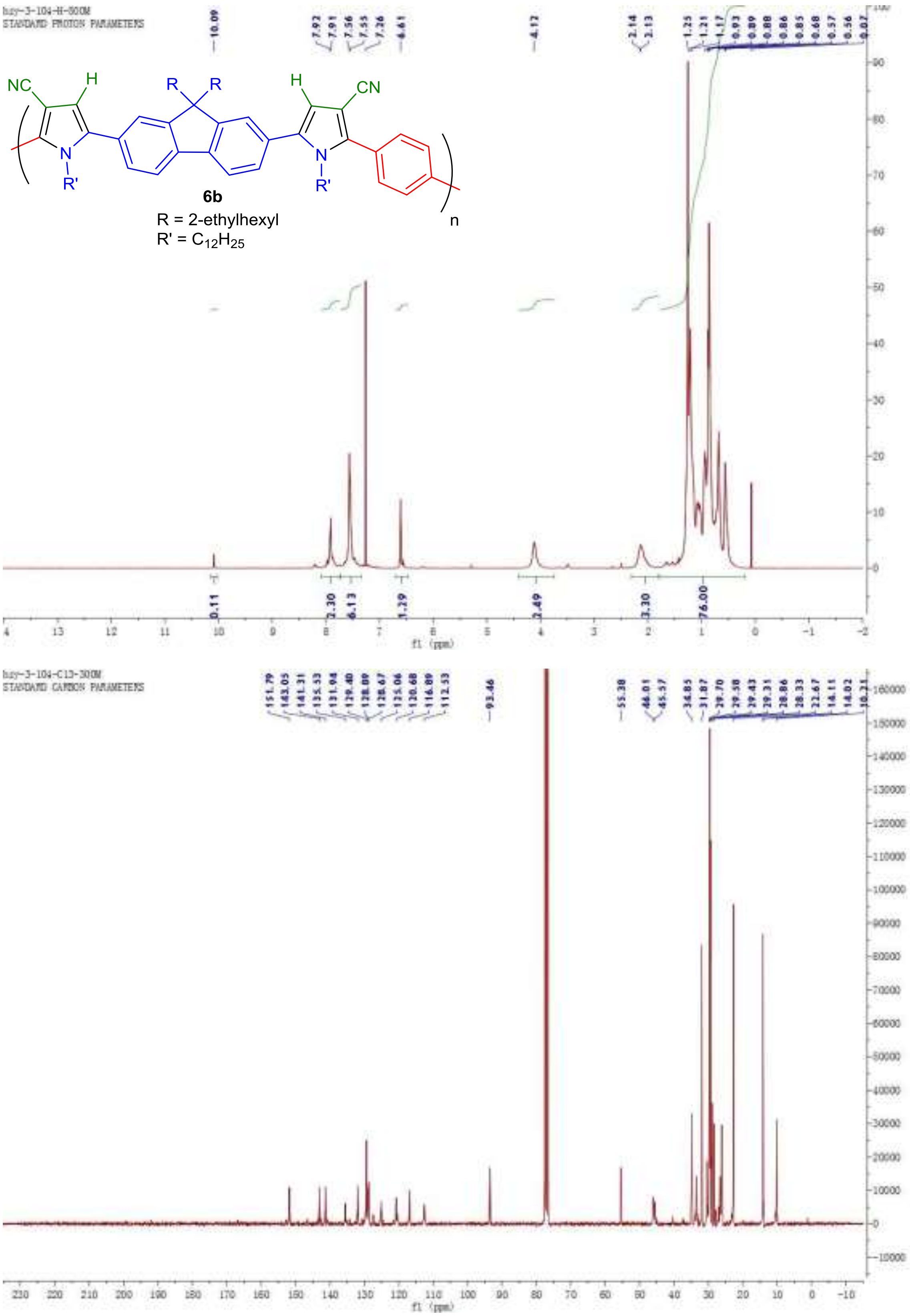

Supplementary Figure 27. ^{1}H and ^{13}C NMR spectra for polymer **6b**.

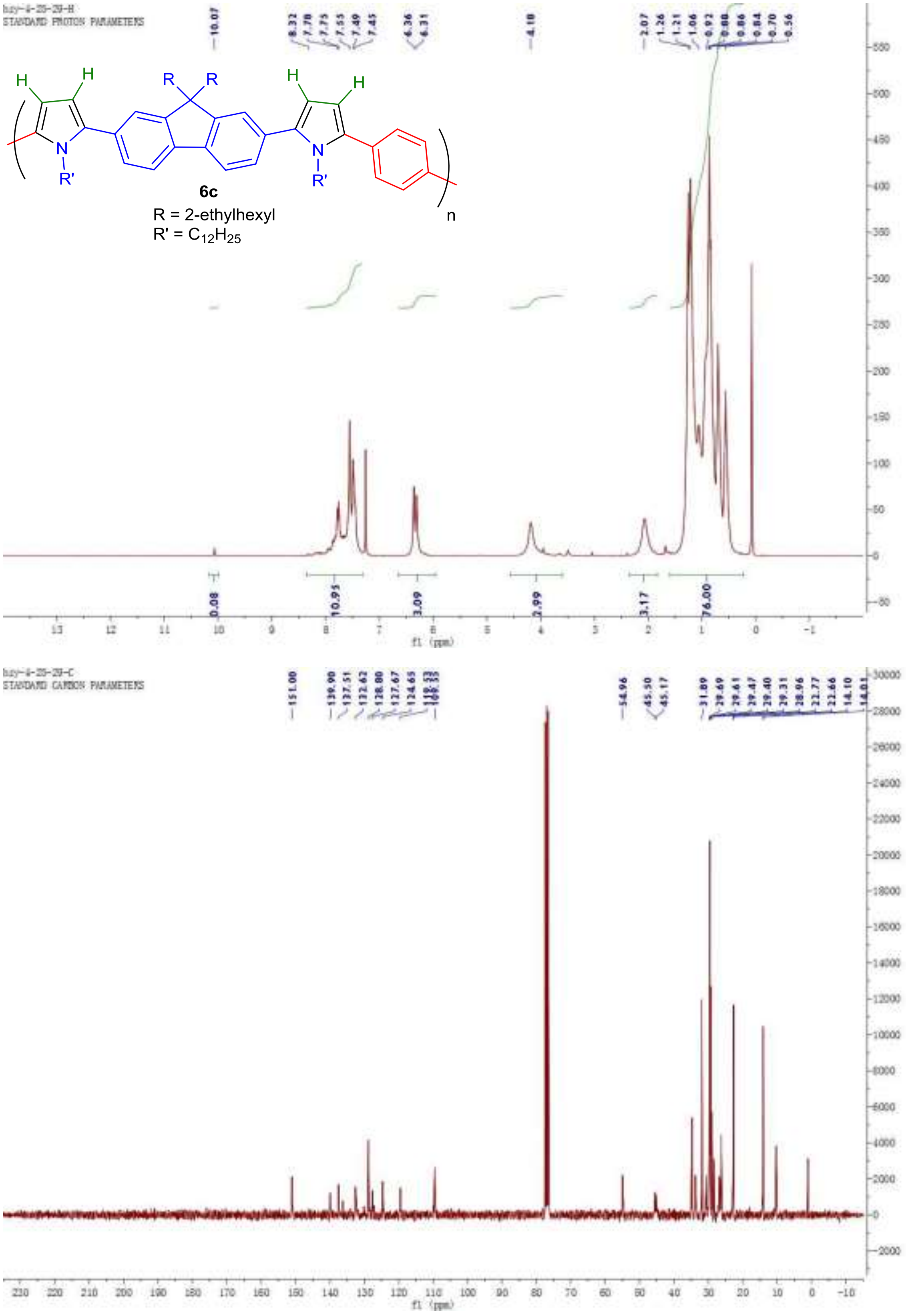

Supplementary Figure 28. ^{1}H and ^{13}C NMR spectra for polymer **6c**.

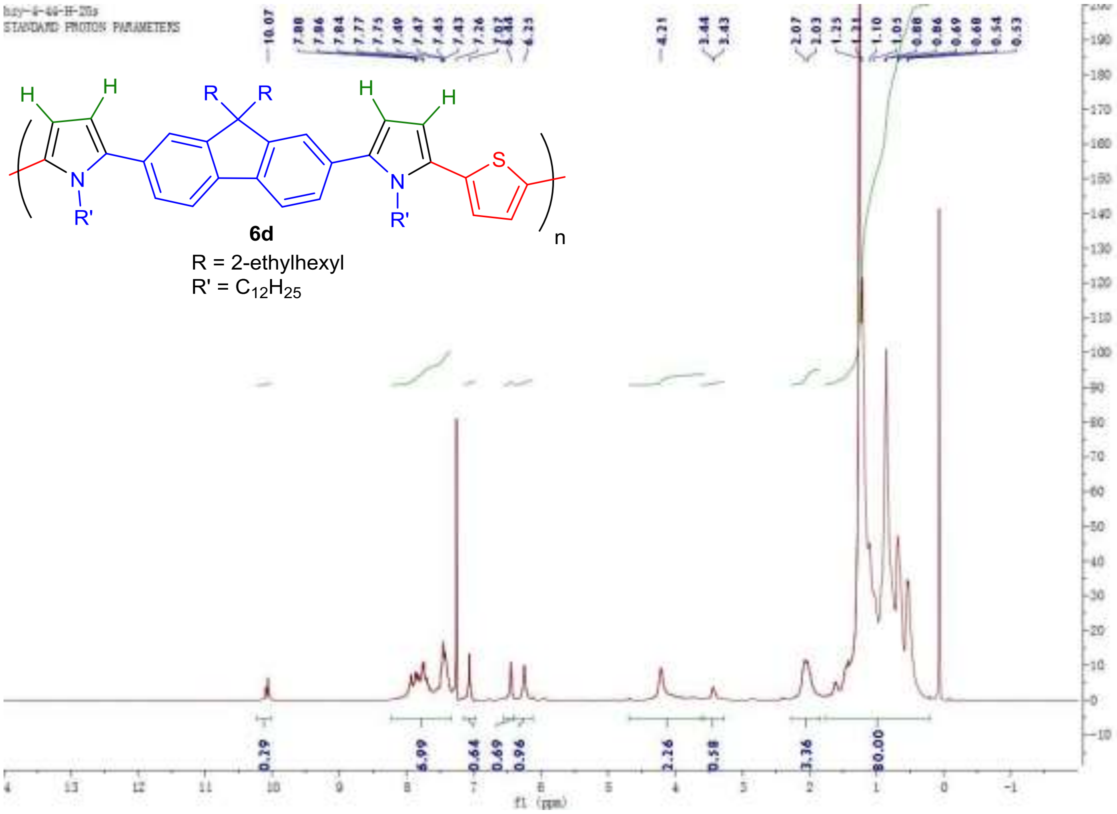

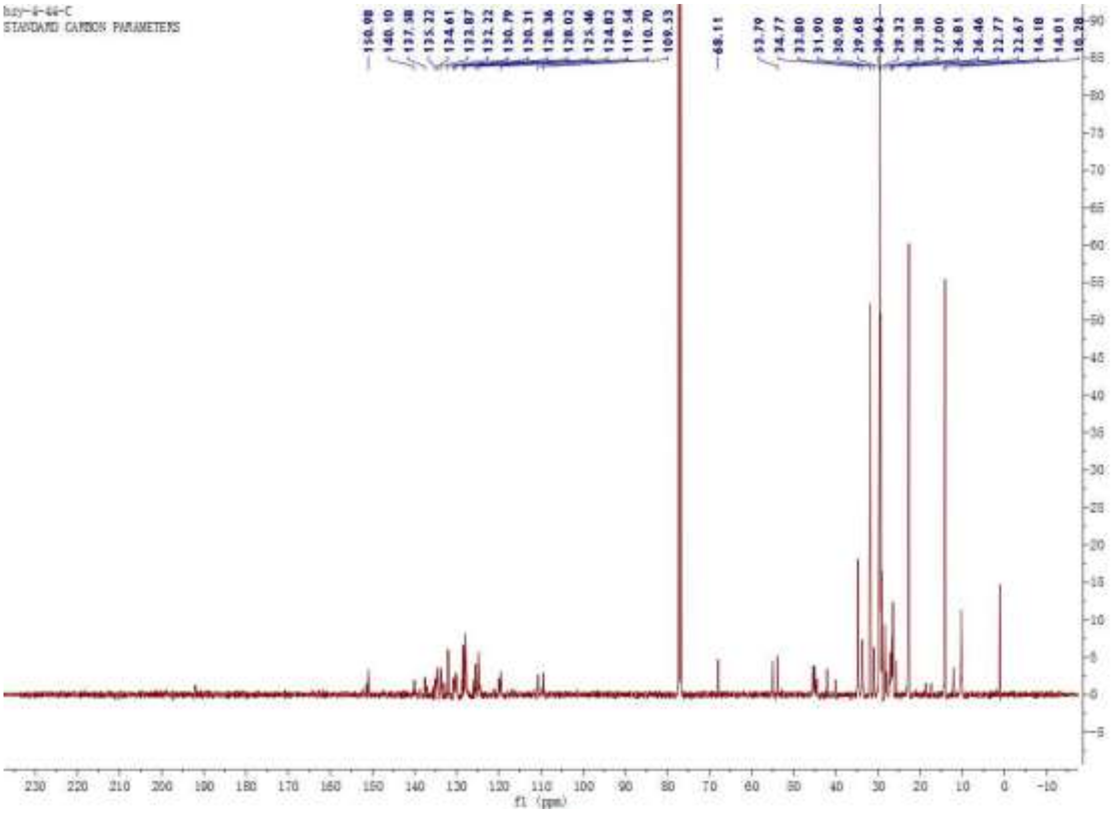

Supplementary Figure 29. ^{1}H and ^{13}C NMR spectra for polymer **6d**.

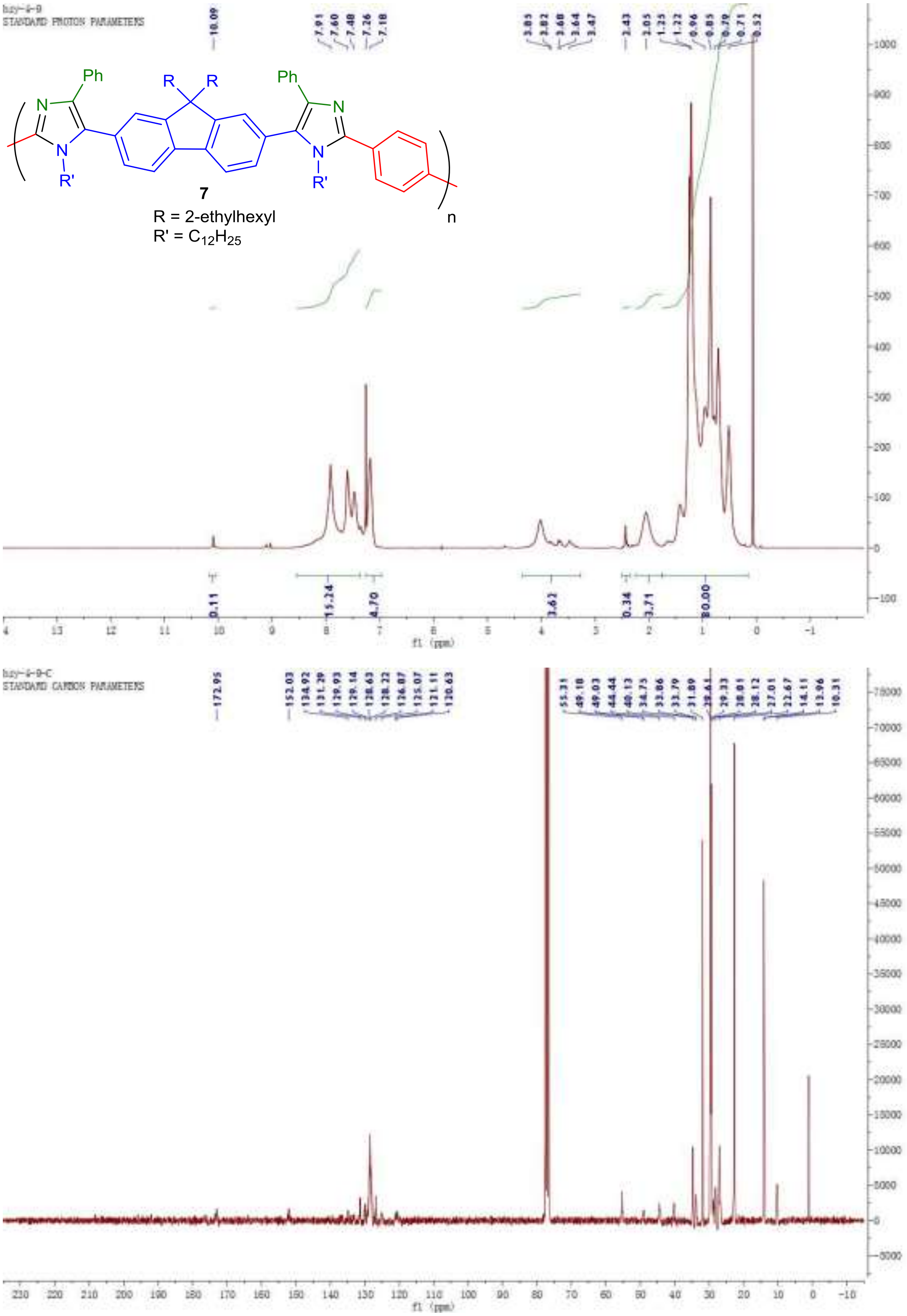

Supplementary Figure 30. ^{1}H and ^{13}C NMR spectra for polymer **7**.

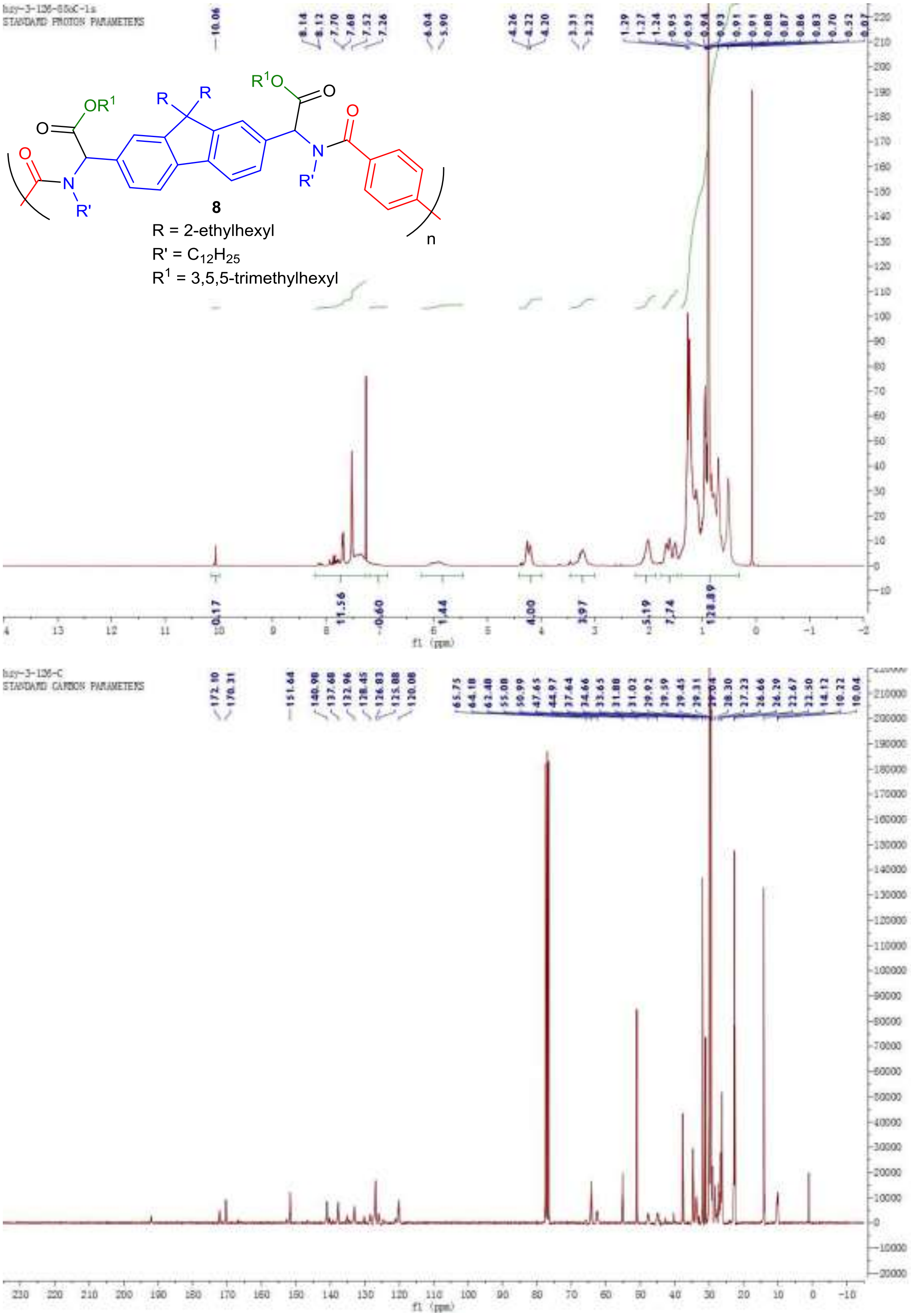

Supplementary Figure 31. ^{1}H and ^{13}C NMR spectra for polymer **8**.

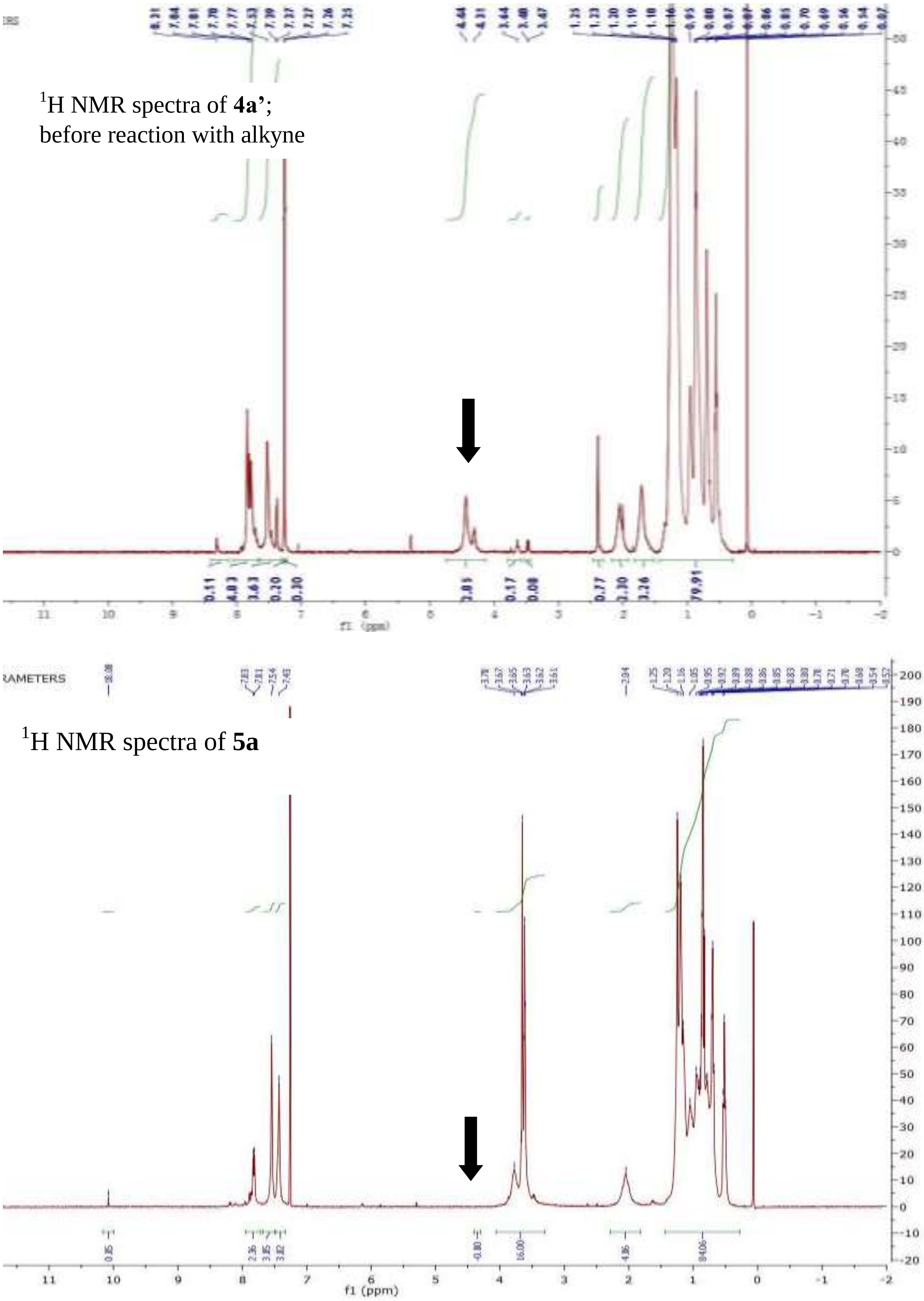

Supplementary Figure 32. ^{1}H NMR spectra of **4a'** before reaction with the alkyne and after to form **5a**.

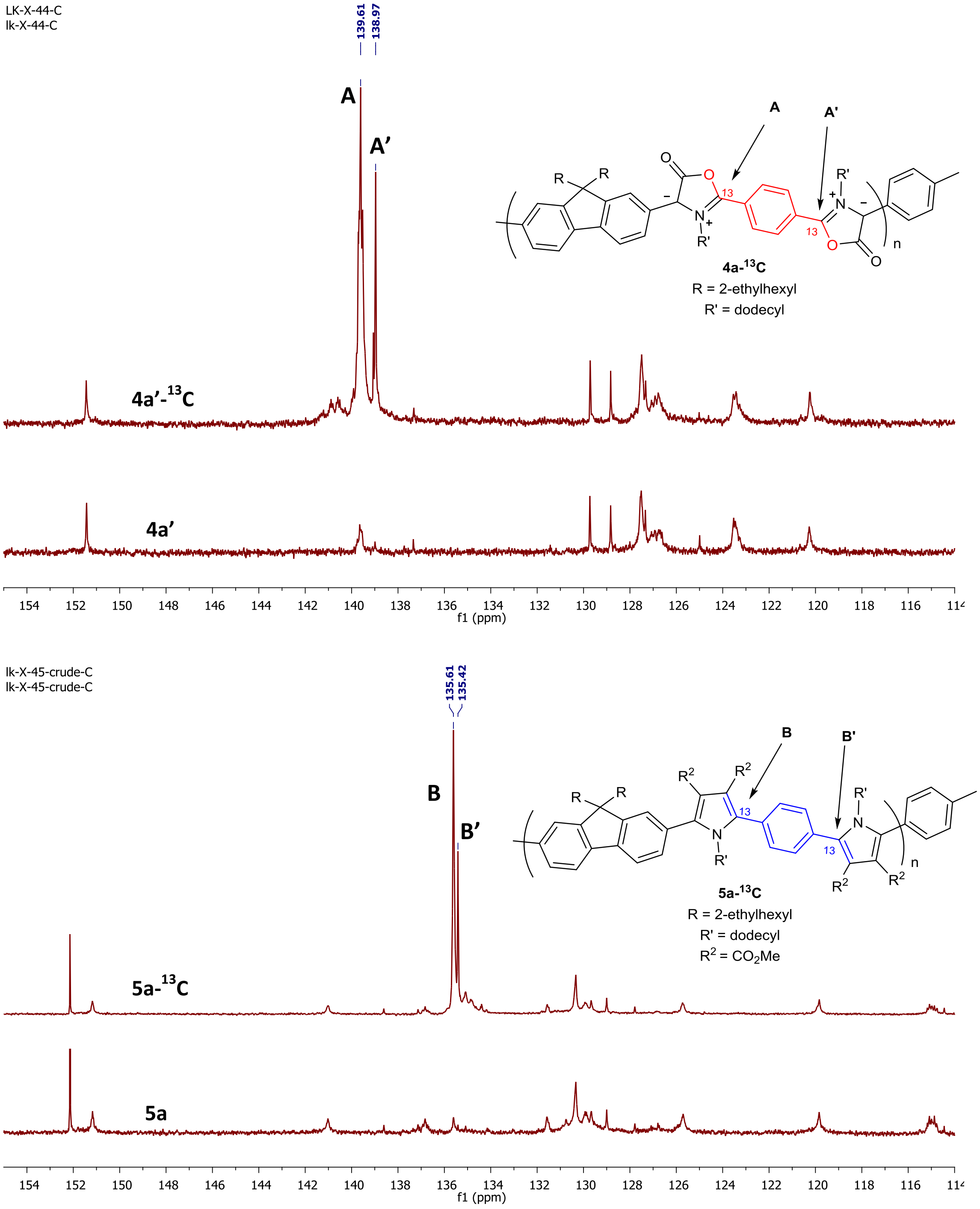

Supplementary Figure 33. ^{13}C NMR spectra of **4a'-^{13}C, 4a', 5a-^{13}C** and **5a** for the quantification of the cycloaddition.

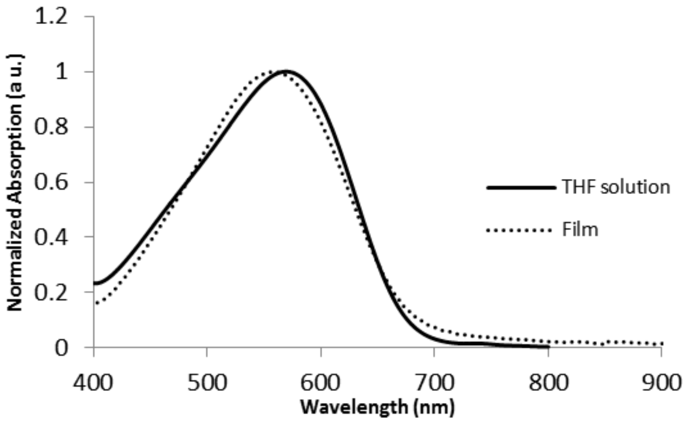

Supplementary Figure 34. UV/Vis absorption spectra of polymer **4a'**.

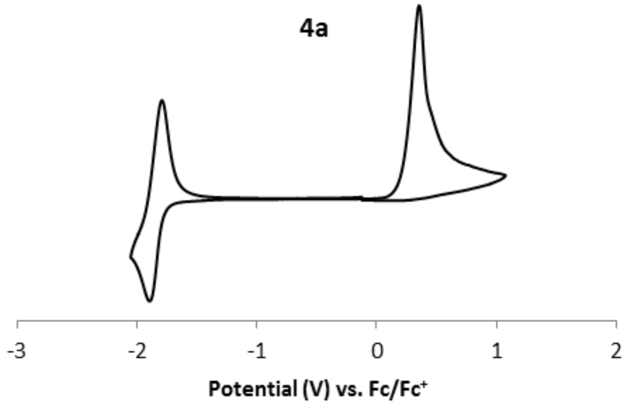

Supplementary Figure 35. Cyclic Voltammogram of polymer **4a'**.

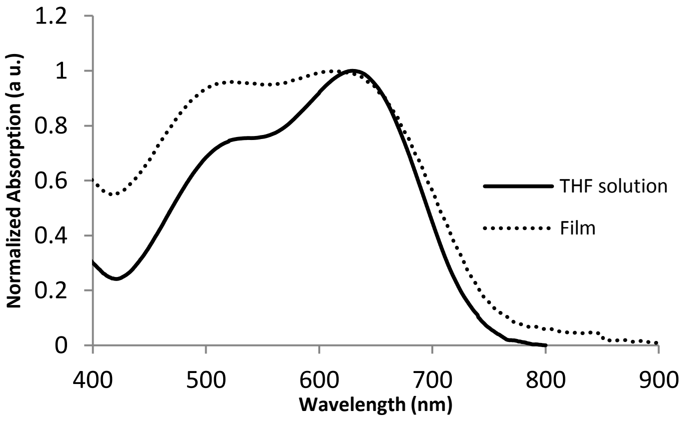

Supplementary Figure 36. UV/Vis absorption spectra of polymer **4b'**.

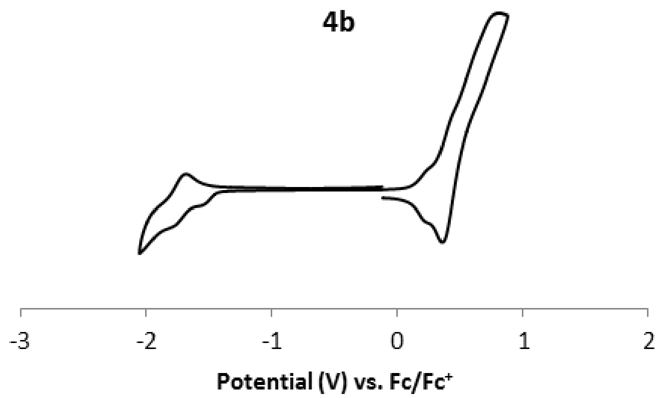

Supplementary Figure 37. Cyclic Voltammogram of polymer **4b'**.

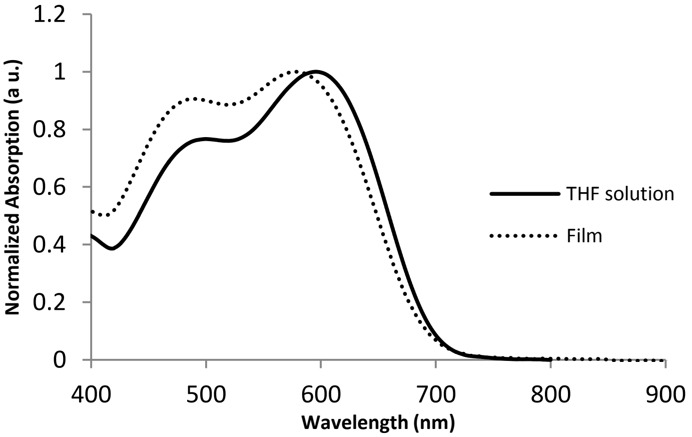

Supplementary Figure 38. UV/Vis absorption spectra of polymer **4c'**.

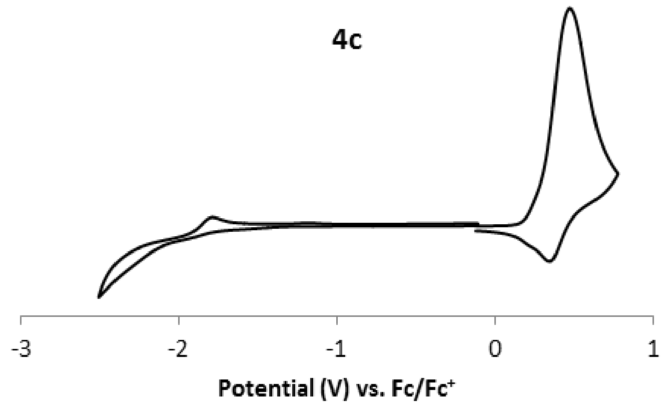

Supplementary Figure 39. Cyclic Voltammogram of polymer **4c'**.

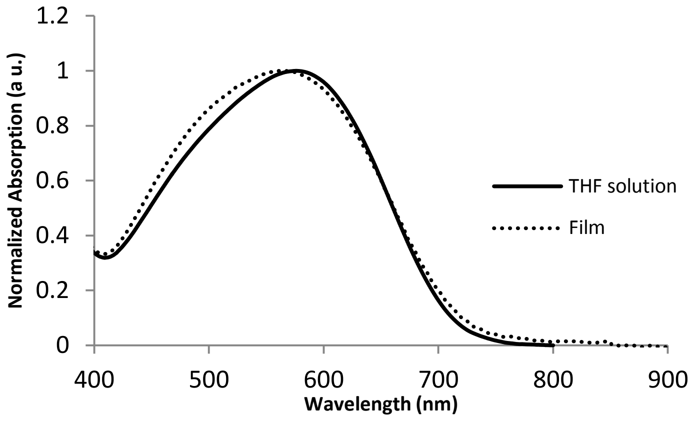

Supplementary Figure 40. UV/Vis absorption spectra of polymer **4d'**.

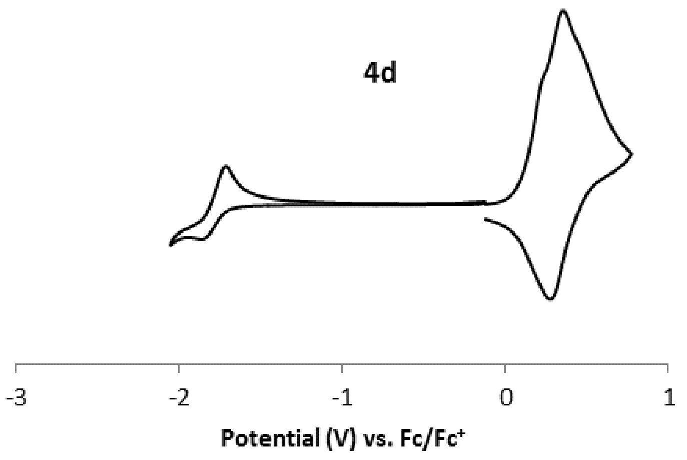

Supplementary Figure 41. Cyclic Voltammogram of polymer **4d'**.

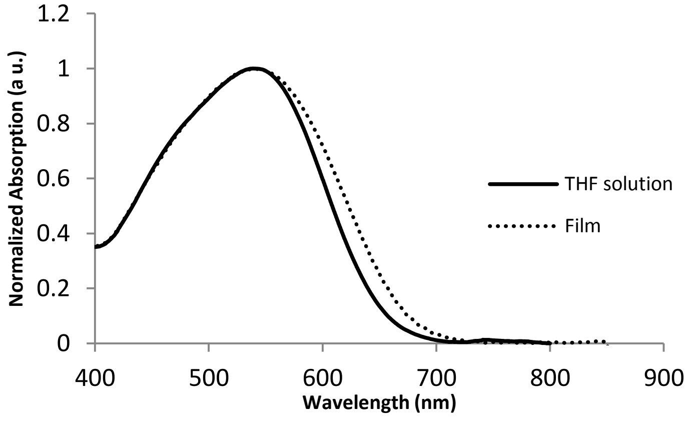

Supplementary Figure 42. UV/Vis absorption spectra of polymer **4e'**.

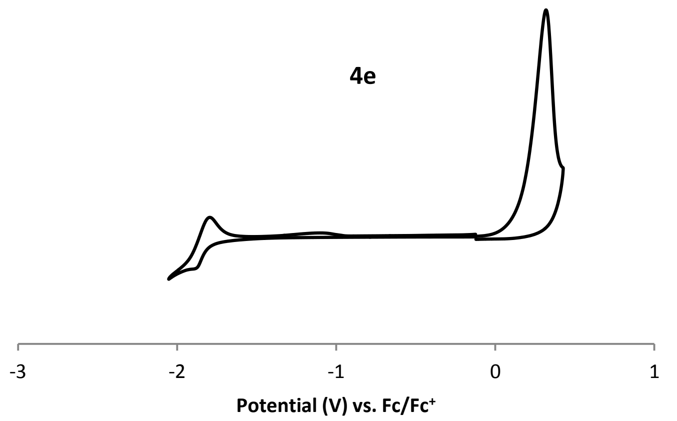

Supplementary Figure 43. Cyclic Voltammogram of polymer **4e'**.

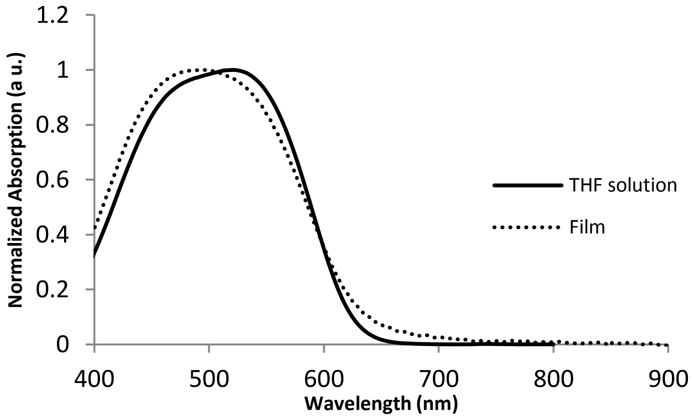

Supplementary Figure 44. UV/Vis absorption spectra of polymer **4f'**.

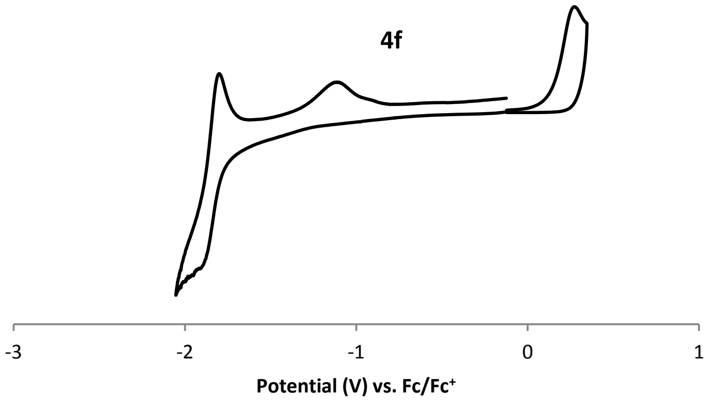

Supplementary Figure 45. Cyclic Voltammogram of polymer **4f'**.

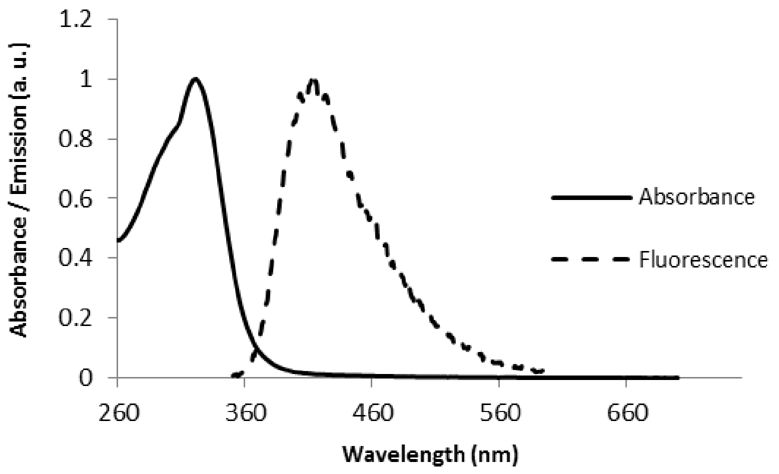

Supplementary Figure 46. UV/Vis absorption and fluorescence emission spectra of polymer **5a**.

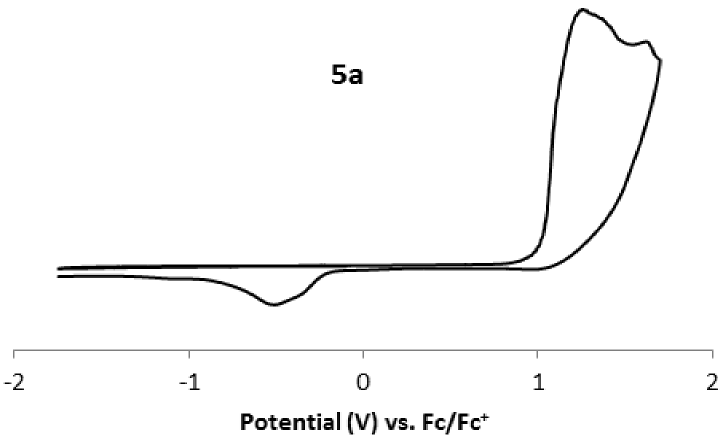

Supplementary Figure 47. Cyclic Voltammogram of polymer **5a**.

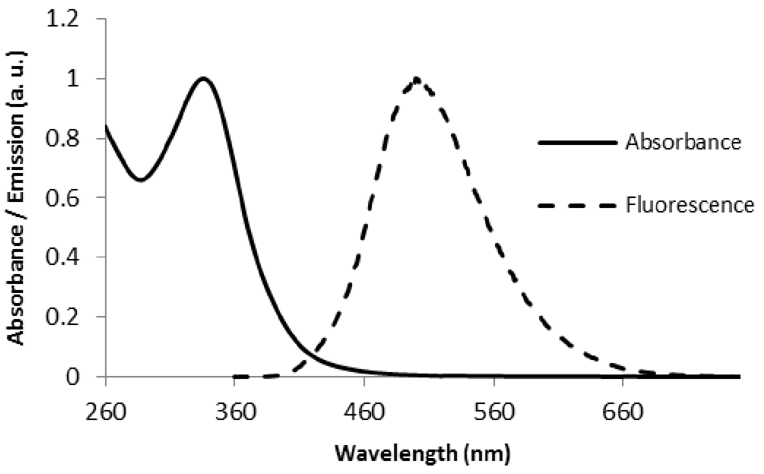

Supplementary Figure 48. UV/Vis absorption and fluorescence emission spectra of polymer **5b**.

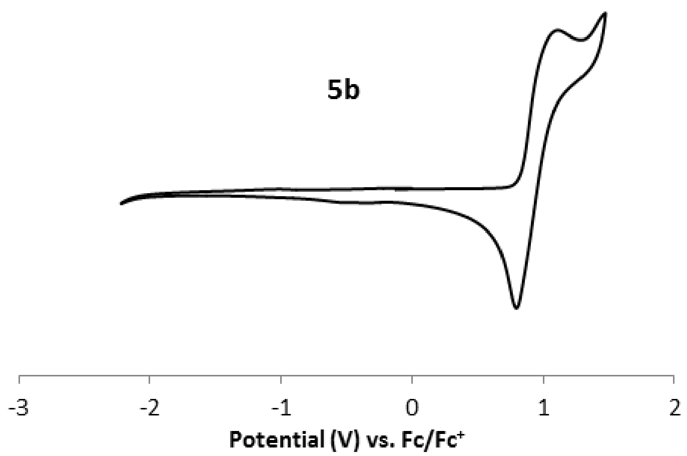

Supplementary Figure 49. Cyclic Voltammogram of polymer **5b**.

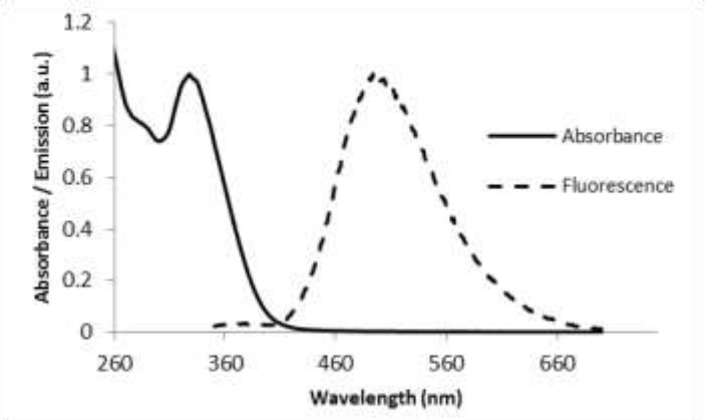

Supplementary Figure 50. UV/Vis absorption and fluorescence emission spectra of polymer **5c.**

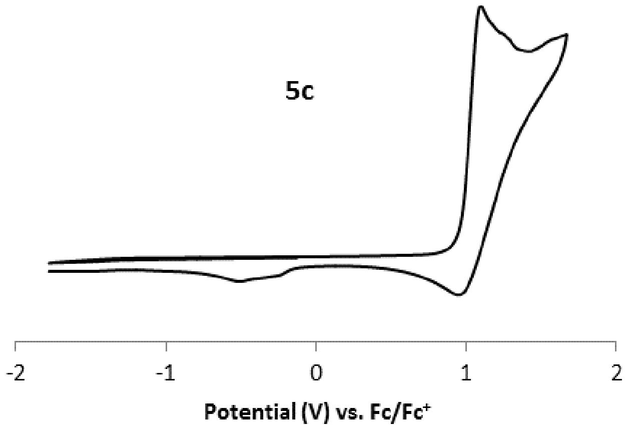

Supplementary Figure 51. Cyclic Voltammogram of polymer **5c**.

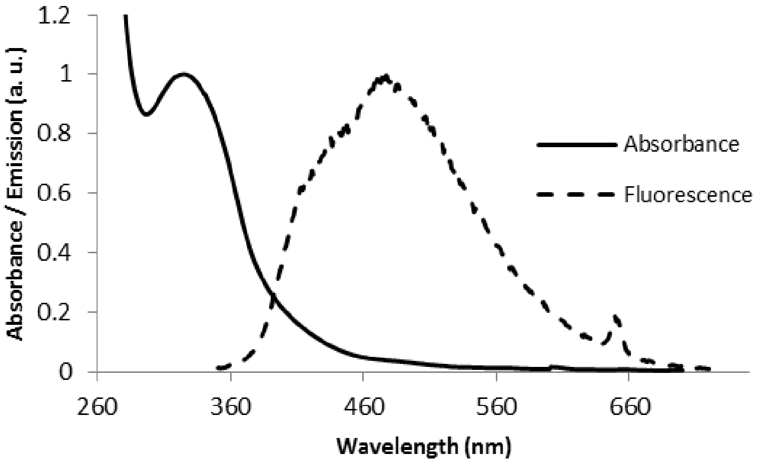

Supplementary Figure 52. UV/Vis absorption and fluorescence emission spectra of polymer **5d.**

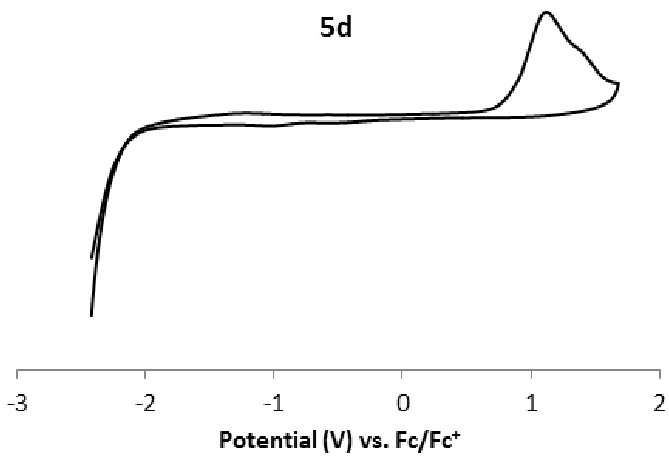

Supplementary Figure 53. Cyclic Voltammogram of polymer **5d**.

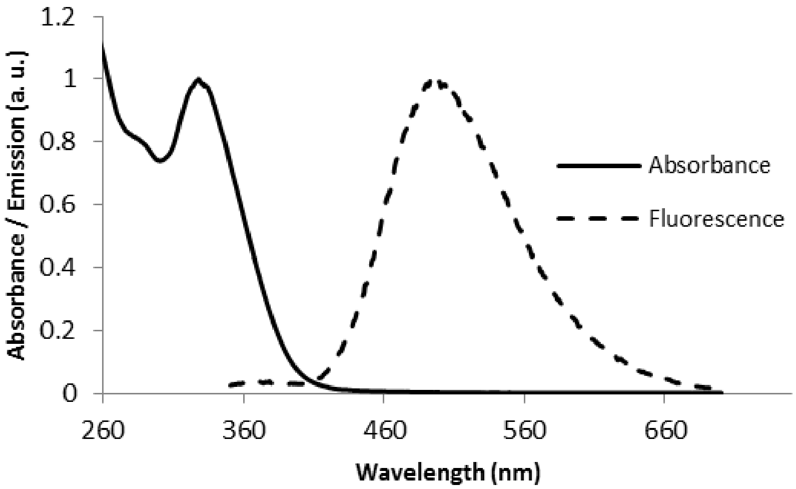

Supplementary Figure 54. UV/Vis absorption and fluorescence emission spectra of polymer **5e.**

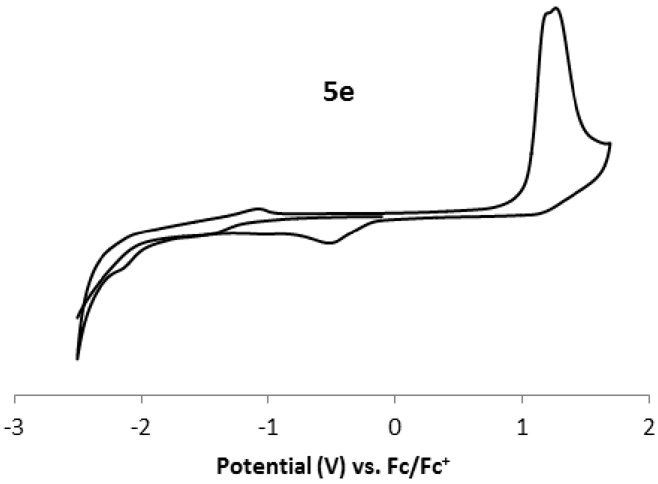

Supplementary Figure 55. Cyclic Voltammogram of polymer **5e**.

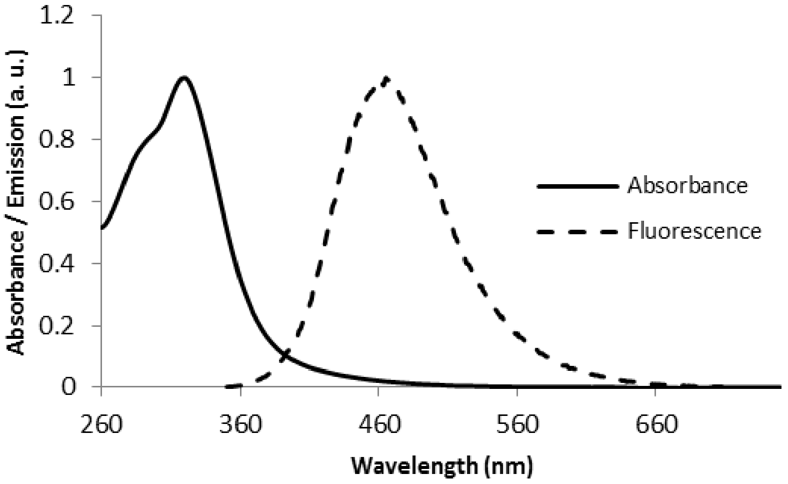

Supplementary Figure 56. UV/Vis absorption and fluorescence emission spectra of polymer **5f.**

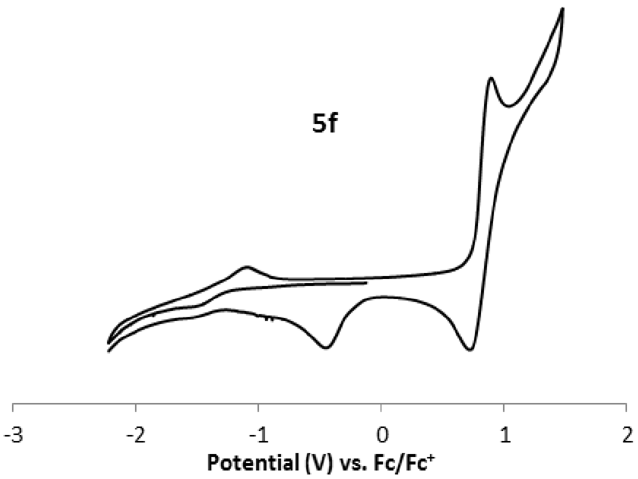

Supplementary Figure 57. Cyclic Voltammogram of polymer **5f**.

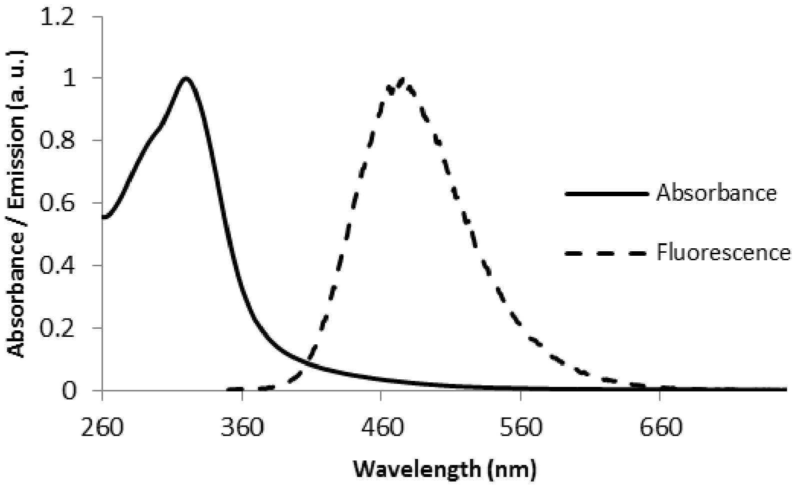

Supplementary Figure 58. UV/Vis absorption and fluorescence emission spectra of polymer **5g.**

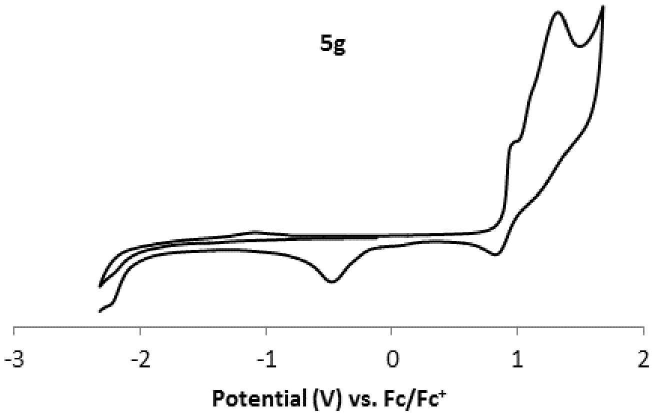

Supplementary Figure 59. Cyclic Voltammogram of polymer **5g**.

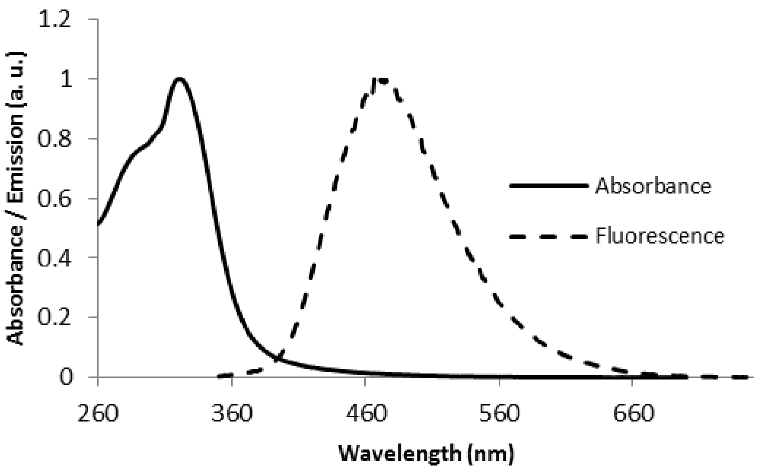

Supplementary Figure 60. UV/Vis absorption and fluorescence emission spectra of polymer **5h.**

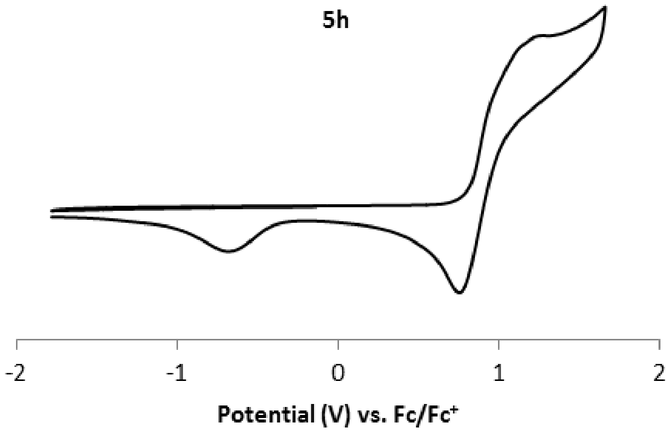

Supplementary Figure 61. Cyclic Voltammogram of polymer **5h**.

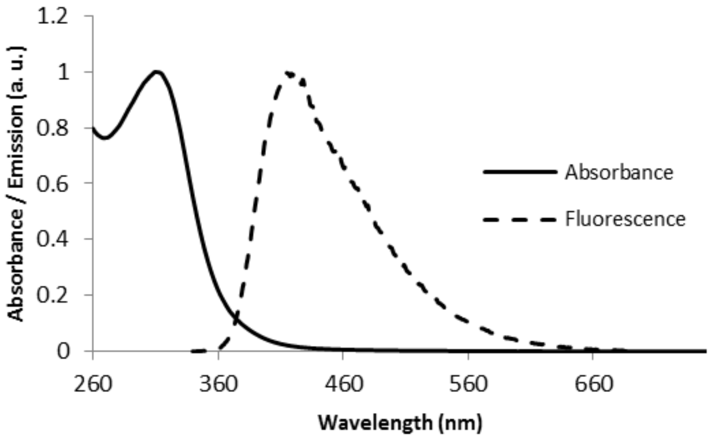

Supplementary Figure 62. UV/Vis absorption and fluorescence emission spectra of polymer **5i.**

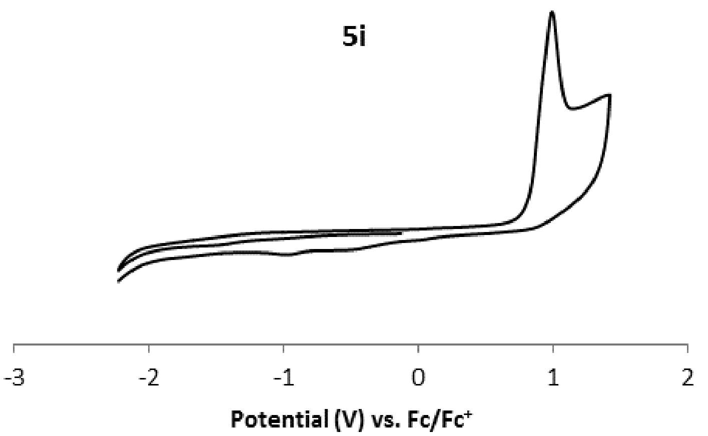

Supplementary Figure 63. Cyclic Voltammogram of polymer **5i**.

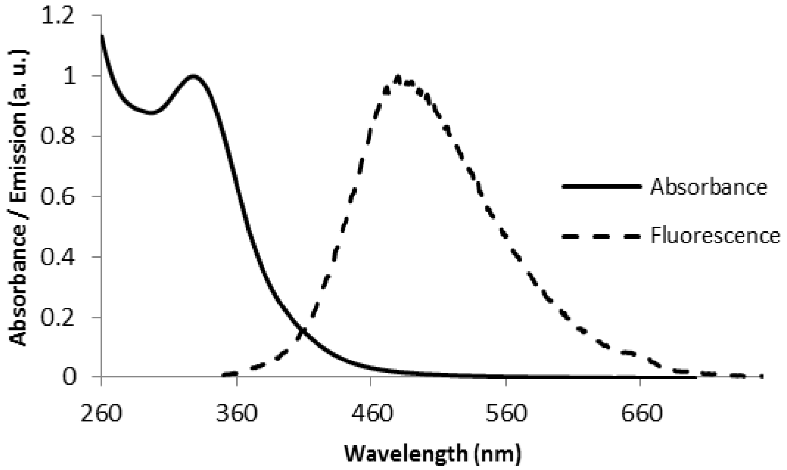

Supplementary Figure 64. UV/Vis absorption and fluorescence emission spectra of polymer **5j.**

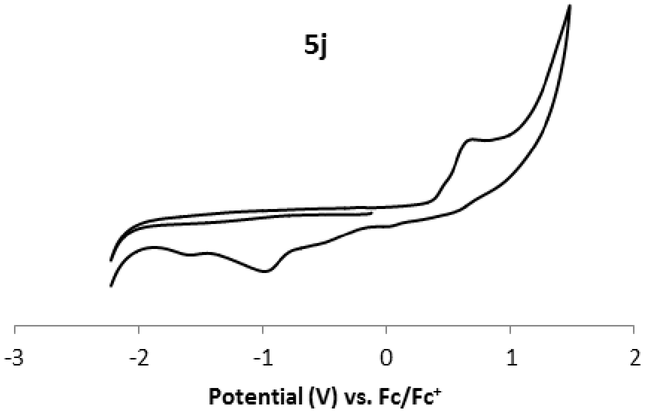

Supplementary Figure 65. Cyclic Voltammogram of polymer **5j**.

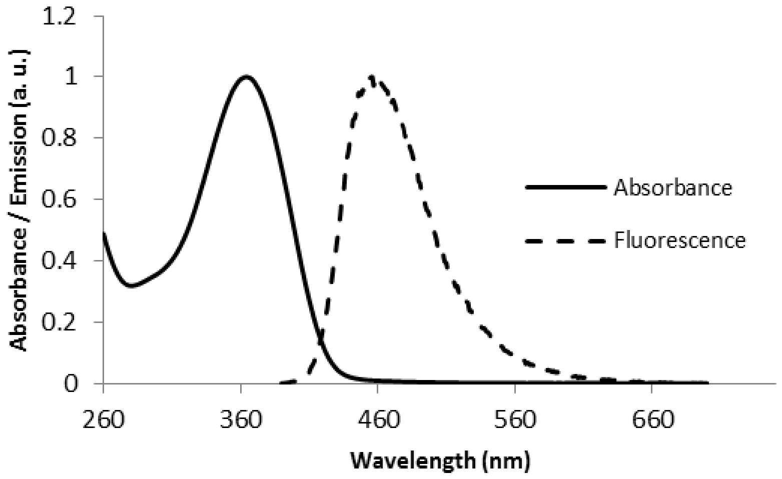

Supplementary Figure 66. UV/Vis absorption and fluorescence emission spectra of polymer **6a.**

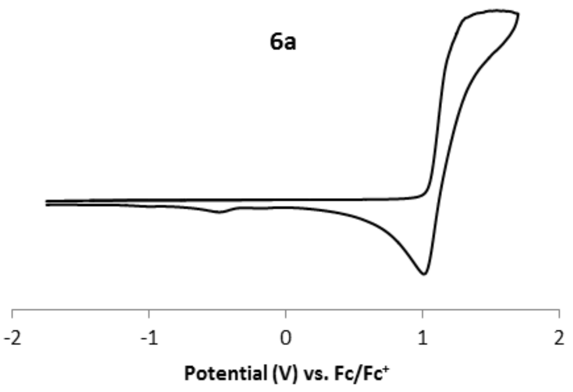

Supplementary Figure 67. Cyclic Voltammogram of polymer **6a**.

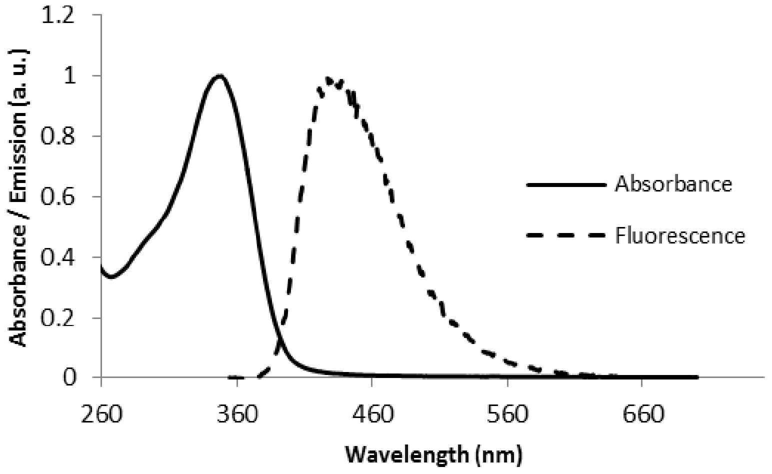

Supplementary Figure 68. UV/Vis absorption and fluorescence emission spectra of polymer **6b.**

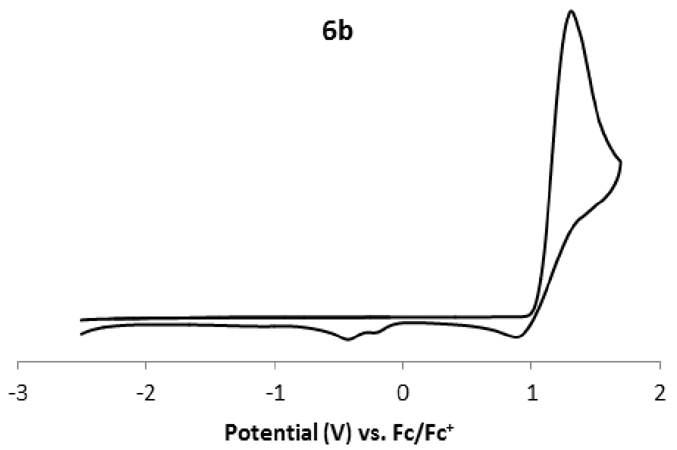

Supplementary Figure 69. Cyclic Voltammogram of polymer **6b**.

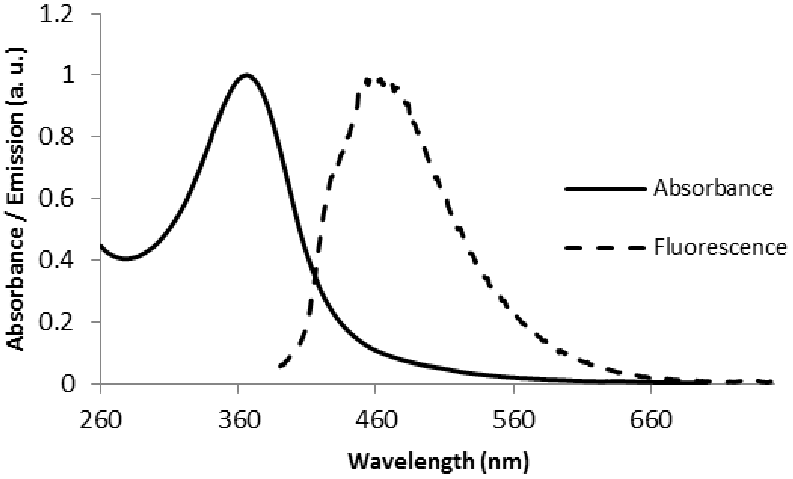

Supplementary Figure 70. UV/Vis absorption and fluorescence emission spectra of polymer **6c.**

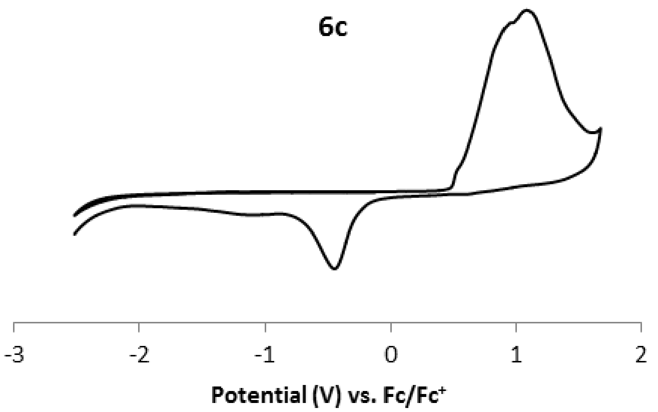

Supplementary Figure 71. Cyclic Voltammogram of polymer **6c**.

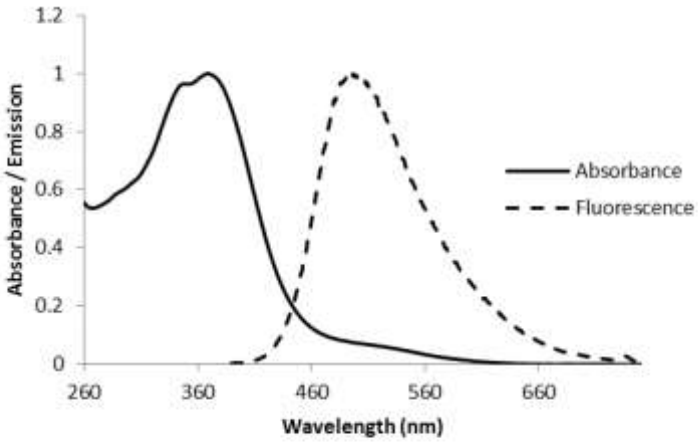

Supplementary Figure 72. UV/Vis absorption and fluorescence emission spectra of polymer **6d.**

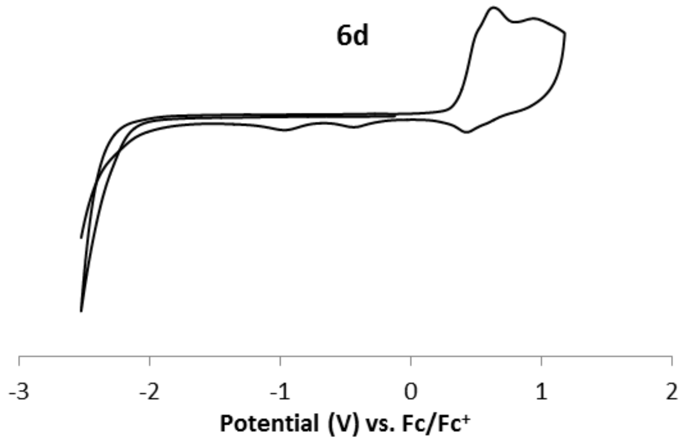

Supplementary Figure 73. Cyclic Voltammogram of polymer **6d**.

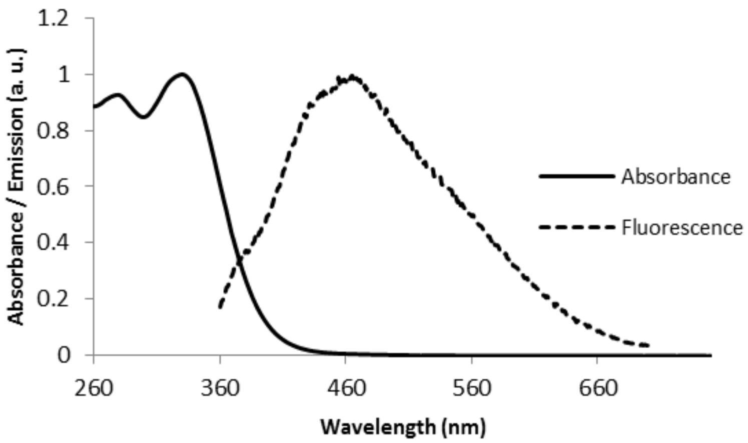

Supplementary Figure 74. UV/Vis absorption and fluorescence emission spectra of polymer **7.**

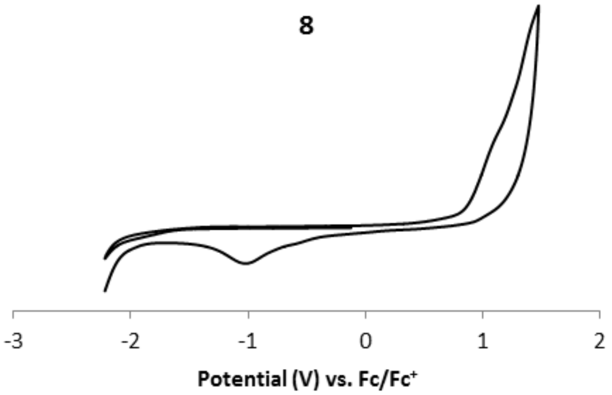

Supplementary Figure 75. Cyclic Voltammogram of polymer **7**.

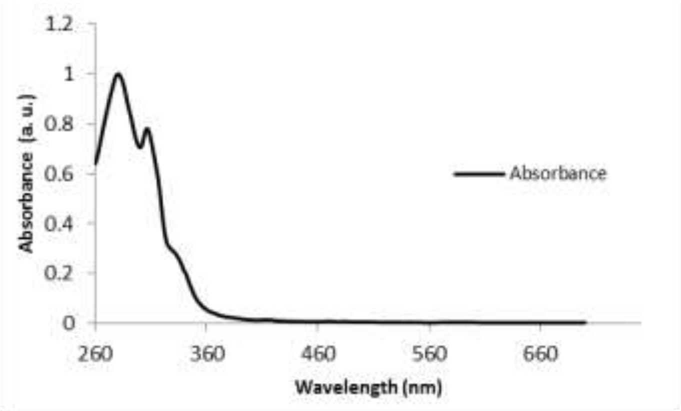

Supplementary Figure 76. UV/Vis absorption spectra of polymer **8.**

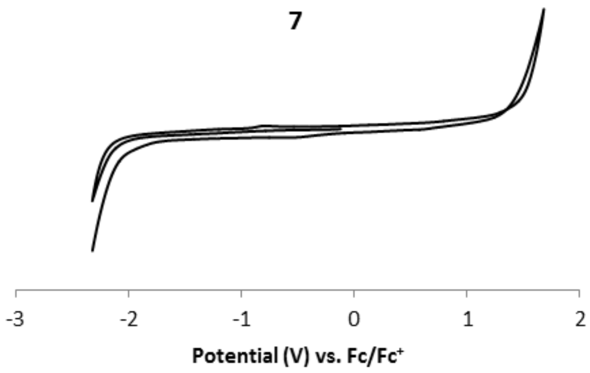

Supplementary Figure 77. Cyclic Voltammogram of polymer **8**.

Supplementary Tables

Supplementary Table 1. Properties of poly-Münchnones **4a-f**[a]

Cpd	λ_{max}[b] (nm)	λ_{onset} (nm)	E_g^{opt} (eV)	E_g^{opt} film[c] (eV)	E_g^{elec} (eV)	E_{HOMO}[d] (eV)	E_{LUMO}[e] (eV)
**4a**	570	699	1.78	1.74	1.60	-4.92	-3.08
**4b**	630	768	1.62	1.58	1.29	-4.91	-3.40
**4c**	596	725	1.71	1.72	1.46	-5.03	-3.11
**4d**	576	749	1.66	1.65	1.56	-4.88	-3.16
**4e**	539	688	1.81	1.76	1.59	-4.90	-3.11
**4f**	521	649	1.91	1.88	1.66	-4.84	-3.09

[a]Analyses of **4a-f** were performed on imine end-capped materials to ensure complete solubility. [b]THF solution. [c]Drop-cast polymer thin film. [d] From cathodic onset with reference to Fc/Fc^+ (E_{HOMO} = e - ($E\text{ox}^{onset}$ vs Fc/Fc^+) - 4.80 eV)[1] [e] From anodic onset with reference to Fc/Fc^+ (E_{LUMO} = e - ($E\text{red}^{onset}$ vs Fc/Fc^+) - 4.80 eV).

Supplementary Table 2. Properties of polymers **5-8**

Cpd	M_n (kDa)	PDI	λ_{max}[a] (nm)	λ_{onset} (nm)	λ_{em} (nm)	φ_{PL}	E_g^{opt} (eV)	E_g^{opt} film[b] (eV)	E_{HOMO}[c] (eV)	E_{LUMO}[d] (eV)
**5a**	22.7[e]	2.5	321	391	413	0.39	3.18	3.16	-5.67	-2.51
**5b**	11.1	2.1	335	426	492	0.27	2.91	2.87	-5.57	-2.70
**5c**	12.2	2.1	329	416	496	0.04	2.99	2.98	-5.72	-2.74
**5d**	9.4	2.5	325	426	474	0.03	2.92	2.79	-5.47	-2.68
**5e**	8.2	2.1	301	395	413	0.12	3.15	3.08	-5.75	-2.67
**5f**	12.5[e]	2.6	320	403	459	0.14	3.08	2.97	-5.45	-2.48
**5g**	10.8	2.8	320	405	467	0.11	3.07	3.11	-5.61	-2.50
**5h**	12.8[e]	2.6	321	408	467	0.10	3.05	2.97	-5.51	-2.54
**5i**	8.5	1.6	312	390	417	0.17	3.19	2.93	-5.53	-2.60
**5j**	8.5	1.6	329	431	473	0.02	2.88	2.92	-5.16	-2.24
**6a**	11.4	2.4	364	434	459	0.35	2.86	2.80	-5.75	-2.95
**6b**	9.8	2.3	345	413	431	0.47	3.01	2.96	-5.80	-2.84
**6c**	8.1	1.8	366	478	501	0.08	2.60	2.60	-5.27	-2.67
**6d**	6.1	2.1	368	472	497	0.06	2.63	2.47	-5.07	-2.60
**7**	9.2	1.4	330	417	467	0.12	2.98	2.80	-5.62	-2.82
**8**	11.5	2.0	280	360	-	-	3.45	3.42	-	-

[a] $CHCl_3$ solution. [b]Drop-cast film. [c] Calculated as in Table c. [d] The anodic onset was not observed. Therefore electron affinity was calculated from the HOMO level and optical band-gap (E_{LUMO} = E_{HOMO} + E_g^{opt} thin film). [e] The formation of **4** was performed for 64h.

Supplementary Methods

General methods. All reactions were carried out using standard Schlenk line and glovebox techniques under an atmosphere of oxygen- and water-free dinitrogen, unless described otherwise. Solution phase ^{1}H and $^{13}C\{^{1}H\}$ NMR spectra were recorded on a 300 MHz Varian Mercury, 400 MHz Varian Mercury, or 500 MHz Varian VNMRS spectrometer at ambient temperature; chemical shifts are reported in parts per million (ppm) relative to the corresponding residual *protio*-solvent signal. Mass spectra were acquired by electrospray ionization (ESI). GPC was carried out on a Polymer Laboratories PL-GPC 50 with THF as the eluent and a UV/Vis absorbance detector. Samples were analyzed versus monodispersed polystyrene standards. The UV/Vis absorption of the polymers was measured in THF (polymers **4**) or $CHCl_3$ (polymers **5-8**) solutions using a 1 cm path quartz cell and also as thin films (drop cast on a glass slide) using a JASCO V670 UV-Vis-NIR spectrometer. The fluorescence measurements were performed on a Varian Eclipse Fluorometer. The fluorescence quantum yields were determined versus anthracene in ethanol (Φ_{em} = 0.27) at slit widths of 2.5 nm. Cyclic voltammetry was performed on a CH670 potentiostat from CH-Instruments in a three-electrode cell using a 0.1M solution of $(TBA)PF_6$ in CH_3CN as an electrolyte. Platinum wires were used as working and counter electrodes, a $Ag/AgNO_3$ electrode was used as a reference. The scan rate was 0.1 V s^{-1} for all the measurements. All potentials were adjusted versus ferrocene (Fc/Fc^{+}). The reduction and oxidation in cyclic voltammograms of polymers **4** were acquired separately. UV/Vis, Fluorescence spectra and cyclic voltammograms for the polymers are shown in Supplementary Figures 34-77.

Materials. All common reagents were purchased from Aldrich and used as received, unless otherwise noted. Na_2PdCl_4 was purchased from Pressure Chemicals. P(*o*-tol)$_3$ and P(*t*-Bu)$_3$ $PdCl_2(PhCN)_2$, and $PdCl_2(PPh_3)_2$ were purchased from Strem and used as received. Common solvents (THF, Et_2O, acetonitrile, DCM) were sparged with dinitrogen and dried by passage through a column of alumina before use in air- and moisture-sensitive experiments. *d*^{6}-Benzene and $CDCl_3$ were dried over CaH_2 for at least 48 hours, then degassed by the freeze-pump-thaw method and vacuum transferred prior to use. *N*,*N*-Diisopropylethylamine, dimethyl acetylenedicarboxylate, methyl phenylpropiolate, 2-chloroacrylonitrile were distilled from CaH_2 prior to use in

polymerizations. Terephthaloyl chloride was recrystallized from hexanes prior to use in polymerizations. $Pd_2dba_3CHCl_3$,[2] Pd[P(*o*-tol)$_3$],[3] Pd[P(*t*-Bu)$_3$]$_2$,[4] 9,9-Bis(2-ethylhexyl)-2,7-fluorene dicarboxaldehyde,[5] *N*-(2-ethylhexyl)-2,7-carbazole dicarboxaldehyde,[6] 2,5-furan dicarbonyl chloride,[7] 2,5-thiophene dicarbonyl chloride,[8] 1,4-diphenylbut-2-yne-1,4-dione,[9] and 3-bromo-1-hexyl-1H-pyrrole-2,5-dione[10] were prepared according to literature procedures.

Synthesis of monomer 2a.

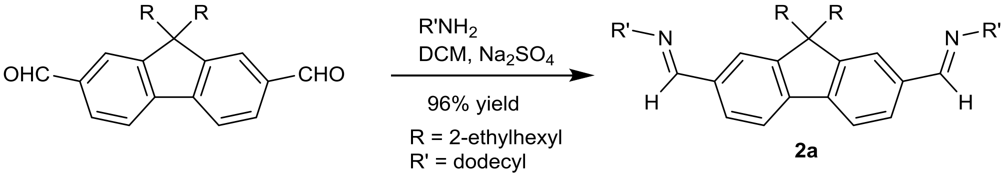

2a: 9,9-Bis(2-ethylhexyl)-2,7-fluorene dicarboxaldehyde (6.53 g, 14.6 mmol) and dodecylamine (5.42 g, 29.2 mmol) were dissolved in 50 mL dichloromethane. Excess Na_2SO_4 was added and the mixture stirred for 3 hours. CaH_2 was carefully added portionwise until no more effervescence was observed, then an approximate equal amount of Celite was added. The suspension was gravity filtered and the solvent removed. The resulting viscous oil was dried under vacuum at 70 °C for 18 hours to give 11.00 g of pure diimine (96% yield). ^{1}H NMR (400 MHz, $CDCl_3$) δ 8.30 (s, 2H), 7.73 (m, 6H), 3.63 (t, *J* = 7.0 Hz, 4H), 2.03 (m, 4H), 1.70 (m, 4H), 1.52 – 1.11 (m, 36H), 0.99 – 0.58 (m, 26H), 0.58 – 0.41 (m, 6H). ^{13}C NMR (100 MHz, $CDCl_3$) δ 160.9, 151.6, 143.0, 135.1, 127.5, 123.5, 120.1, 62.0, 54.9, 44.3, 34.7, 33.5, 31.9, 31.0, 29.6, 29.4, 29.4, 27.9, 27.4, 27.0, 22.7, 14.1, 14.0, 10.3. MS(ESI): $C_{55}H_{93}N_2$ $[M+H]^+$ *m/z* calcd. 781.73333; found 781.73251. See Supplementary Figure 1 for ^{1}H and ^{13}C NMR spectra.

Synthesis of monomer 2b.

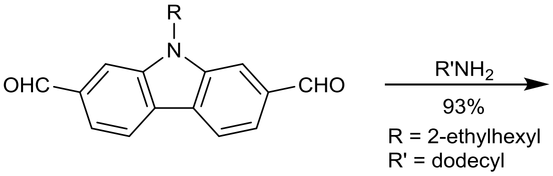

2b: *N*-(2-ethylhexyl)-2,7-carbazole dicarboxaldehyde (0.150 g, 0.447 mmol) and dodecylamine (0.158 g, 0.852 mmol) were dissolved in dichloromethane (2 mL). Excess Na_2SO_4 was added and the mixture stirred for 5 hours. The solution was filtered and a fresh portion of Na_2SO_4 was added. The suspension was stirred overnight. The solution was filtered and the solvent removed to give a waxy solid. This material was dried *in vacuo* at 70 °C overnight (note: solid melts at this temperature) to give a waxy off-white solid upon cooling (0.278 g, 93% yield). ^1H NMR (400 MHz, $CDCl_3$) δ 8.44 (s, 2H), 8.10 (d, *J* = 8.0 Hz, 2H), 7.78 (s, 2H), 7.62 (d, *J* = 8.1 Hz, 2H), 4.24 (m, 2H), 3.67 (t, *J* = 7.0 Hz, 4H), 2.25 – 2.00 (m, 1H), 1.86 – 1.59 (m, 4H), 1.52 – 1.06 (m, 46H), 0.88 (m, 12H). ^{13}C NMR (100 MHz, $CDCl_3$) δ 161.4, 141.9, 134.5, 124.4, 120.6, 119.5, 108.7, 61.9, 47.5, 39.2, 31.9, 31.1, 30.8, 29.7, 29.7, 29.6, 29.5, 29.4, 28.6, 27.4, 24.3, 23.0, 22.7, 14.1, 14.0, 10.9. MS(ESI): $C_{46}H_{76}N_3$ $[M+H]^+$ *m*/*z* calcd. 670.60338; found 670.60324. See Supplementary Figure 2 for ^1H and ^{13}C NMR spectra.

Synthesis of monomer 2c.

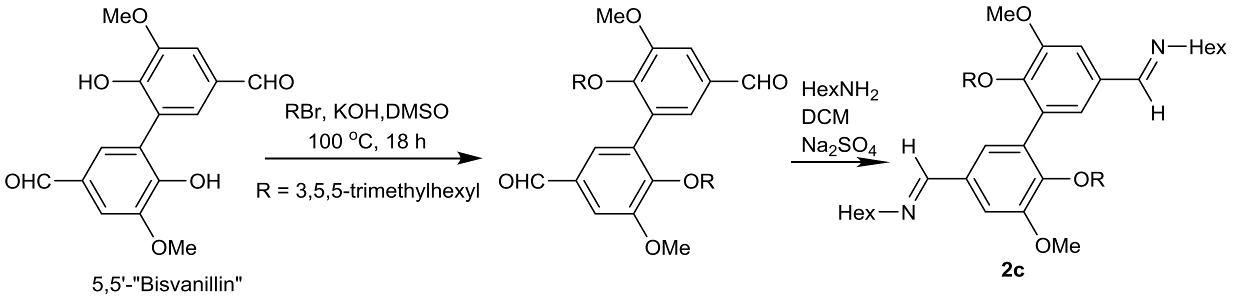

2c: 5,5'-"Bisvanillin" [11] (2.50 g, 8.270 mmol) and KOH (2.125g, 37.87 mmol) were dissolved in DMSO (40 mL) at 80 °C. After 1 hour, 1-bromo-3,5,5-trimethylhexane were added. The mixture was stirred at 80 °C for 18 hours. The product was extracted with ether. The ether layer was washed with water and brine and then dried over sodium sulfate. After evaporated in vacuum, the crude product was purified by column chromatography. The resulting alkylated dialdehyde (1.045 g, 1.890 mmol) was dissolved in dichloromethane (3 mL). Hexylamine (0.383 g, 0.500 mL, 3.78 mmol) was added via syringe. Excess Na_2SO_4 was added and the mixture stirred for 5 hours. The solution was

filtered and a fresh portion of Na_2SO_4 was added. The suspension was stirred overnight. The solution was filtered and the solvent removed to give a viscous yellow oil. This material was dried *in vacuo* at 70 °C overnight to give **2c** (1.276 g, 94% yield). ^1H NMR (400 MHz, $CDCl_3$) δ 8.15 (s, 2H), 7.44 (d, *J* = 1.4 Hz, 2H), 7.10 (m, 2H), 3.92 (s, 6H) 3.90 – 3.84 (m, 2H), 3.83 – 3.73 (m, 2H), 3.57 (t, *J* = 7.0 Hz, 4H), 1.66 (m, 4H), 1.54 (m, 2H), 1.40 – 1.14 (m, 10H), 1.05 (dd, *J* = 13.9, 3.8 Hz, 2H), 0.90 (m, 6H), 0.81 – 0.74 (m, 16H), 0.74 – 0.67 (m, 6H). ^{13}C NMR (100 MHz, $CDCl_3$) δ 160.3, 153.3, 148.5, 132.3, 131.4, 125.5, 108.7, 71.5, 61.7, 55.8, 51.4, 39.4, 31.7, 31.0, 29.9, 27.2, 27.0, 25.8, 25.7, 22.6, 22.2, 22.1, 14.1. MS(ESI): $C_{46}H_{77}O_4N_2$ $[M+H]^+$ *m/z* calcd. 721.58779; found 721.58677. See Supplementary Figure 3 for ^1H and ^{13}C NMR spectra.

Synthesis of model compounds.

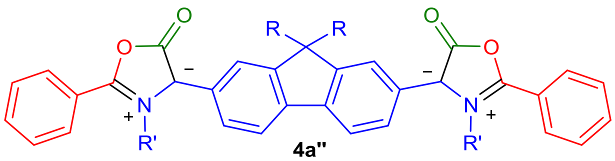

4a"

R = 2-ethylhexyl
R' = dodecyl

4a”: A model bis(Münchnone) based on polymer **4a** was prepared as follows. Imine **2a** (122.2 mg, 0.200 mmol) and benzoyl chloride (56.2 mg, 46.5 µL, 0.400 mmol) were dissolved in THF (1 mL) in a Teflon-sealable thick walled reaction vessel. Then, *N*,*N*-diisopropylethylamine (103.4 mg, 145.2 µL, 0.800 mmol) and Pd[P(*o*-tol)$_3$]$_2$ (14.3 mg, 0.0200 mmol) were dissolved/suspended in THF (1 mL). This slurry was added to the imine/acid chloride solution and stirred until homogeneous. The headspace was briefly evacuated, and the reaction tube charged with 60 psi CO (gauge pressure). The mixture was heated to 50 °C for 18 hours with stirring. Afterward, the CO headspace was replaced with nitrogen, and the vessel returned to the glovebox. The resulting suspension was diluted with dichloromethane (5 mL) and transferred to a 20 mL glass vial. Excess K_3PO_4 was added and the suspension vigorously stirred for 18 hours. The suspension was filtered through Celite and the filter cake washed with dichloromethane (3 x 2 mL). The volatiles were removed *in vacuo* to give the crude product. The residue was dissolved in a minimum of acetonitrile and cooled to -35 °C to precipitate 35.2 mg of **4a”** (20% yield, significant product was sacrificed to obtain a pure sample; a crude ^1H NMR spectrum indicated complete conversion). See Supplementary Figures 1-9 for ^1H and ^{13}C

NMR spectra of all model compounds. ^{1}H NMR (400 MHz, $CDCl_3$) δ 7.70 (dd, *J* = 16.6, 7.2 Hz, 6H), 7.62 – 7.36 (m, 10H), 4.36 (s, 4H), 2.03 (s, 4H), 1.56 (d, *J* = 6.5 Hz, 2H), 1.36 – 0.60 (m, 40H), 0.52 (t, *J* = 7.2 Hz, 3H). ^{13}C NMR (75 MHz, $CDCl_3$) δ 161.1, 151.2, 142.7, 139.2, 130.9, 129.4, 127.9, 127.3, 126.7, 126.6, 126.4, 123.4, 123.2, 123.1, 122.9, 120.0, 95.9, 55.0, 47.4, 45.3, 34.7, 33.5, 30.1, 28.8, 28.2, 26.5, 25.5, 22.8, 22.3, 14.0, 13.8, 10.2. MS(ESI): $C_{59}H_{77}O_4N_2$ $[M+H]^+$ *m*/*z* calcd. 877.58779; found 877.58724.

5a', 5b', 5c', 6a', 6b': Model bis(pyrrole)s were formed for spectroscopic comparison to polymers **5** and **6** in order to confirm the presence of the newly constructed pyrrole units. These compounds were obtained as follows. Imine **2a** (234.4 mg, 0.300 mmol) and benzoyl chloride (84.3 mg, 69.6 µL, 0.600 mmol) were dissolved in THF (1.5 mL) in a Teflon-sealable thick walled reaction vessel. Then, *N*,*N*-diisopropylethylamine (155.1 mg, 209.0 µL, 1.20 mmol) and $Pd[P(o\text{-tol})_3]_2$ (21.5 mg, 0.0300 mmol) were dissolved/suspended in THF (1.5 mL). This slurry was added to the imine/acid chloride solution and stirred until homogeneous. The headspace was briefly evacuated, and the reaction tube charged with 60 psi CO (gauge pressure). The mixture was heated to 50 °C for 24 hours with stirring. Afterward, the CO headspace was replaced with nitrogen, and the vessel returned to the glovebox. The mixture was diluted with THF (total volume ~6 mL) and split into six fractions, one for each of the model compounds. To each fraction was added a dipolarophile in the following amounts: dimethyl but-2-ynedioate: 28.4 mg, 24.6 µL, 0.200 mmol ethyl 3-phenylpropiolate: 32.0 mg, 29.5 µL, 0.200 mmol; 1,4-diphenylbut-2-yne-1,4-dione: 46.7 mg, 0.200 mmol; 3-bromo-1-hexyl-1H-pyrrole-2,5-dione: 28.6 mg, 0.110 mmol (also added *N*,*N*-diisopropylethylamine [12.9 mg, 17.4 µL, 0.100 mmol]); 2-chloroacrylonitrile: 11.0 mg, 10.0 µL, 0.126 mmol (also added *N*,*N*-diisopropylethylamine [12.9 mg, 17.4 µL, 0.100 mmol]).

The reactions were stirred at room temperature for 18 hours, except for that with 1,4-diphenylbut-2-yne-1,4-dione, which was heated to 50 °C for 18 hours. After the allotted reaction time, each solution was concentrated *in vacuo* and purified by flash chromatography on silica gel (details for each below). Because the six-way split of the solution of di(Münchnone), the reactions were not performed quantitatively; these compounds were sought just as models for spectroscopic comparisons with polymers **5**

and **6**. The yields for the individual runs varied considerably, and were judged not representative of the reaction efficiency; the average yield was 82%.

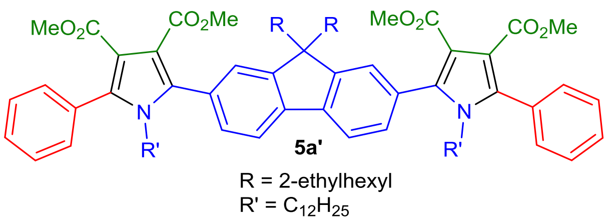

5a': ^{1}H NMR (500 MHz, $CDCl_3$) δ 7.80 (d, *J* = 7.0 Hz, 2H), 7.62 – 7.33 (m, 14H), 3.77 – 3.68 (m, 4H), 3.66 (s, 6H), 3.63 – 3.54 (m, 6H), 2.03 (m, 4H), 1.40 – 1.09 (m, 26H), 1.09 – 1.01 (m, 3H), 1.01 – 0.91 (m, 7H), 0.91 – 0.60 (m, 28H), 0.50 (t, *J* = 7.2 Hz, 6H). ^{13}C NMR (125 MHz, $CDCl_3$) δ 165.5, 165.4, 151.1, 141.0, 136.6, 136.4, 131.1, 130.5, 129.8, 128.7, 128.2, 125.8, 125.7, 119.8, 114.8, 114.3, 55.0, 51.6, 51.4, 45.6, 45.0, 34.8, 33.4, 31.9, 30.4, 29.7, 29.6, 29.5, 29.4, 29.3, 29.2, 28.7, 28.4, 26.2, 26.1, 22.8, 22.7, 14.1, 14.0, 10.1. MS(ESI): $C_{81}H_{113}O_8N_2$ $[M+H]^+$ *m/z* calcd. 1241.84914; found 1241.84849.

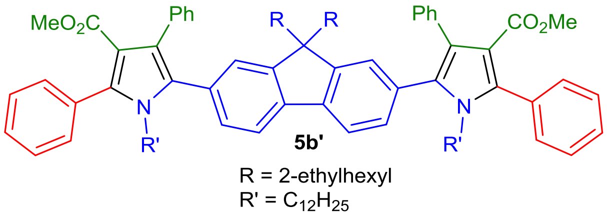

5b': Ratio of major to minor regioisomers (major shown): ~14.5:1. ^{1}H NMR (500 MHz, $CDCl_3$) δ 7.65 – 7.33 (m, 12H), 7.32 – 7.24 (m, 2H), 7.20 (d, *J* = 7.1 Hz, 3H), 7.16 – 6.98 (m, 6H), 3.88 – 3.66 (m, 4H), 3.42 (s, 6H), 2.02 – 1.81 (m, 4H), 1.43 – 0.76 (m, 60H), 0.71 (t, *J* = 7.0 Hz, 6H), 0.60 (m, 4H), 0.52 – 0.33 (m, 6H). ^{13}C NMR (125 MHz, $CDCl_3$) δ 165.8, 151.2, 140.1, 138.2, 135.3, 132.7, 132.6, 130.7, 130.4, 128.5, 128.2, 128.0, 127.1, 126.8, 126.3, 125.8, 124.3, 119.7, 112.7, 54.5, 50.6, 46.0, 45.0, 34.9, 33.2, 33.1, 31.9, 30.6, 29.6, 29.4, 29.3, 29.2, 28.8, 28.2, 27.0, 26.3, 26.0, 25.9, 22.8, 22.7, 14.1, 10.2. MS(ESI): $C_{89}H_{117}O_4N_2$ $[M+H]^+$ *m/z* calcd. 1277.90079; found 127.89813.

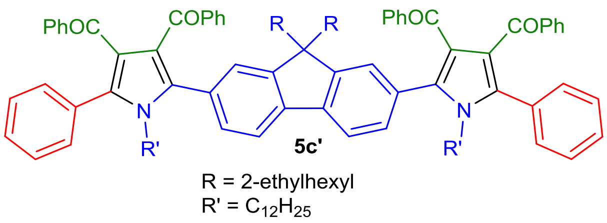

5c': ^{1}H NMR (500 MHz, $CDCl_3$) δ 7.71 (d, *J* = 7.8 Hz, 2H), 7.54 (m, 2H), 7.48 (m, 4H), 7.45 – 7.36 (m, 10H), 7.36 – 7.27 (m, 6H), 7.27 – 7.17 (m, 4H), 7.14

– 6.99 (m, 8H), 3.91 (m, 4H), 2.02 – 1.82 (m, 4H), 1.40 – 1.10 (m, 26H), 1.07 (m, 3H), 1.00 (m, 3H), 0.94 – 0.65 (m, 22H), 0.59 (m, 12H), 0.40 – 0.24 (m, 6H). ^{13}C NMR (125 MHz, $CDCl_3$) δ 192.1, 192.0, 151.0, 140.8, 139.5, 137.4, 136.6, 131.7, 131.4, 131.1, 130.9, 130.3, 129.3, 128.9, 128.4, 128.2, 127.7, 127.6, 126.2, 124.2, 123.5, 119.6, 54.9, 45.4, 45.1, 34.6, 33.4, 31.9, 30.3, 29.6, 29.4, 29.3, 29.2, 28.7, 28.2, 26.2, 22.7, 14.1, 14.0, 9.9. MS(ESI): $C_{101}H_{121}O_4N_2$ $[M+H]^+$ *m/z* calcd. 1425.93209; found 1425.93277.

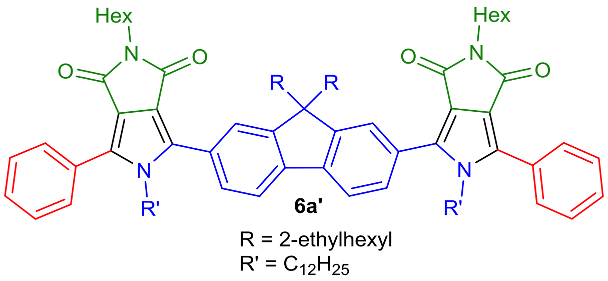

6a': ^{1}H NMR (500 MHz, $CDCl_3$) δ 7.88 (d, *J* = 7.8 Hz, 2H), 7.75 – 7.60 (m, 8H), 7.53 (t, *J* = 7.5 Hz, 4H), 7.46 (t, *J* = 7.3 Hz, 2H), 4.31 – 4.13 (m, 4H), 3.55 (t, *J* = 7.2 Hz, 4H), 2.23 – 2.07 (m, 4H), 1.70 – 1.54 (m, 4H), 1.21 (m, 40H), 1.08 – 0.94 (m, 6H), 0.86 (m, 32H), 0.75 – 0.68 (m, 1H), 0.62 (m, 5H), 0.53 (m, 6H). ^{13}C NMR (125 MHz, $CDCl_3$) δ 164.7, 164.2, 151.5, 141.4, 134.9, 134.6, 129.6, 129.4, 129.1, 128.9, 128.7, 128.1, 124.6, 120.5, 118.8, 118.7, 55.4, 46.5, 46.5, 45.4, 45.3, 37.8, 34.8, 34.7, 33.4, 33.3, 31.9, 31.6, 31.5, 30.2, 29.7, 29.6, 29.5, 29.4, 29.3, 29.2, 28.7, 28.2, 28.1, 26.6, 26.5, 25.9, 25.3, 22.8, 22.7, 22.6, 22.5, 14.1, 14.0, 13.9, 10.3, 10.2. MS(ESI): $C_{89}H_{127}O_4N_4$ $[M+H]^+$ *m/z* calcd. 1315.98518; found 1315.98105.

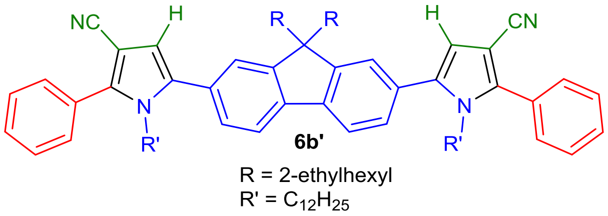

6b': Ratio of major to minor regioisomers (major shown): ~20:1. ^{1}H NMR (400 MHz, $CDCl_3$) δ 7.89 (d, *J* = 7.6 Hz, 2H), 7.67 – 7.33 (m, 14H), 6.52 (s, 2H), 4.04 (m, 4H), 2.30 – 1.96 (m, 4H), 1.23 (m, 24H), 1.09 – 0.74 (m, 26H), 0.67 (m, 6H), 0.54 (m, 6H). ^{13}C NMR (125 MHz, $CDCl_3$) δ 151.7, 142.5, 141.2, 136.3, 132.2, 129.3, 128.8, 128.7, 128.2, 125.1, 124.9, 120.6, 117.1, 112.0, 93.1, 55.3, 45.8, 45.5, 34.8, 33.5, 33.4, 31.9, 30.2, 29.7, 29.6, 29.4, 29.3, 29.2, 28.7, 28.3, 26.6, 26.5, 26.0, 22.7, 14.1, 14.0, 10.2. MS(ESI): $C_{75}H_{102}N_4Na$ $[M+Na]^+$ *m/z* calcd.1081.79967; found 1081.79877.

Typical procedure for the synthesis of poly-Münchnones 4a-e.

4a: In a glovebox, diimine **2a** (78.1 mg, 0.100 mmol) and terephtaloyl chloride **1a** (20.3 mg, 0.100 mmol) were dissolved with 0.5 mL of THF in a 5 mL vial. *N*,*N*-diisopropylethylamine (51.7 mg, 70.0 µL, 0.400 mmol) and $Pd[P(o\text{-tol})_3]_2$ (7.2 mg, 0.010 mmol) were added together with 1.0 mL of THF and 0.5 mL of MeCN. The vial equipped with a stir bar was installed in a 40 mL Parr steel autoclave. The vessel was charged with CO (20 bar) then heated at 45 °C in oil bath for 64 hours. The CO was evacuated, and the vessel was brought back into a glovebox. **4a** can be isolated if required by tituration and washing of the dark reaction slurry with acetonitrile (5 x 2 mL), to provide **4a** as a sparingly soluble purple/black solid. However, it is often more efficient and convenient to directly convert this product into polypyrrole **5a** by reaction with DMAD (see section VIII for detailed workup procedure) for yield and molecular weight determination: 73% yield. GPC: M_n = 22.7 kDa, PDI = 2.5. PolyMünchnone **4a** used for formation of polymers **5b, 5c,** and **6-8** was prepared in more moderate molecular weights (t = 30 h) to ensure complete product solubility for quantitative yield analysis.

4b: As above from **2a** (78.1 mg, 0.100 mmol), thiophene-2,5-dicarbonyl dichloride **1c** (20.9 mg, 0.10 mmol), *N*,*N*-diisopropylethylamine (51.7 mg, 70.0 µL, 0.400 mmol), $Pd[P(o\text{-tol})_3]_2$ (7.2 mg, 0.010 mmol), CO (20 bar), THF/MeCN (1.9/0.6 mL), 45 ^{o}C for 36 hours. (74% yield). GPC: M_n = 10.8 kDa, PDI = 2.8.

4c: As above from **2a** (78.1 mg, 0.100 mmol), furan-2,5-dicarbonyl dichloride **1b** (19.3 mg, 0.10 mmol), *N*,*N*-diisopropylethylamine (51.7 mg, 70.0 µL, 0.400 mmol), $Pd[P(o\text{-tol})_3]_2$ (7.2 mg, 0.010 mmol), CO (20 bar), THF/MeCN (1.5/0.5 mL), 45 ^{o}C for 64 hours. (78% yield). GPC: M_n = 12.5 kDa, PDI = 2.6.

4d: As above from **2a** (78.1 mg, 0.100 mmol), 2,3-dihydrothieno[3,4-*b*][1,4]dioxine-5,7-dicarbonyl dichloride **1d** (26.7 mg, 0.10 mmol), *N*,*N*-diisopropylethylamine (51.7 mg, 70.0 µL, 0.400 mmol), $Pd[P(o\text{-tol})_3]_2$ (7.2 mg, 0.010 mmol), CO (20 bar), THF/MeCN (1.5/0.5 mL), 45 ^{o}C for 64 hours. (76% yield). GPC: M_n = 12.8 kDa, PDI = 2.6.

4e: As above from **2b** (67.0 mg, 0.100 mmol), terephthaloyl chloride **1a** (20.3 mg, 0.10 mmol), *N*,*N*-diisopropylethylamine (51.7 mg, 70.0 µL, 0.400 mmol), Pd[P(*o*-tol)$_3$]$_2$ (7.2 mg, 0.010 mmol), CO (20 bar), THF/MeCN (1.9/0.6 mL), 45 °C for 21 hours. (55% yield). GPC: M_n = 9.4 kDa, PDI = 2.5.

Synthesis of end-capped poly-Münchnones. For quantitative spectroscopic analysis, imine end capped polymers **4a-e'** were prepared as described below:

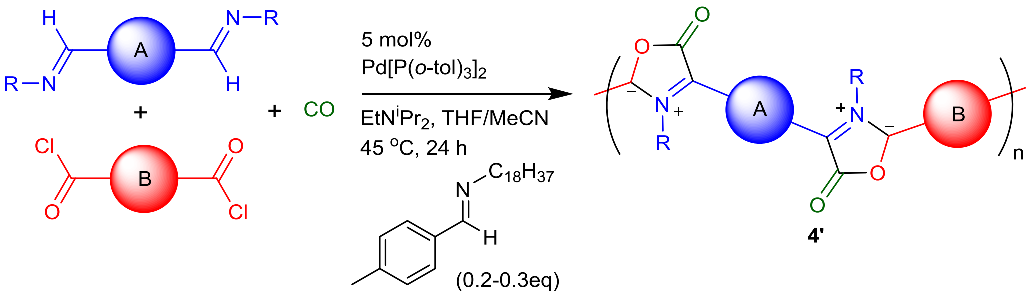

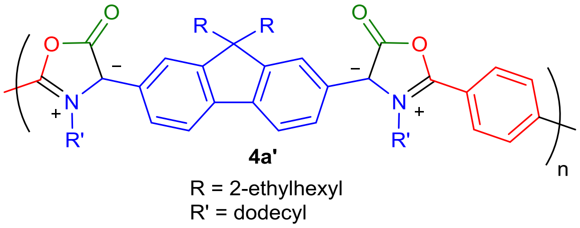

4a': In a glovebox, diimine **2a** (62.5 mg, 0.080 mmol) and (*p*-tolyl)HC=N(octadecyl) (12.7 mg, 0.04 mmol) were dissolved with 0.4 mL of THF in a 5 mL vial. terephthaloyl chloride (20.3 mg, 0.100 mmol) was added. *N*,*N*-diisopropylethylamine (51.7 mg, 70.0 µL, 0.400 mmol) and Pd[P(*o*-tol)$_3$]$_2$ (7.2 mg, 0.010 mmol) were added together with 1.1 mL of THF and 0.5 mL of MeCN. The vial equipped with a stir bar was installed in a 40 mL Parr steel autoclave. The vessel was charged with CO (20 bar) then heated at 45 °C in oil bath for 24 hours. The CO was evacuated, and the vessel was brought back into a glovebox. THF (~1 mL) was added to dissolve the purple residue. This darkly coloured solution was added dropwise to acetonitrile (16 mL) in a 20 mL capacity scintillation vial. The slurry thus formed was cooled to -35 °C overnight and subsequently centrifuged. The liquid layer was decanted and the solid residue triturated with acetonitrile repeatedly (3 x 2 mL). The solid product was dried *in vacuo* for 18 hours to give 80.7 mg of **4a'** (86% yield). GPC: M_n = 6.7 kDa, PDI = 2.6. ^{1}H NMR (400 MHz, CDCl$_3$) δ 8.31 – 7.25 (m, 10H), 4.40 – 4.31 (br m, 4H), 2.39 (s, 0.77H, *p*-tolyl end group), 2.09 (br m, 4H), 1.63 (br m, 4H), 1.34 – 0.54 (m, 72H). ^{13}C NMR (125 MHz, CDCl$_3$) 160.6, 151.4, 139.6, 137.3,

129.7, 128.8, 127.5, 125.0, 123.5, 120.2, 98.7, 98.3, 55.1, 47.7, 45.2, 34.7, 33.6, 31.9, 31.8, 29.6, 29.6, 29.5, 29.4, 29.4, 29.3, 28.8, 28.6, 28.2, 26.6, 26.0, 14.1, 14.0, 10.2, 10.2. IR (film): ν_{CO} = 1714 cm^{-1}, ν_{CN} = 1553 cm^{-1}. See Supplementary Figure 10 for ^{1}H and ^{13}C NMR spectra.

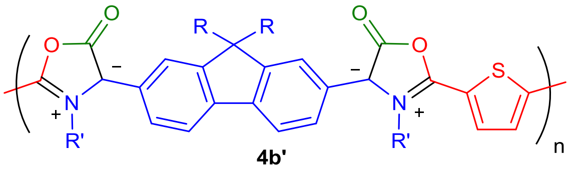

4b'

R = 2-ethylhexyl
R' = dodecyl

4b': As above from **2a** (54.7 mg, 0.07 mmol), thiophene-2,5-dicarbonyl dichloride, 20.9 mg, 0.1 mmol), (*p*-tolyl)HC=N(octadecyl) (22.3 mg, 0.060 mmol), *N*,*N*-diisopropylethylamine (51.7 mg, 70.0 µL, 0.400 mmol), and Pd[P(*o*-tol)$_3$]$_2$ (7.2 mg, 0.010 mmol). The reaction was heated to 45 °C for 24 hours before isolation. 89 mg (89% yield). GPC: M_n = 6.8 kDa, PDI = 2.9. ^{1}H NMR (400 MHz, $CDCl_3$) 7.76 – 7.05 (m, 8H), 4.37 – 4.26 (m, br, 4H), 2.38 (s, 0.96H, *p*-tolyl end group), 2.03 – 0.86 (m, 80H). ^{13}C NMR could not be obtained due to insufficient solubility of this polymer. Structure determined by conversion to polypyrrole **5g**. See Supplementary Figure 11 for ^{1}H NMR spectra.

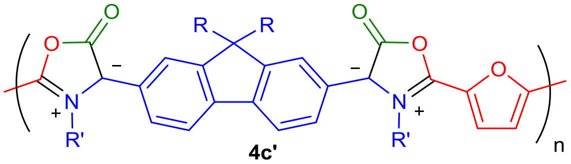

4c'

R = 2-ethylhexyl
R' = dodecyl

4c': As above from **2a** (54.7 mg, 0.07 mmol), furan-2,5-dicarbonyl dichloride, 19.3 mg, 0.1 mmol), (*p*-tolyl)HC=N(octadecyl) (22.3 mg, 0.060 mmol), *N*,*N*-diisopropylethylamine (51.7 mg, 70.0 µL, 0.400 mmol), and Pd[P(*o*-tol)$_3$]$_2$ (7.2 mg, 0.010 mmol). The reaction was heated to 45 °C for 24 hours before isolation. 90 mg (90% yield). GPC: M_n = 5.7 kDa, PDI = 2.6. ^{1}H NMR (400 MHz, $CDCl_3$) δ 7.77 – 6.95 (m, 8H), 4.46 – 4.34 (br m, 4H), 2.38 (s, 1.30H, *p*-tolyl end group), 2.03 – 1.62 (m, 4H), 1.24 – 0.49 (m, 76H). ^{13}C NMR (125 MHz, $CDCl_3$) δ 159.8, 151.4, 139.3, 137.4, 131.6, 129.6 128.5, 126.8, 126.0, 124.4, 122.9, 120.3, 114.1, 98.5, 98.1, 55.1, 47.3, 46.8, 45.2, 34.6, 33.5, 31.9, 28.8, 28.1, 22.6, 21.2, 14.0, 10.2. See Supplementary Figure 12 for ^{1}H and ^{13}C NMR spectra.

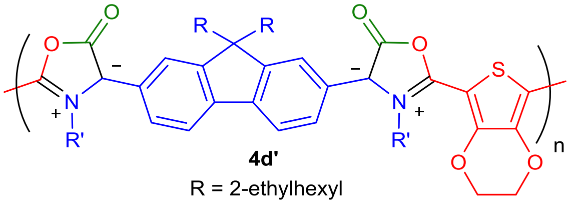

4d'

R = 2-ethylhexyl
R' = dodecyl

4d': As above from **2a** (54.7 mg, 0.07 mmol), 2,3-dihydrothieno[3,4-

b][1,4]dioxine-5,7-dicarbonyl dichloride, 26.7 mg, 0.1 mmol), (*p*-tolyl)HC=N(octadecyl) (22.3 mg, 0.060 mmol), *N*,*N*-diisopropylethylamine (51.7 mg, 70.0 µL, 0.400 mmol), and Pd[P(*o*-tol)$_3$]$_2$ (7.2 mg, 0.010 mmol). The reaction was heated to 45 °C for 24 hours before isolation. 92 mg (87% yield). GPC: M_n = 7.7 kDa, PDI = 2.4. ^{1}H NMR (400 MHz, CDCl$_3$) δ 7.73 – 7.21 (m, 6H), 4.50 – 4.27 (br m, 8H), 2.36 (s, 1.30H, *p*-tolyl end group), 2.02 (s, br, 2H), 1.61 (s, br, 2H), 1.24 – 0.53 (m, 76H). ^{13}C NMR (125 MHz, CDCl$_3$) δ 160.7, 160.6, 151.3, 139.5, 136.9, 134.2, 133.8, 129.6, 128.3, 126.6, 125.1, 122.9, 120.1, 64.9, 55.0, 47.5, 45.2, 34.7, 33.5, 31.8, 29.6, 28.1, 26.2, 22.6, 21.2, 14.1, 10.2. See Supplementary Figure 13 for ^{1}H and ^{13}C NMR spectra.

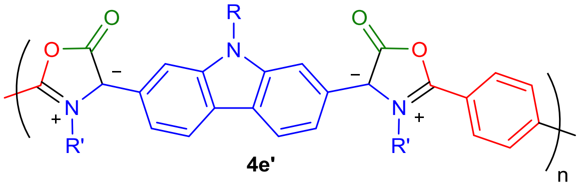

R = 2-ethylhexyl
R' = dodecyl

4e': As above from **2b** (46.9 mg, 0.07 mmol), terephthaloyl chloride **1a** (20.3 mg, 0.1 mmol), (*p*-tolyl)HC=N(octadecyl) (22.3 mg, 0.060 mmol), *N*,*N*-diisopropylethylamine (51.7 mg, 70.0 µL, 0.400 mmol), and Pd[P(*o*-tol)$_3$]$_2$ (7.2 mg, 0.010 mmol). The reaction was heated to 45 °C for only 8 hours before isolation. 78 mg (86% yield). GPC: M_n = 4.5 kDa, PDI = 2.6. ^{1}H NMR (400 MHz, CDCl$_3$) δ 8.45 – 7.24 (m, 10H), 4.43 – 4.28 (br m, 6H), 2.38 (s, 1.42H, *p*-tolyl end group), 1.68 – 0.85 (m, 61H). ^{13}C NMR (125 MHz, CDCl$_3$) δ 160.6, 139.0, 137.2, 129.6, 128.7, 127.3, 125.0, 123.5, 98.1, 65.9, 61.8, 53.3, 46.9, 31.8, 29.6, 29.4, 28.5, 25.9, 22.6, 21.2, 14.0, 11.0. See Supplementary Figure 14 for ^{1}H and ^{13}C NMR spectra.

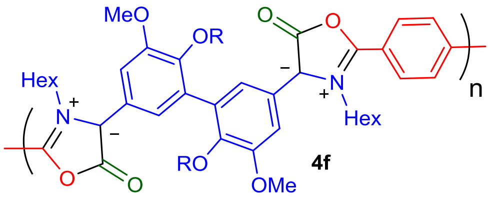

R = 3,5,5-trimethylhexyl

4f': As above from **2c** (57.7 mg, 0.08 mmol), terephthaloyl chloride **1a** (20.3 mg, 0.10 mmol), (*p*-tolyl)HC=N(octadecyl) (14.9 mg, 0.04 mmol), *N*,*N*-diisopropylethylamine (51.7 mg, 70.0 µL, 0.400 mmol), and Pd[P(*o*-tol)$_3$]$_2$ (7.2 mg, 0.010 mmol). The reaction was heated to 45 °C for only 24 hours before isolation. 72 mg (79% yield). GPC: M_n = 6.9 kDa, PDI = 1.9. ^{1}H NMR (400 MHz, CDCl$_3$) δ 7.76 (s, br, 2H),

7.26 – 7.23 (m, 4H), 6.88 (s, br, 2H), 4.36 – 3.83 (m, 14H), 2.36 (s, 0.68H, *p*-tolyl end group), 1.66 – 0.74 (m, 56H). ^{13}C NMR (125 MHz, $CDCl_3$) δ160.6, 153.4, 145.3, 139.7, 132.6, 129.7, 128.8, 127.7, 123.6, 123.0, 112.1, 97.9, 71.9, 55.8, 51.4, 47.6, 39.5, 31.8, 31.0, 30.8, 29.9, 29.6, 29.3, 28.9, 27.2, 26.0, 25.5, 22.6, 22.3, 22.2, 14.1, 13.8. See Supplementary Figure 15 for ^{1}H and ^{13}C NMR spectra.

General Procedure for the Synthesis of Polymers 5-8. In a glovebox, diacid chloride **1** (0.1 mmol) and diimine **2** (0.100 mmol) were dissolved in 0.6 mL of THF in a 5 mL vial. *N*,*N*-diisopropylethylamine (51.7 mg, 70.0 μL, 0.400 mmol) and $Pd[P(o\text{-tol})_3]_2$ (7.2 mg, 0.010 mmol) in another 1.3 mL of THF and 0.6 mL of MeCN was added to the vial. The vial equipped with a stir bar was installed in a 40 mL Parr steel autoclave. The vessel was charged with CO (20 bar) and heated at 45 °C in oil bath for 21-48 hours as noted below. The CO was evacuated, and the vessel was brought back into a glovebox. 1.5 mL of THF and the appropriate dipolarophile (together with *N*,*N*-diisopropylethylamine in some cases, as noted below) was added. The reaction mixture was stirred at room temperature or 50 °C for 16 h. 0.2 mL water was added and the mixture was extracted with *o*-dichlorobenzene using a Soxhlet extractor. The solvent was removed under vacuum and the residue was dissolved with a minimum amount of hot chloroform (~ 1mL). The concentrated solution was dripped into methanol (~20 mL) to precipitate the polymer. The suspension was centrifuged, and the methanol layer decanted. The polymer was again washed with methanol (3 x 2 mL) before drying in under vacuum at 50 °C. To quantitate the conversion of **4** into **5-8**, ^{1}H, ^{13}C NMR (See Supplementary Figures 16-31) and/or IR analysis were performed. For example, the ^{1}H NMR resonance for the NCH_2R in the Münchnone unit of **4a'** (δ 4.30-4.40 ppm) is in a unique location and can be easily monitored. This is replaced by the analogous signal in **5a** at δ 3.70 ppm. An example spectra of the conversion of **4a'** to **5a** is shown in Supplementary Figure 32. Integration shows no (< 5%) remaining Münchnone after reaction.

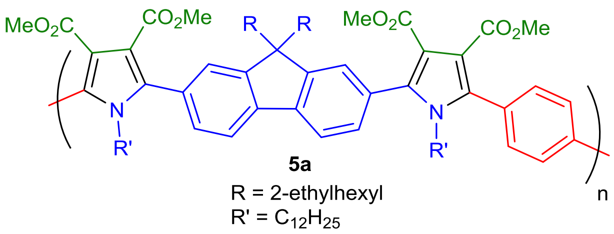

5a: As above from **2a** (78.1 mg, 0.10 mmol), terephthaloyl chloride **1a** (20.3

mg, 0.10 mmol), *N*,*N*-diisopropylethylamine (51.7 mg, 70.0 µL, 0.400 mmol), Pd[P(*o*-tol)$_3$]$_2$ (7.2 mg, 0.010 mmol), CO (20 bar), THF/MeCN (1.5/0.5 mL), 45 ^{o}C for 64 hours. The CO was evacuated, and the vessel was brought back into a glovebox. The slurry was suspended in THF (2 mL), dimethyl but-2-ynedioate (56.8 mg, 0.400 mmol) in 3mL of THF was added, and the reaction stirred at room temperature for 2 h. $CHCl_3$ (1 mL) was then added and the reaction was left to stir an additional 16 h at room temperature. After the reaction was complete, 0.2 mL of water were added and the reaction mixture was heated at 120 °C for 2 days in order to hydrolyze any remaining imine or iminium salt end groups. The mixture was then filtered over loosely packed celite. Tightly packed celite would result in a loss of yield due to the removal of highly viscous higher molecular weight polymer. The polymer was precipitated and washed with MeOH (3x2 mL), and finally filtered through an alumina plug with chloroform as a solvent. 85 mg (73% yield). GPC: M_n = 22.7 kDa, PDI = 2.5. ^{1}H NMR (400 MHz, $CDCl_3$) δ 7.83 – 7.43 (m, 10H), 3.78 – 3.61 (m, 16H), 2.04 (s, br, 4H), 1.25 – 0.52 (m, 76H). ^{13}C NMR (125 MHz,/$CDCl_3$) δ 165.1, 151.1, 141.0, 136.8, 135.6, 131.5, 130.3, 129.9, 129.6, 125.7, 119.8, 115.1, 115.0, 114.8, 54.9, 51.4, 45.5, 34.8, 33.4, 31.8, 30.6, 30.5, 29.6, 29.5, 29.4, 29.3, 29.2, 28.9, 28.3, 26.4, 26.1, 22.7, 22.6, 14.0, 10.0.

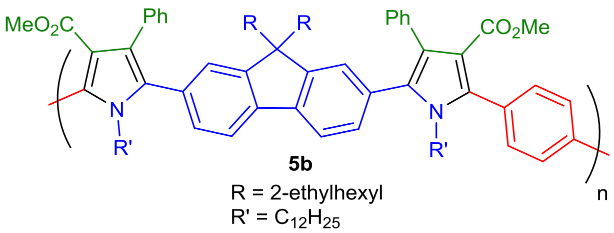

5b

5b: As above from **2a** (78.1 mg, 0.10 mmol), terephthaloyl chloride **1a** (20.3 mg, 0.10 mmol), *N*,*N*-diisopropylethylamine (51.7 mg, 70.0 µL, 0.400 mmol), Pd[P(*o*-tol)$_3$]$_2$ (7.2 mg, 0.010 mmol), CO (20 bar), THF/MeCN (1.9/0.6 mL), 45 ^{o}C for 30 hours. The cycloaddition step was carried out with methyl 3-phenylpropiolate (64.1 mg, 59.0 µL, 0.400 mmol) at 50 ^{o}C for 16 h. 115 mg (91% yield). GPC: M_n = 11.1 kDa, PDI = 2.1. ^{1}H NMR (400 MHz, $CDCl_3$) δ 8.17 – 7.12 (m, 20H), 3.85 – 3.81 (m, 4H), 3.52 – 3.43 (m, 6H), 1.91 (s, br, 4H), 1.26 – 0.46 (m, 76H). ^{13}C NMR (125 MHz,/$CDCl_3$) δ 165.5, 151.2, 140.1, 137.5, 135.1, 130.7, 130.3, 127.1, 125.8, 124.6, 119.7, 113.0, 54.4, 50.4, 45.9, 45.3, 34.9, 33.1, 31.8, 30.7, 29.6, 29.5, 29.4, 29.2, 29.0, 28.2, 22.8, 22.6, 14.0, 10.2.

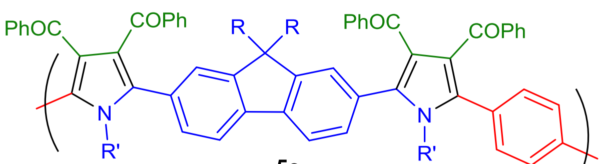

5c
R = 2-ethylhexyl
R' = $C_{12}H_{25}$

5c: As above from **2a** (78.1 mg, 0.10 mmol), terephthaloyl chloride **1a** (20.3 mg, 0.10 mmol), *N*,*N*-diisopropylethylamine (51.7 mg, 70.0 µL, 0.400 mmol), Pd[P(*o*-tol)$_3$]$_2$ (7.2 mg, 0.010 mmol), CO (20 bar), THF/MeCN (1.9/0.6 mL), 45 ^{o}C for 30 hours. The cycloaddition step was carried out with 1,4-diphenylbut-2-yne-1,4-dione (93.7 mg, 0.400 mmol) at room temperature for 16 h. 116 mg (82% yield). GPC: M_n = 10.6 kDa, PDI = 1.9. ^{1}H NMR (400 MHz, $CDCl_3$) δ 8.06 – 7.03 (m, 30H), 3.85 (s, br, 4H), 2.03 – 1.88 (m, 4H), 1.15 – 0.34 (m, 76H). ^{13}C NMR (125 MHz,/$CDCl_3$) δ 191.8, 151.1, 140.7, 139.3, 139.2, 139.1, 137.1, 136.9, 136.3, 131.8, 131.5, 131.2, 130.8, 129.2, 128.9, 127.7, 127.6, 126.0, 124.4, 124.3, 124.2, 123.8, 123.8, 119.7, 54.9, 45.3, 34.6, 33.4, 31.8, 30.4, 29.6, 29.5, 29.4, 29.3, 29.0, 28.1, 26.3, 26.1, 22.6, 14.1, 14.0, 13.9, 9.9.

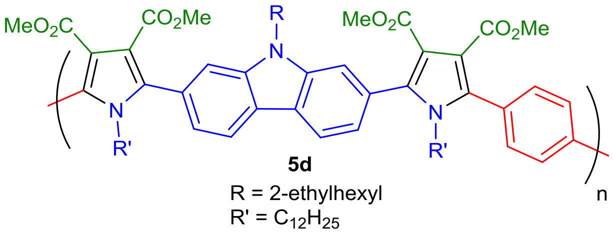

5d
R = 2-ethylhexyl
R' = $C_{12}H_{25}$

5d: As above from **2b** (67.0 mg, 0.100 mmol), terephthaloyl chloride **1a** (20.3 mg, 0.10 mmol), *N*,*N*-diisopropylethylamine (51.7 mg, 70.0 µL, 0.400 mmol), Pd[P(*o*-tol)$_3$]$_2$ (7.2 mg, 0.010 mmol), CO (20 bar), THF/MeCN (1.9/0.6 mL), 45 ^{o}C for 21 hours. The cycloaddition step was carried out with dimethyl but-2-ynedioate (56.8 mg, 50.0 µL, 0.400 mmol) at room temperature for 16 h. 53 mg (51% yield). GPC: M_n = 9.4 kDa, PDI = 2.5. ^{1}H NMR (400 MHz, $CDCl_3$) δ 8.18 – 7.26 (m, 10H), 4.21 – 3.49 (m, 18H), 1.58 – 0.84 (m, 61H). ^{13}C NMR (125 MHz, $CDCl_3$) δ 187.2, 164.1, 138.0, 129.5, 127.1, 121.6, 120.1, 111.7, 53.9, 47.9, 40.3, 39.4, 37.3, 33.6, 33.6, 31.8, 30.1, 30.1, 29.6, 29.6, 29.3, 28.9, 26.9, 26.6, 22.9, 22.6, 14.2, 14.1, 13.9, 10.8.

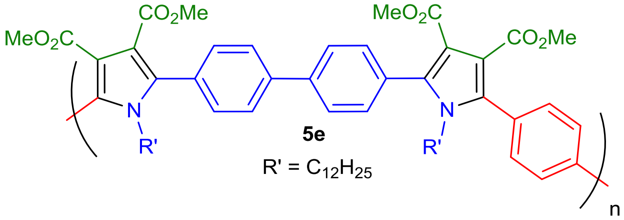

5e
R' = $C_{12}H_{25}$

5e: As above from 1,1'-([1,1'-biphenyl]-4,4'-diyl)bis(N-dodecylmethanimine) (54.4 mg, 0.100 mmol), terephthaloyl chloride **1a** (20.3

mg, 0.10 mmol), *N*,*N*-diisopropylethylamine (51.7 mg, 70.0 µL, 0.400 mmol), Pd[P(*o*-tol)$_3$]$_2$ (7.2 mg, 0.010 mmol), CO (20 bar), THF/MeCN (1.9/0.6 mL), 45 ^{o}C for 48 hours. The cycloaddition step was carried out with dimethyl but-2-ynedioate (56.8 mg, 50.0 µL, 0.400 mmol) at room temperature for 16 h. 55 mg (55% yield). GPC: M_n = 8.2 kDa, PDI = 2.1. ^{1}H NMR (400 MHz, CDCl$_3$) δ 8.20 – 7.53 (m, 12H), 3.89 – 3.47 (m, 16H), 1.83 – 0.83 (m, 46H). ^{13}C NMR (75 MHz, CDCl$_3$) δ 165.2, 131.0, 130.4, 130.2, 126.9, 54.0, 51.7, 37.3, 37.0, 33.6, 31.8, 30.1, 30.0, 29.7, 29.5, 29.2, 28.7, 27.0, 26.6, 26.2, 26.1, 22.6, 22.3, 19.7, 14.1.

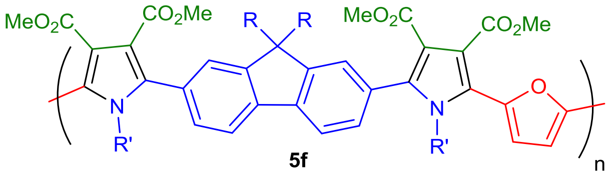

5f
R = 2-ethylhexyl
R' = $C_{12}H_{25}$

5f: As above from **2a** (78.1 mg, 0.100 mmol), furan-2,5-dicarbonyl dichloride **1b** (19.3 mg, 0.10 mmol), *N*,*N*-diisopropylethylamine (51.7 mg, 70.0 µL, 0.400 mmol), Pd[P(*o*-tol)$_3$]$_2$ (7.2 mg, 0.010 mmol), CO (20 bar), THF/MeCN (1.5/0.5 mL), 45 ^{o}C for 64 hours. The CO was evacuated, and the vessel was brought back into a glovebox. The slurry was suspended in THF (2 mL), dimethyl but-2-ynedioate (56.8 mg, 0.400 mmol) in 3mL of THF was added, and the reaction was stirred at room temperature for 2 h. CHCl$_3$ (1 mL) was then added and the reaction was left to stir an additional 16 h at room temperature. After the reaction was complete, 0.2 mL of water were added and the reaction mixture was heated at 120° for 2 days in order to hydrolyze any remaining imine or iminium salt end groups. The mixture was then filtered over loosely packed celite. Tightly packed celite would result in a loss of yield due to the removal of highly viscous higher molecular weight polymer. The polymer was precipitated and washed with MeOH (3x2 mL) and finally filtered through an alumina plug with chloroform as a solvent. 90 mg (78% yield). GPC: M_n = 12.5 kDa, PDI = 2.6. ^{1}H NMR (400 MHz, CDCl$_3$) δ 7.95 – 6.31 (m, 8H), 3.91 – 3.43 (m, 16H), 2.03 (s, br, 4H), 1.38 – 0.51 (m, 76H). ^{13}C NMR (75 MHz, CDCl$_3$) δ 165.3, 164.4, 151.1, 144.2, 141.1, 138.4, 129.9, 129.4, 125.6, 124.3, 119.8, 117.2, 114.5, 113.5, 109.6, 55.0, 51.9, 51.3, 45.9, 34.8, 33.4, 31.8, 30.7, 29.6, 29.6, 29.3, 28.3, 26.4, 26.1, 22.6, 14.0, 10.0.

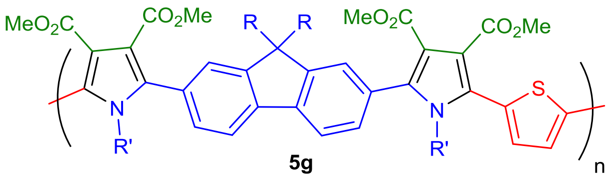

5g
R = 2-ethylhexyl
R' = $C_{12}H_{25}$

5g: As above from **2a** (78.1 mg, 0.100 mmol), thiophene-2,5-dicarbonyl dichloride **1c** (20.9 mg, 0.10 mmol), *N*,*N*-diisopropylethylamine (51.7 mg, 70.0 µL, 0.400 mmol), $Pd[P(o\text{-tol})_3]_2$ (7.2 mg, 0.010 mmol), CO (20 bar), THF/MeCN (1.9/0.6 mL), 45 ^{o}C for 36 hours. The cycloaddition step was carried out with dimethyl but-2-ynedioate (56.8 mg, 50.0 µL, 0.400 mmol) at room temperature for 16 h. 90 mg (74% yield). GPC: M_n = 10.8 kDa, PDI = 2.8. ^{1}H NMR (400 MHz, $CDCl_3$) δ 7.96 – 6.20 (m, 8H), 3.84 – 3.45 (m, 16H), 2.04 (s, br, 4H), 1.62 – 0.51 (m, 76H). ^{13}C NMR (125 MHz, $CDCl_3$) δ 165.1, 164.6, 151.2, 141.1, 137.8, 133.2, 129.9, 129.5, 126.7, 125.6, 119.8, 117.3, 114.8, 55.0, 51.7, 51.4, 45.5, 40.2, 34.9, 33.4, 31.8, 30.9, 29.6, 29.6, 29.5, 29.4, 29.2, 29.1, 28.3, 27.8, 22.8, 22.6, 14.0, 10.0.

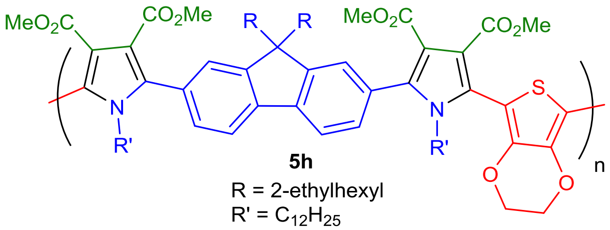

5h
R = 2-ethylhexyl
R' = $C_{12}H_{25}$

5h: As above from **2a** (78.1 mg, 0.100 mmol), 2,3-dihydrothieno[3,4-*b*][1,4]dioxine-5,7-dicarbonyl dichloride **1d** (26.7 mg, 0.10 mmol), *N*,*N*-diisopropylethylamine (51.7 mg, 70.0 µL, 0.400 mmol), $Pd[P(o\text{-tol})_3]_2$ (7.2 mg, 0.010 mmol), CO (20 bar), THF/MeCN (1.5/0.5 mL), 45 ^{o}C for 64 hours. The CO was evacuated, and the vessel was brought back into a glovebox. The slurry was suspended in THF (2 mL), dimethyl but-2-ynedioate (56.8 mg, 0.400 mmol) in 3mL of THF was added, and the reaction was stirred at room temperature for 2 h. $CHCl_3$ (1 mL) was then added and the reaction was left to stir 16 h at room temperature. After the reaction was complete, 0.2 mL of water were added and the reaction mixture was heated at 120 °C for 2 days in order to hydrolyze any remaining imine or iminium salt end groups. The mixture was then filtered over loosely packed celite. Tightly packed celite would result in a loss of yield due to the removal of highly viscous higher molecular weight polymer. The polymer was precipitated and washed with MeOH (3x2mL) and finally filtered through an alumina plug with chloroform as a solvent. 94 mg (76% yield). GPC: M_n = 12.8 kDa, PDI = 2.6. ^{1}H NMR (400 MHz, $CDCl_3$) δ 7.95 – 7.40 (m, 6H), 4.28 (s, br, 4H), 3.88 – 3.42 (m, 16H), 2.03 (s,

br, 4H), 1.61 – 0.51 (m, 76H). ^{13}C NMR (75 MHz, $CDCl_3$) δ 164.7, 151.0, 141.0, 139.7, 137.8, 129.9, 129.5, 125.6, 124.3, 119.7, 117.5, 115.2, 106.9, 64.6, 54.9, 51.6, 51.4, 45.5, 34.8, 33.4, 31.8, 30.7, 29.6, 29.5, 29.3, 28.3, 26.7, 26.1, 22.8, 22.6, 14.1, 10.0.

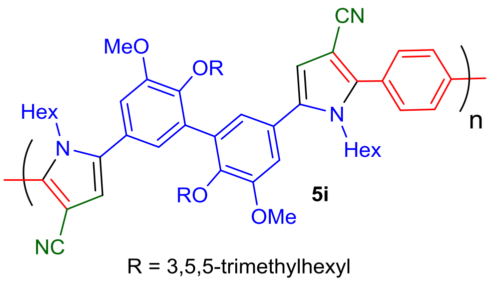

R = 3,5,5-trimethylhexyl

5i: As above from **2c** (72.1 mg, 0.100 mmol), terephthaloyl chloride **1a** (20.3 mg, 0.10 mmol), *N*,*N*-diisopropylethylamine (51.7 mg, 70.0 µL, 0.400 mmol), Pd[P(*o*-tol)$_3$]$_2$ (7.2 mg, 0.010 mmol), CO (20 bar), THF/MeCN (0.75/0.25 mL), 45 ^{o}C for 40 hours. The cycloaddition step was carried out with 2-chloroacrylonitrile (35.0 mg, 32.0 µL, 0.400 mmol) and *N*,*N*-diisopropylethylamine (51.7 mg, 70.0 µL, 0.400 mmol) at room temperature for 16 h. 75 mg (82% yield). GPC: M_n = 8.5 kDa, PDI = 1.6. ^{1}H NMR (400 MHz, $CDCl_3$) δ 7.46 (s, br, 4H), 7.11 – 7.04 (m, br, 4H), 6.52 (s, 2H), 4.12 – 3.46 (m, 14H), 1.25 – 0.68 (m, 56H). ^{13}C NMR (125 MHz, $CDCl_3$) δ 153.2, 147.0, 142.8, 135.5, 132.8, 131.8, 129.2, 124.6, 124.2, 117.3, 113.1, 112.2, 92.9, 71.9, 56.0, 51.3, 46.0, 39.5, 31.0, 30.8, 30.1, 29.9, 29.6, 27.2, 26.0, 25.6, 22.3, 22.2, 13.8.

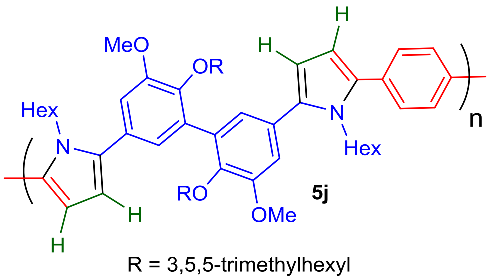

R = 3,5,5-trimethylhexyl

5j: As above from **2c** (72.1 mg, 0.100 mmol), terephthaloyl chloride **1a** (20.3 mg, 0.10 mmol), *N*,*N*-diisopropylethylamine (51.7 mg, 70.0 µL, 0.400 mmol), Pd[P(*o*-tol)$_3$]$_2$ (7.2 mg, 0.010 mmol), CO (20 bar), THF/MeCN (0.75/0.25 mL), 45 ^{o}C for 40 hours. The cycloaddition step was carried out with triphenyl(vinyl)phosphonium bromide (147.7 mg, 0.400 mmol) and *N*,*N*-diisopropylethylamine (51.7 mg, 70.0 µL, 0.400 mmol) at room temperature for 16 h. 70 mg (80% yield). GPC: M_n = 8.1 kDa, PDI = 1.3. ^{1}H NMR (400 MHz, $CDCl_3$) δ 7.46 (s, br, 4H), 7.04 – 7.00 (m, br, 4H), 6.27 – 6.26 (s, br, 4H), 4.13 – 3.48 (m, 14H), 1.25 – 0.69 (m, 56H). ^{13}C NMR (125 MHz, $CDCl_3$) 152.6, 145.5, 136.6, 136.0, 135.3, 132.9, 132.5, 128.9, 128.6, 120.9, 117.5, 112.3, 109.3, 71.7, 55.8, 51.4, 45.3, 39.5, 39.5, 30.8, 29.9, 29.6, 27.2, 26.6, 26.0, 22.3, 13.8.

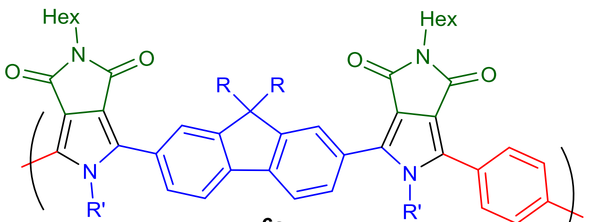

6a
R = 2-ethylhexyl
R' = $C_{12}H_{25}$

6a: As above from **2a** (78.1 mg, 0.100 mmol), terephthaloyl chloride **1a** (20.3 mg, 0.10 mmol), *N*,*N*-diisopropylethylamine (51.7 mg, 70.0 μL, 0.400 mmol), Pd[P(*o*-tol)$_3$]$_2$ (7.2 mg, 0.010 mmol), CO (20 bar), THF/MeCN (1.9/0.6 mL), 45 °C for 30 hours. The cycloaddition step was carried out with 3-bromo-1-hexyl-1H-pyrrole-2,5-dione (104.1 mg, 0.400 mmol) and *N*,*N*-diisopropylethylamine (51.7 mg, 70.0 μL, 0.400 mmol) at room temperature for 16 h. 126.8 mg (96% yield). GPC: M_n = 11.4 kDa, PDI = 2.4. ^{1}H NMR (400 MHz, $CDCl_3$) δ 8.24 – 7.70 (m, 10H), 4.31 (s, br, 4H), 3.57 (s, br, 4H), 2.48 (s, br, 4H), 1.63 (s, br, 4H), 1.29 – 0.56 (m, 94H). ^{13}C NMR (75 MHz, $CDCl_3$) δ 164.7, 164.0, 151.6, 141.4, 135.6, 133.8, 130.0, 129.7, 128.7, 128.0, 124.6, 120.6, 119.3, 119.0, 55.4, 46.9, 45.3, 37.9, 34.8, 33.3, 31.8, 31.4, 30.3, 29.6, 29.5, 29.4, 29.3, 28.8, 28.7, 28.1, 28.1, 26.6, 26.0, 22.7, 22.6, 22.5, 14.1, 14.0, 10.2, 10.2.

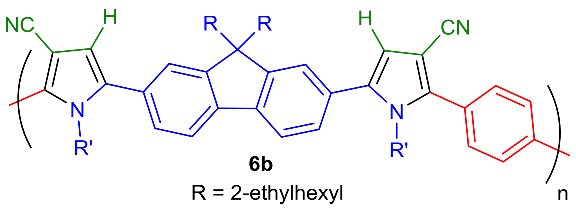

6b
R = 2-ethylhexyl
R' = $C_{12}H_{25}$

6b: As above from **2a** (78.1 mg, 0.100 mmol), terephthaloyl chloride **1a** (20.3 mg, 0.10 mmol), *N*,*N*-diisopropylethylamine (51.7 mg, 70.0 μL, 0.400 mmol), Pd[P(*o*-tol)$_3$]$_2$ (7.2 mg, 0.010 mmol), CO (20 bar), THF/MeCN (1.9/0.6 mL), 45 °C for 30 hours. The cycloaddition step was carried out with 2-chloroacrylonitrile (35.0 mg, 0.400 mmol) and *N*,*N*-diisopropylethylamine (51.7 mg, 70.0 μL, 0.400 mmol) at room temperature for 16 h. 92.4 mg (95% yield). GPC: M_n = 9.8 kDa, PDI = 2.3. ^{1}H NMR (400 MHz, $CDCl_3$) δ 7.92 – 7.55 (m, 10H), 6.61 (s, br, 2H), 4.12 (s, br, 4H), 2.14 (s, br, 4H), 1.25 – 0.56 (m, 76H). ^{13}C NMR (75 MHz, $CDCl_3$) δ 151.7, 143.0, 141.3, 135.5, 131.9 129.4, 128.8, 128.6, 125.0, 120.6, 116.8, 112.5, 93.4, 55.3, 46.0, 45.5, 34.8, 33.5, 33.4, 31.8, 29.7, 29.5, 29.4, 29.3, 28.8, 28.3, 26.6, 26.5 26.0, 22.6, 14.1, 14.0.

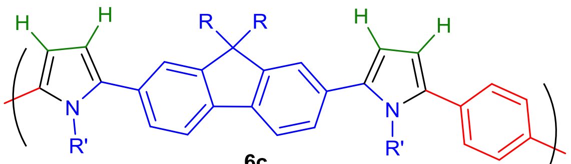

6c
R = 2-ethylhexyl
R' = $C_{12}H_{25}$

6c: As above from **2a** (78.1 mg, 0.100 mmol), terephthaloyl chloride **1a** (20.3 mg, 0.10 mmol), *N*,*N*-diisopropylethylamine (51.7 mg, 70.0 µL, 0.400 mmol), Pd[P(*o*-tol)$_3$]$_2$ (7.2 mg, 0.010 mmol), CO (20 bar), THF/MeCN (1.9/0.6 mL), 45 ^{o}C for 30 hours. The cycloaddition step was carried out with triphenyl(vinyl)phosphonium bromide (147.7 mg, 0.400 mmol) and *N*,*N*-diisopropylethylamine (51.7 mg, 70.0 µL, 0.400 mmol) at room temperature for 16 h. The product was isolated directly without Soxhlet extraction. 88 mg (95% yield). GPC: M_n = 8.1 kDa, PDI = 1.8. ^{1}H NMR (400 MHz, $CDCl_3$) δ 8.32 – 7.45 (m, 10H), 6.36 – 6.31 (m, 4H), 4.18 (s, br, 4H), 2.07 (s, br, 4H), 1.26 – 0.56 (m, 76H). ^{13}C NMR (75 MHz, $CDCl_3$) δ 151.0, 139.9, 137.5, 136.2, 132.6, 132.3, 128.8, 127.6, 124.6, 119.5, 109.5, 54.9, 45.5, 45.2, 34.7, 33.8, 33.6, 31.8, 30.6, 29.7, 29.6, 29.4, 29.4, 29.3, 28.9, 28.4, 28.3, 26.7, 26.6, 26.2, 22.7, 22.6, 14.1, 14.0, 10.3, 10.2.

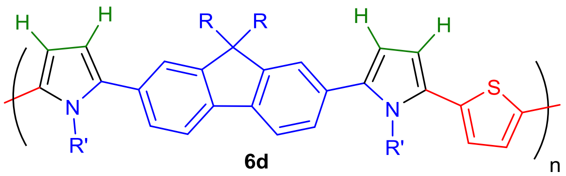

6d
R = 2-ethylhexyl
R' = $C_{12}H_{25}$

6d: As above from **2a** (78.1 mg, 0.100 mmol), thiophene-2,5-dicarbonyl dichloride **1c** (20.9 mg, 0.10 mmol), *N*,*N*-diisopropylethylamine (51.7 mg, 70.0 µL, 0.400 mmol), Pd[P(*o*-tol)$_3$]$_2$ (7.2 mg, 0.010 mmol), CO (20 bar), THF/MeCN (1.9/0.6 mL), 45 ^{o}C for 16 hours. The cycloaddition step was carried out with triphenyl(vinyl)phosphonium bromide (147.7 mg, 0.400 mmol) and *N*,*N*-diisopropylethylamine (51.7 mg, 70.0 µL, 0.400 mmol) at room temperature for 16 h. The product was isolated directly without Soxhlet extraction. 76 mg (82% yield). GPC: M_n = 6.1 kDa, PDI = 2.1. ^{1}H NMR (400 MHz, $CDCl_3$) δ 7.95 – 7.38 (m, 7H), 7.07 (s, br, 1H), 6.44 (s, br, 2H), 6.25 (s, br, 2H), 4.21 (s, br, 4H), 2.08 (s, br, 4H), 1.62 – 0.55 (m, 76H). ^{13}C NMR (125 MHz, $CDCl_3$) δ 150.9, 140.1, 137.5, 135.2, 134.6, 133.8, 132.3, 130.7, 130.3, 128.3, 128.0, 125.4, 124.8, 119.5, 110.7, 109.5, 68.1, 68.1, 54.9, 53.7, 45.4, 45.0, 42.0, 40.0, 34.7, 33.8, 33.5, 31.9, 30.9, 29.6, 29.6, 29.3, 28.3, 27.0, 26.8, 26.4, 22.7, 22.6, 14.2, 14.0, 10.2.

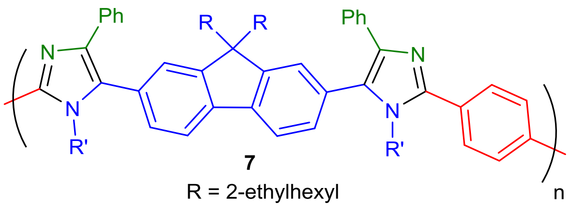

7
R = 2-ethylhexyl
R' = $C_{12}H_{25}$

7: As above from **2a** (78.1 mg, 0.100 mmol), terephthaloyl chloride **1a** (20.3 mg, 0.10 mmol), *N*,*N*-diisopropylethylamine (51.7 mg, 70.0 µL, 0.400 mmol), Pd[P(*o*-tol)$_3$]$_2$ (7.2 mg, 0.010 mmol), CO (20 bar), THF/MeCN (1.9/0.6 mL), 45 °C for 40 hours. The cycloaddition step was carried out with *N*-benzylidene-4-methylbenzenesulfonamide (259 mg, 1.0 mmol) at room temperature for 16 h. The product was isolated directly without Soxhlet extraction. Indicated by ^{1}H NMR, the isolated product contains a very small amount of impurities containing the tosyl group. 75 mg (65% yield). GPC: M_n = 9.2 kDa, PDI = 1.4. ^{1}H NMR (400 MHz, $CDCl_3$) δ 7.91 – 7.18 (m, 20H), 3.85 – 3.47 (m, 4H), 2.05 (s, br, 4H), 1.25 - 0.52 (m, 76H). ^{13}C NMR (75 MHz, $CDCl_3$) δ 152.0, 134.8, 131.2, 129.9, 129.1, 128.6, 128.2, 126.8, 125.0, 121.1, 120.6, 55.3, 49.1, 44.4, 40.1, 34.7, 33.8, 31.8, 29.6, 29.4, 28.8, 28.1, 27.0, 22.6, 14.1, 13.9, 10.3.

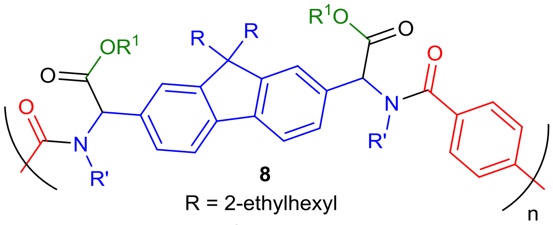

8
R = 2-ethylhexyl
R' = $C_{12}H_{25}$
R^1 = 3,5,5-trimethylhexyl

8: As above from **2a** (78.1 mg, 0.100 mmol), terephthaloyl chloride **1a** (20.3 mg, 0.10 mmol), *N*,*N*-diisopropylethylamine (51.7 mg, 70.0 µL, 0.400 mmol), Pd[P(*o*-tol)$_3$]$_2$ (7.2 mg, 0.010 mmol), CO (20 bar), THF/MeCN (1.9/0.6 mL), 45 °C for 30 hours. The crude polyMünchnone was quenched with 3,5,5-trimethylhexan-1-ol (57.7 mg, 0.400 mmol) at room temperature for 16 h and then heated at 70 °C for 1 hour. The crude mixture was concentrated and precipitated in MeOH (~ 20 mL). The slurry thus formed was then centrifuged. The liquid layer was decanted and the solid residue triturated with MeOH repeatedly (3 x 2 mL). The solid product was dried *in vacu*. 103 mg (82% yield). GPC: M_n = 11.5 kDa, PDI = 2.0. ^{1}H NMR (400 MHz, $CDCl_3$) δ 8.14 – 7.52 (m, 10H), 6.04 – 5.90 (m, 2H), 4.26 – 4.20 (m, 4H), 3.31 – 3.22 (m, 2H), 2.01 - 0.52 (m, 114H). ^{13}C NMR (75 MHz, $CDCl_3$) δ 172.1, 170.3, 151.6, 140.9, 137.6, 132.9, 128.4, 126.8, 125.8, 120.0, 65.7, 64.1, 62.4, 55.0, 50.9, 47.6, 44.9, 37.6, 34.6, 33.6, 31.8, 31.0, 29.9, 29.5, 29.4, 29.3, 29.0, 28.3, 27.2, 26.6, 26.2, 22.6, 22.5, 14.1, 13.9, 10.2, 10.0.

Quantification of cycloaddition with ^{13}C labelled 4a'.

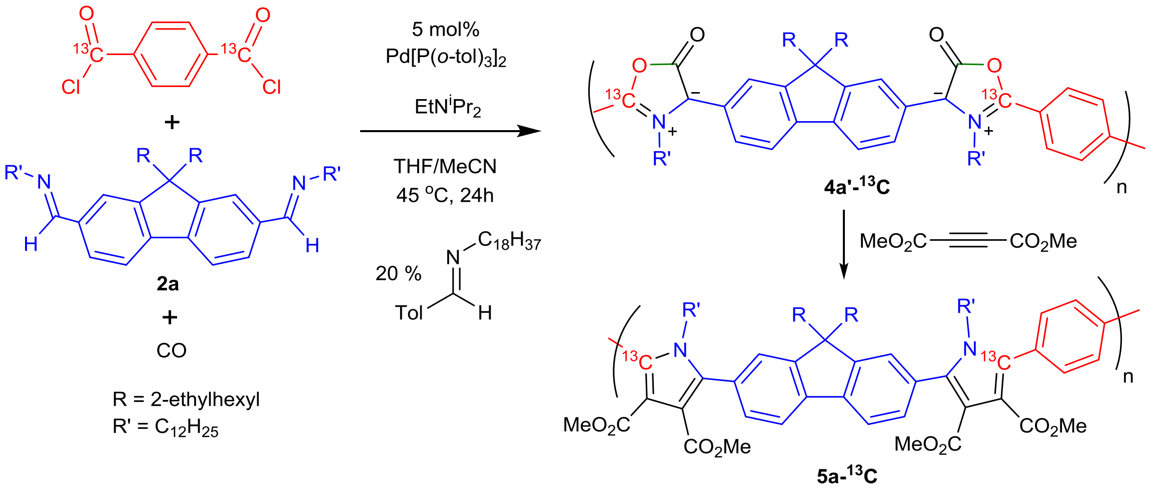

In order to quantify the post-polymerization cycloaddition of polymers **4**, ^{13}C labelled polymer **4a'-^{13}C** was generated as follows: In a glovebox, diimine **2a** (62.5 mg, 0.080 mmol) and (*p*-tolyl)HC=N(octadecyl) (12.7 mg, 0.04 mmol) were dissolved with 0.4 mL of THF in a 5 mL vial. ^{13}CO-terephthaloyl chloride (20.3 mg, 0.100 mmol) was added. *N*,*N*-diisopropylethylamine (51.7 mg, 70.0 µL, 0.400 mmol) and Pd[P(*o*-tol)$_3$]$_2$ (7.2 mg, 0.010 mmol) were added together with 1.1 mL of THF and 0.5 mL of MeCN. The vial equipped with a stir bar was installed in a 40 mL Parr steel autoclave. The vessel was charged with CO (20 bar) then heated at 45 °C in oil bath for 24 hours. The CO was evacuated, and the vessel was brought back into a glovebox. THF (~1 mL) was added to dissolve the purple residue. This darkly colored solution was added dropwise to acetonitrile (16 mL) in a 20 mL capacity scintillation vial. The slurry thus formed was cooled to -35 °C overnight and subsequently centrifuged. The liquid layer was decanted and the solid residue triturated with acetonitrile repeatedly (3 x 2 mL). The solid product was dried *in vacuo* for 18 hours to give 66.2 mg of **4a'-^{13}C** (81% yield). Polymer **5a-^{13}C** was prepared as above from **4a'-^{13}C** (20.0 mg, 0.021 mmol) and DMAD (9 mg, 0.06 mmol).

^{13}C NMR analysis of **4a'-^{13}C** (See Supplementary Figure 33) shows the incorporation of ^{13}C-label into the Münchnone unit (140 and 139 ppm) as the only enhanced signals in the aromatic region. After cycloaddition with DMAD, this signal is no longer present, and is replaced by ^{13}C enriched signals at 136 and 135 ppm, corresponding to the pyrrole

backbone carbon. No other ^{13}C labeled signals are observed in the aromatic region, suggesting the essentially quantitative conversion (>95%) of the Münchnone unit into pyrrole.

Procedure for the synthesis of 5b *via* four-component polymerization.

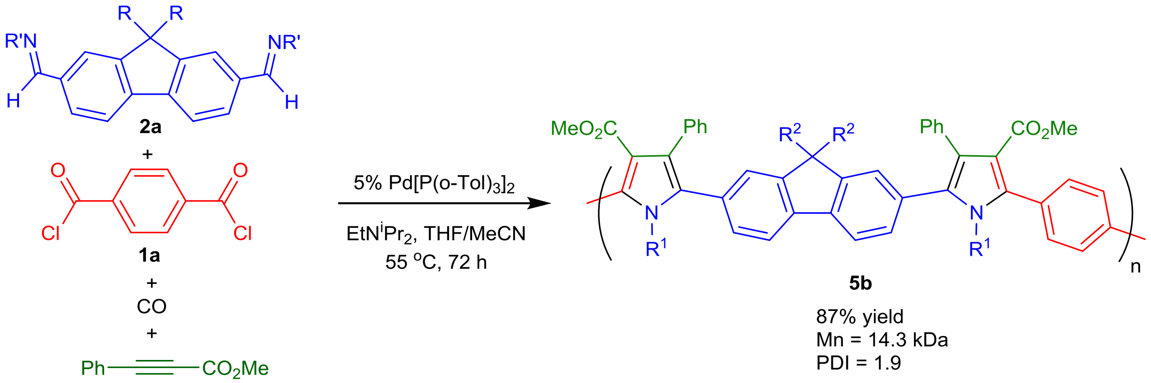

In the glovebox, diimine **2a** (78.1mg, 0.1 mmol) and diacid chloride **1a** (20.3 mg, 0.100 mmol) were dissolved with 0.25 mL of THF in a 5 mL vial. *N*,*N*-diisopropylethylamine (51.7 mg, 70.0 µL, 0.400 mmol) and Pd[P(*o*-tol)$_3$]$_2$ (7.2 mg, 0.010 mmol) together with another 0.5 mL of THF and 0.25 mL of MeCN were added to the vial. Then methyl 3-phenylpropiolate (64.1 mg, 59.0 µL, 0.400 mmol) was added to the mixture. The vial equipped with a stir bar was installed in a 40 mL Parr steel autoclave. The vessel was charged with CO (20 bar) and heated at 55 °C in oil bath for 72 hours. Afterward, the CO was evacuated and 0.2 mL of water was added. The solvent was removed under vacuum and the residue was dissolved with a minimum amount of hot chloroform (~ 1mL). The concentrated solution was dripped into methanol (~20 mL) to precipitate the polymer. The suspension was centrifuged, and the methanol layer decanted. The polymer was again washed with methanol (3 x 2 mL) before drying in under vacuum at 50 °C. Yield: 104 mg (87%). GPC: M_n = 14.3 kDa, PDI = 1.9.

Supplementary References

1. D'Andrade, B. W.; Datta, S.; Forrest, S. R.; Djurovich, P.; Polikarpov, E.; Thompson, M. E. Relationship between the ionization and oxidation potentials of molecular organic semiconductors. *Organic Electronics* **6**, 11-20 (2005).

2. Zalesskly, S. S.; Ananikov, V. P. $Pd_2(dba)_3$ as a precursor of soluble metal complexes and nanoparticles: determination of palladium active species for catalysis and synthesis. *Organometallics* **31**, 2302-2309 (2012).
3. Ogata, T.; Hartwig, J. F. Palladium-catalyzed amination of aryl and heteroaryl tosylates at room temperature. *J. Am. Chem. Soc.* **130**, 13848-13849 (2008).
4. Littke, A. F.; Fu, G. C. Heck reactions of aryl chlorides catalyzed by palladium/tri-tert-butylphosphine: (E)-2-methyl-3-phenylacrylic acid butyl ester and (E)-4-(2-phenylethenyl)benzonitrile. *Org. Synth.* **81**, 63-76 (2005).
5. Weychardt, H.; Plenio, H. Acyclic diene metathesis polymerization of divinylarenes and divinylferrocenes with grubbs-type olefin metathesis catalysts. *Organometallics.* **27**, 1479-1485 (2008).
6. Leclerc, M.; Morin, J.-F. WO2005/16882 (EP1660450 A1).
7. Gomez, M.; Gandini, A.; Silvestre, A. J. D.; Reis, B. A. Synthesis and characterization of poly(2,5-furan dicarboxylate)s based on a variety of diols. *J. Polym. Sci. A Polym. Chem.* **49**, 3759-3768 (2011).
8. Siamaki, A. R.; Sakalauskas, M.; Arndtsen, B. A. A palladium-catalyzed multicomponent coupling approach to π-conjugated oligomers: assembling imidazole-based materials from imines and acyl chlorides. *Angew. Chem. Int. Ed.* **50**, 6552-6556 (2011).
9. Zhang, J. J.; Schuster, G. B. Ylidions: a new reactive intermediate prepared by photosensitized one-electron oxidation of phenacyl sulfonium ylides. *J. Am. Chem. Soc.* **111**, 7179-7155 (1989).
10. Onimura, K.; Matsushima, M.; Yamabuki, K.; Oishi, T. Synthesis and properties of N-substituted maleimides conjugated with 1,4-phenylene or 2,5-thienylene polymers. *Polymer Journal,* **42**, 290-297 (2010).
11. Delomenede, M.; Bedos-Delval, F.; Duran, H.; Vindis, C.; Baltas, M.; Negre-Salvayre, A. Development of novel antiatherogenic biaryls: design, synthesis, and reactivity. *J. Med. Chem.* **51**, 3171-3181 (2008).
